# Supplementary material for: Engineering Genetic Predisposition in Human Neuroepithelial Stem Cells Recapitulates Medulloblastoma Tumorigenesis
Source: Cell Stem Cell. 2019 Sep 5;25(3):433–446.e7. doi: 10.1016/j.stem.2019.05.013 (PMC6731167; doi:10.1016/j.stem.2019.05.013)
Supplement: Document S1. Figures S1–S7 [file mmc1.pdf]

Cell Stem Cell, Volume *25*

Supplemental Information

Engineering Genetic Predisposition in Human Neuroepithelial Stem Cells Recapitulates Medulloblastoma Tumorigenesis

Miller Huang, Jignesh Tailor, Qiqi Zhen, Aaron H. Gillmor, Matthew L. Miller, Holger Weishaupt, Justin Chen, Tina Zheng, Emily K. Nash, Lauren K. McHenry, Zhenyi An, Fubaiyang Ye, Yasuhiro Takashima, James Clarke, Harold Ayetey, Florence M.G. Cavalli, Betty Luu, Branden S. Moriarity, Shirin Ilkhanizadeh, Lukas Chavez, Chunying Yu, Kathreena M. Kurian, Thierry Magnaldo, Nicolas Sevenet, Philipp Koch, Steven M. Pollard, Peter Dirks, Michael P. Snyder, David A. Largaespada, Yoon Jae Cho, Joanna J. Phillips, Fredrik J. Swartling, A. Sorana Morrissy, Marcel Kool, Stefan M. Pfister, Michael D. Taylor, Austin Smith, and William A. Weiss

SUPPLEMENTARY FIGURES

Figure S1. Expression of FLAG-MYCN and p53 in WTC10 *MYCN* tumors. (Related to Figure 1). Immunohistochemical staining of WTC10 *MYCN* tumors show **(A)** positive staining for FLAG (MYCN), and **(B)** negative staining for p53. Scale bars indicate 20um. **(C)** Quantitation of staining for FLAG and p53. A minimum of three fields of 400x view were analyzed per tumor, with approximately 400 cells in each field of view. Data represent mean ± SEM.

Figure S2*.* Hierarchical clustering analysis of WTC10 *MYCN* tumors (Related to Figures 1 and 2). Hierarchical clustering of transcriptomes of WTC10 *MYCN* tumors with **(A)** various pediatric brain tumors and **(B)** four major medulloblastoma subgroups showing WTC10 *MYCN* tumors resemble SHH medulloblastoma.

Figure S3. Keratinocytes from Gorlin syndrome patients can be converted to iPSC (Related to Figure 3). **(A)** Phase contrast images of keratinocytes from a healthy control (WT) and a patient with Gorlin syndrome (Gorlin 1). **(B)** Table showing the PTCH1 status in WT and Gorlin keratinocytes. **(C)** Protocol for reprogramming keratinocytes to iPSC with Sendai virus. **(D)** Both Gorlin iPSC clones expressed pluripotency markers OCT4, SOX2, and NANOG and showed typical morphology of human iPSCs. **(E)** Differentiated embryoid bodies from Gorlin 1 iPSC were evaluated for markers of the three germ layers. Images shown are (left) immunofluorescence detection for markers of the neuroectoderm (SOX1, TUJ1), mesoderm (TBRA) and endoderm (SOX17), and (right) phase contrast images. **(F)** Teratoma after injection into kidney capsules of NOD/SCID mice contain tissue of all three germ layers including neuroectodermal rosettes (ectoderm) (i), intestinal epithelium (endoderm) (ii), and mesenchymal tissue (mesoderm) (iii). **(G)** Phase contrast image of NES cells derived from Gorlin 1 iPSC, displaying typical rosette forming features of NES cells. **(H)** RT-qPCR analysis of NES cell lines derived from Gorlin 1 and Gorlin 2 iPSC show typical neuroepithelial (SOX1), rosette-stage (PLZF1, MMNR1) and hindbrain regional markers (GBX2) similar to that found in fetal hindbrain NES cells (Sai2) (Tailor et al., 2013), and low expression of radial-glial markers (GFAP, S100B). (Scale bars: D – 100um, E – 50um (top four panels), 75uM (lower two panels), G – 50um).

Figure S4. Hierarchical clustering analysis of Gorlin 1 tumors for brain tumor type and medulloblastoma subgroup (Related to Figure 4). Hierarchical clustering of transcriptomes of Gorlin 1 tumors with **(A)** pediatric brain tumors and **(B)** four major medulloblastoma subgroups. Gorlin tumors align with SHH medulloblastoma.

Figure S5. Hierarchical clustering analysis of Gorlin 1 *DDX3X* mutant and *GSE1*$^{-/-}$ tumors for medulloblastoma subgroup (Related to Figures 6 and 7). Hierarchical clustering analysis of transcriptomes comparing **(A)** Gorlin *DDX3X* mutant tumors, and **(B)** Gorlin *GSE1*$^{-/-}$ tumors with the four major subgroups of medulloblastoma.

Figure S6. Characterization of GSE1 and KDM3B expression in medulloblastoma survival and mutations generated in NES cells (Related to Figure 7). (A) Kaplan Meier curves of human medulloblastoma patients stratified by expression levels for *GSE1* or *KDM3B*. **(B)** Gorlin 1 NES cells were transduced with Cas9 and either control (Ctrl) sgRNA or sgRNA binding *GSE1* or *KDM3B*. Genomic DNA was extracted and PCR was performed using primers specific to the intended cut site on *GSE1* or *KDM3B.* PCR products were digested with Surveyor nuclease and run on a 10% TBE gel. Cleavage products confirmed indels at the desired locations. **(C)** Quantitation of the number of alleles mutated or showing a frameshift at *GSE1* and *KDM3B* (50,000 total reads per gene). **(D)** Examples from amplicon sequencing of mutations near the sgRNA target regions for *GSE1* and *KDM3B*. **(E)** Schematic of GSE1 construct showing silent mutations preventing sgRNA binding while expressing WT amino acid sequence for GSE1.

Figure S7: Copy number variations in MYCN and Gorlin 1 tumors (Related to Figure 2, and Figure 4) Representative copy number analysis of **(A)** *MYCN* tumors and **(B)** Gorlin 1 tumors showing no chromosome copy number changes.

Figure S1

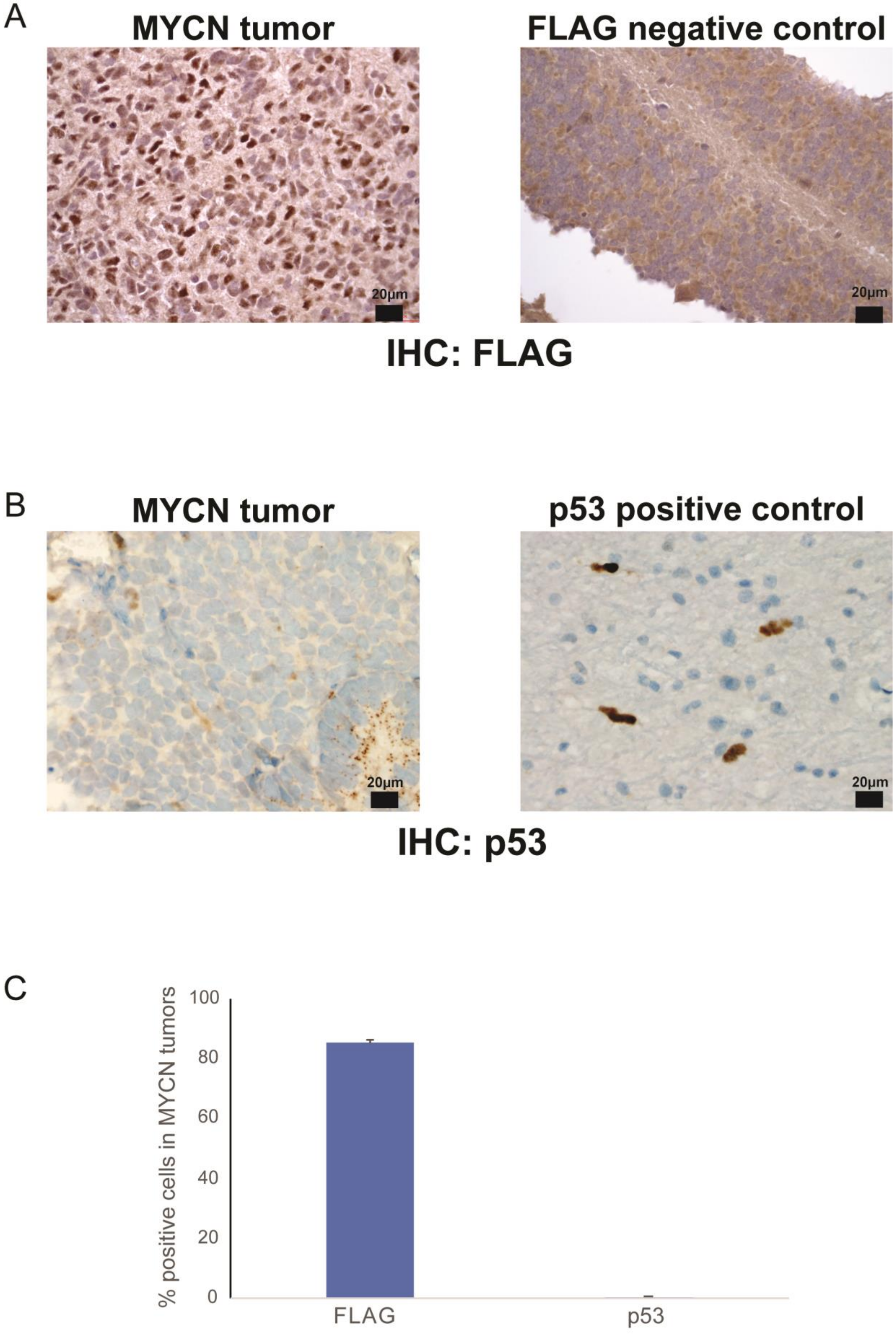
A
MYCN tumor
FLAG negative control
20µm
20µm
IHC: FLAG
B
MYCN tumor
p53 positive control
20µm
20µm
IHC: p53
C
% positive cells in MYCN tumors
100
80
60
40
20
0
FLAG
p53

Figure S2

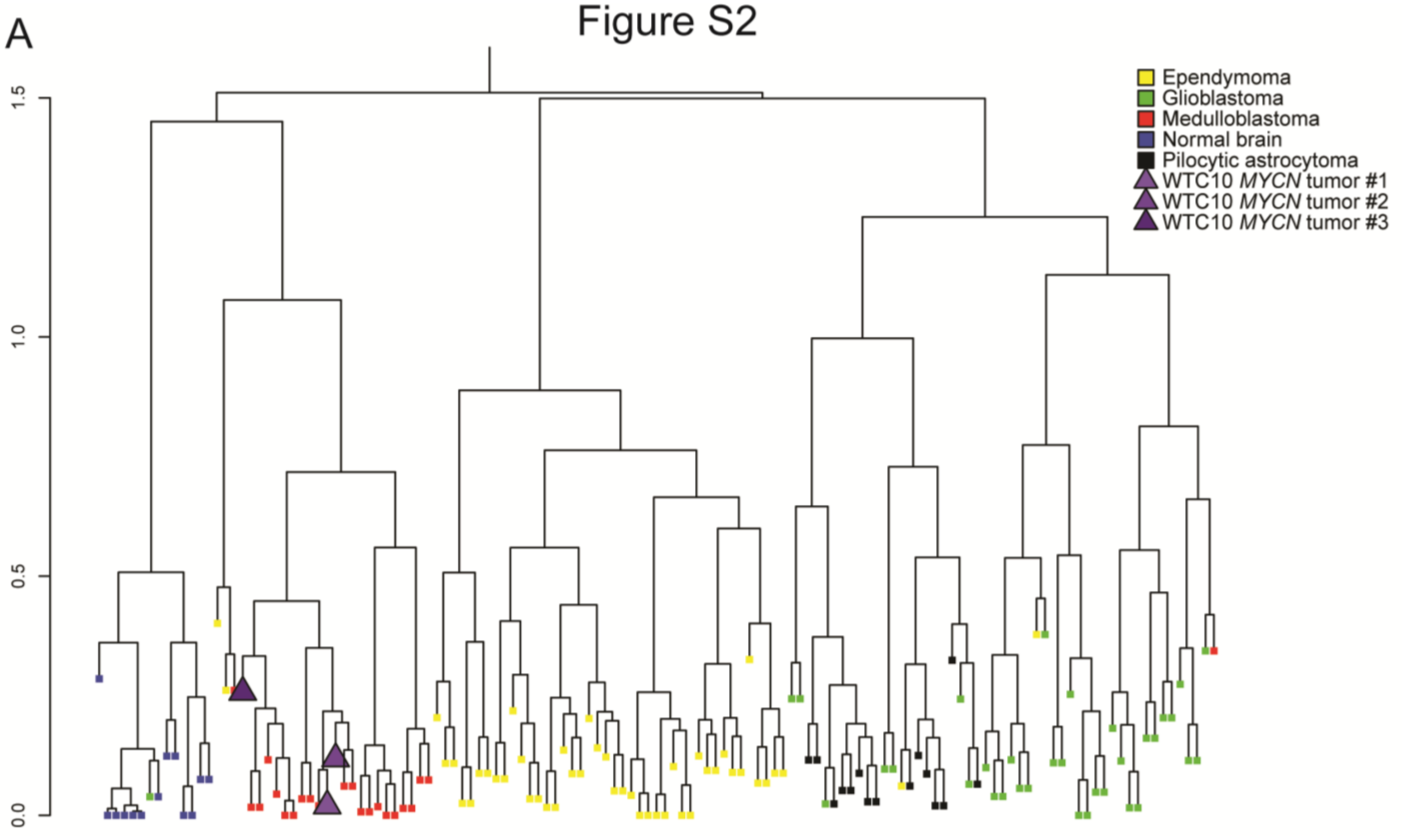
A
1.5
1.0
0.5
0.0
Ependymoma
Glioblastoma
Medulloblastoma
Normal brain
Pilocytic astrocytoma
WTC10 MYCN tumor #1
WTC10 MYCN tumor #2
WTC10 MYCN tumor #3

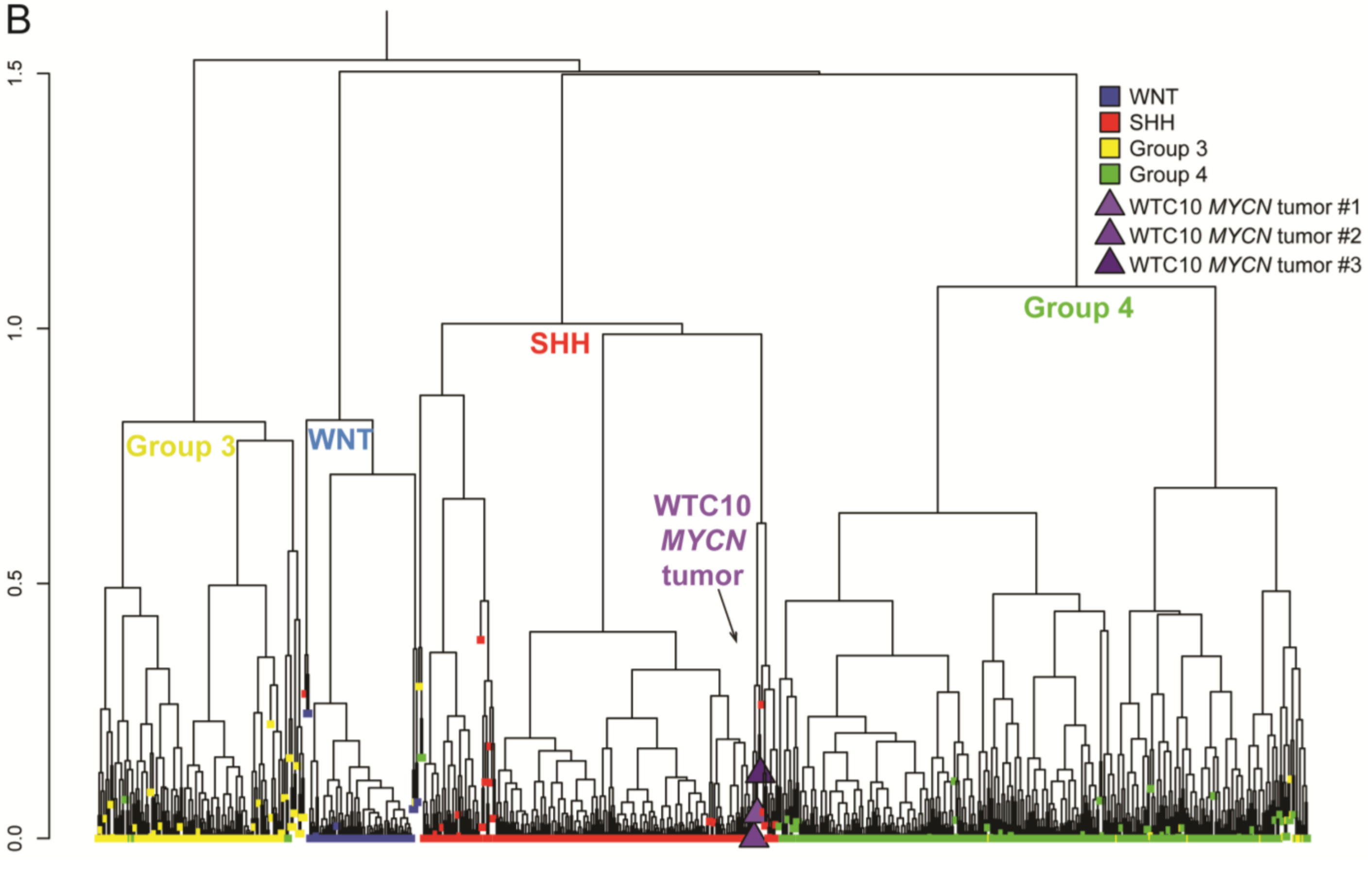
B
1.5
1.0
0.5
0.0
WNT
SHH
Group 3
Group 4
WTC10 MYCN tumor #1
WTC10 MYCN tumor #2
WTC10 MYCN tumor #3
Group 4
SHH
Group 3
WNT
WTC10
MYCN
tumor

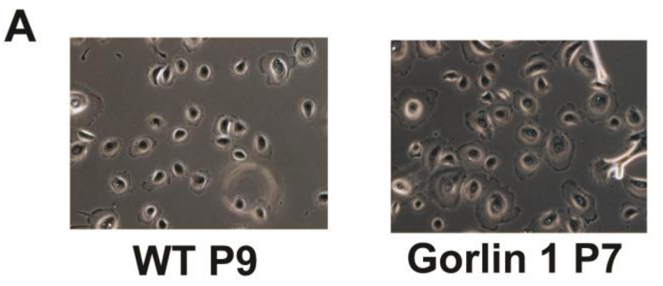

B

WT /Gorlin	PTCH1 mutation (DNA)	PTCH1 mutation (protein)
WT	N/A	N/A
Gorlin 1	1762dupG	V588G_fsX39
Gorlin 2	1925dupC	P643T_fsX11

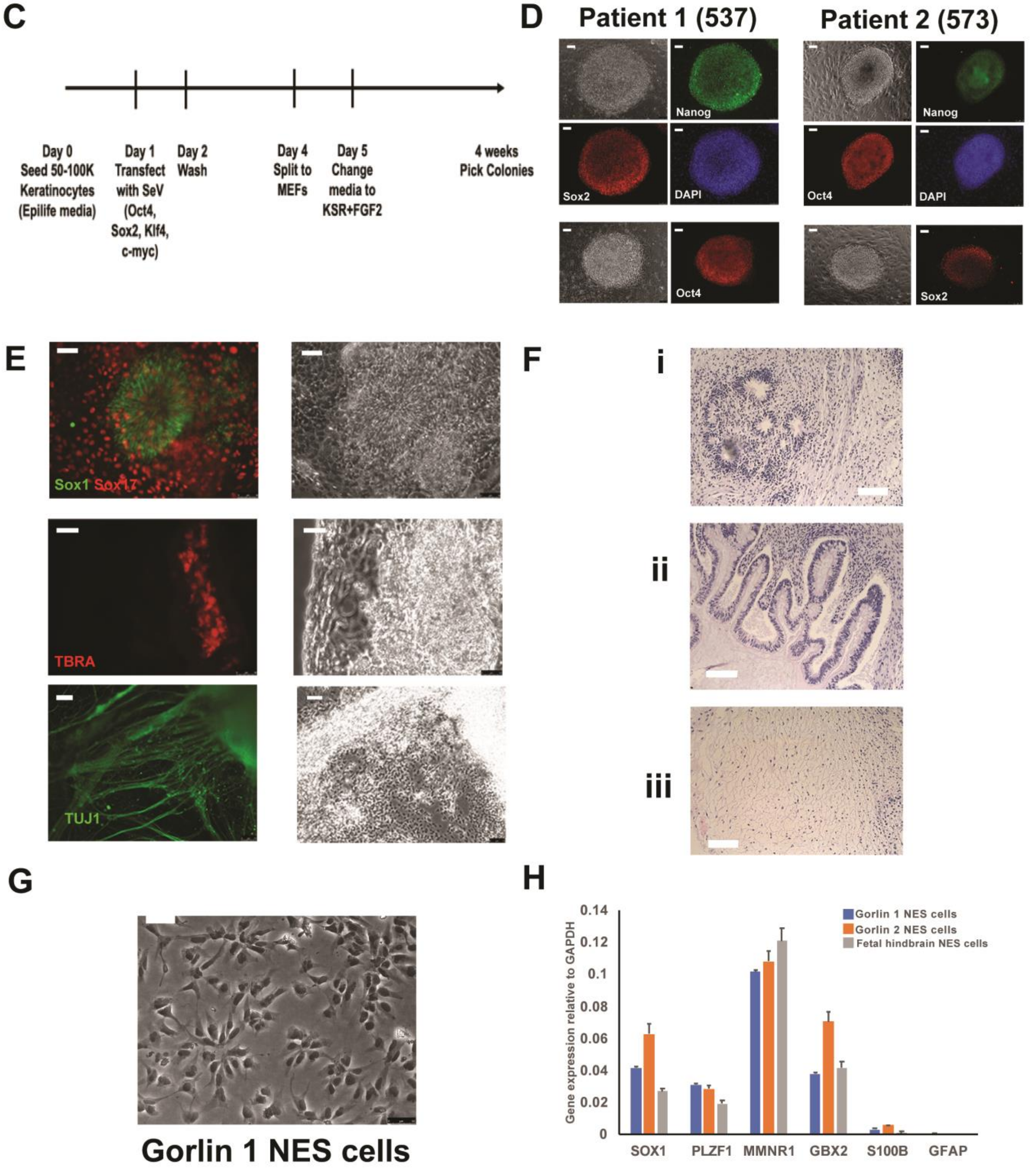

Figure S3

Figure S4

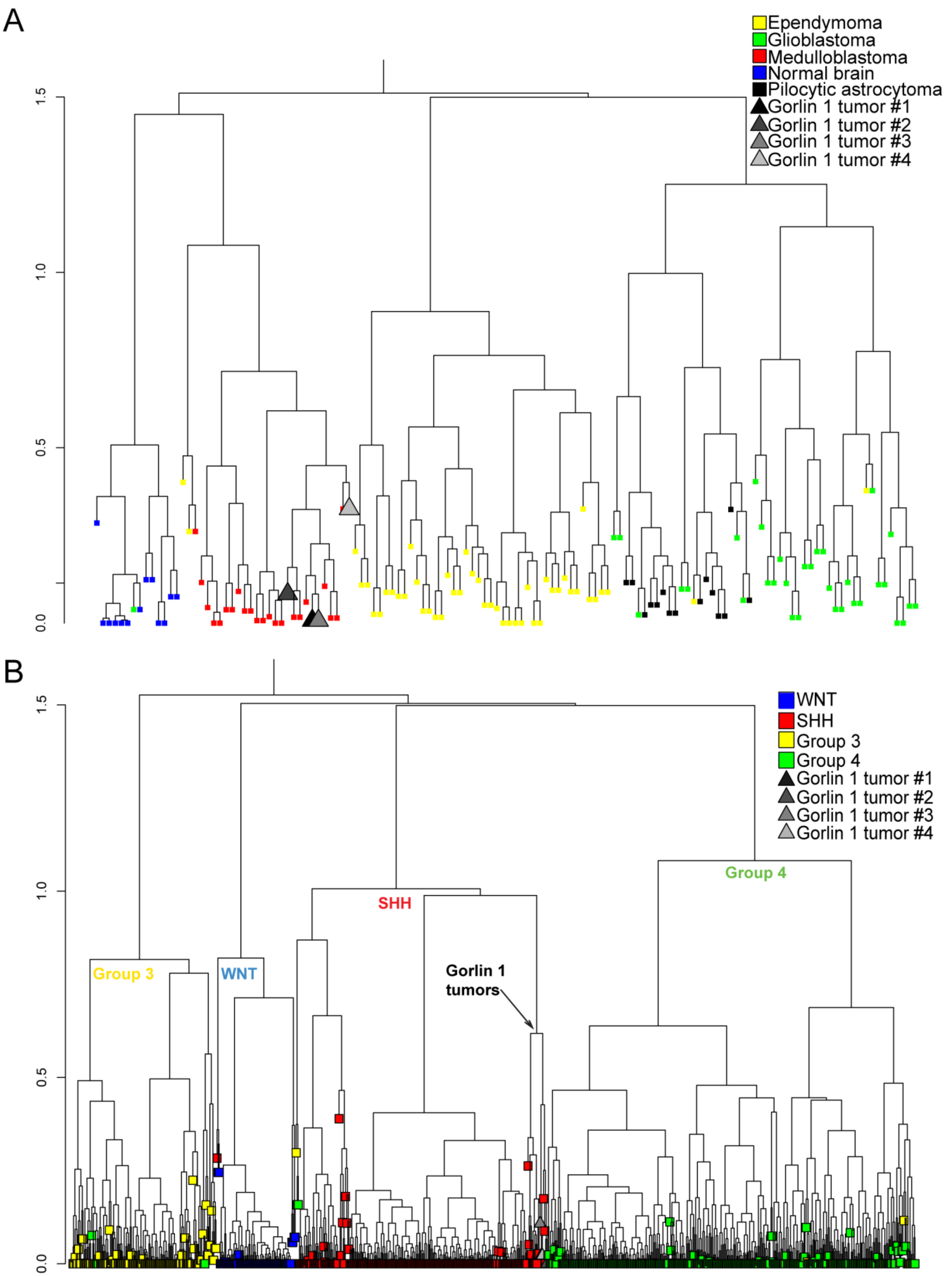
A
Ependymoma
Glioblastoma
Medulloblastoma
Normal brain
Pilocytic astrocytoma
Gorlin 1 tumor #1
Gorlin 1 tumor #2
Gorlin 1 tumor #3
Gorlin 1 tumor #4
0.0
0.5
1.0
1.5
B
WNT
SHH
Group 3
Group 4
Gorlin 1 tumor #1
Gorlin 1 tumor #2
Gorlin 1 tumor #3
Gorlin 1 tumor #4
Group 4
SHH
Group 3
WNT
Gorlin 1 tumors
0.0
0.5
1.0
1.5

Figure S5

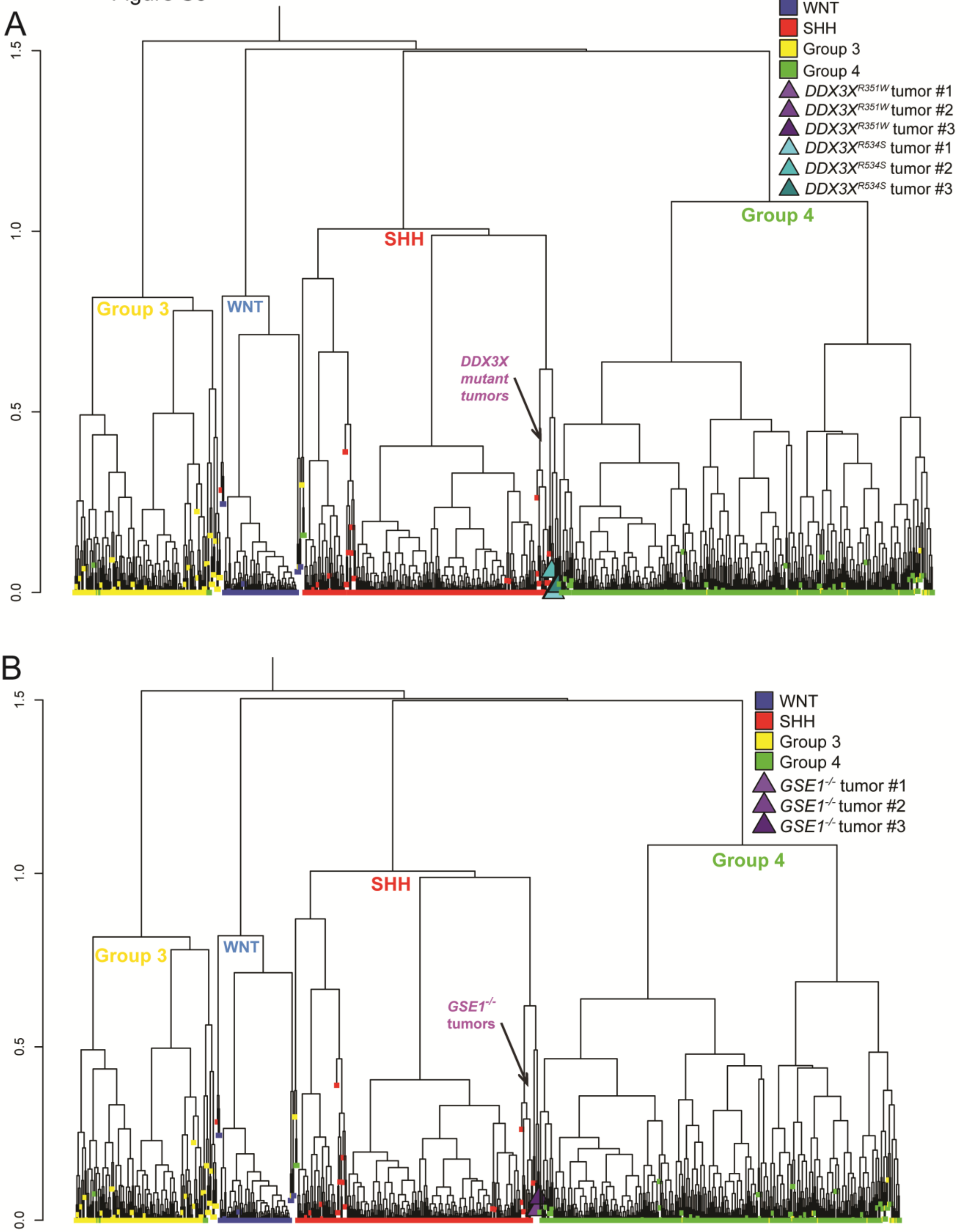
A
1.5
1.0
0.5
0.0
WNT
SHH
Group 3
Group 4
DDX3X R351W tumor #1
DDX3X R351W tumor #2
DDX3X R351W tumor #3
DDX3X R534S tumor #1
DDX3X R534S tumor #2
DDX3X R534S tumor #3
Group 4
SHH
Group 3
WNT
DDX3X mutant tumors
B
1.5
1.0
0.5
0.0
WNT
SHH
Group 3
Group 4
GSE1-/- tumor #1
GSE1-/- tumor #2
GSE1-/- tumor #3
Group 4
SHH
Group 3
WNT
GSE1-/- tumors

Figure S6

A

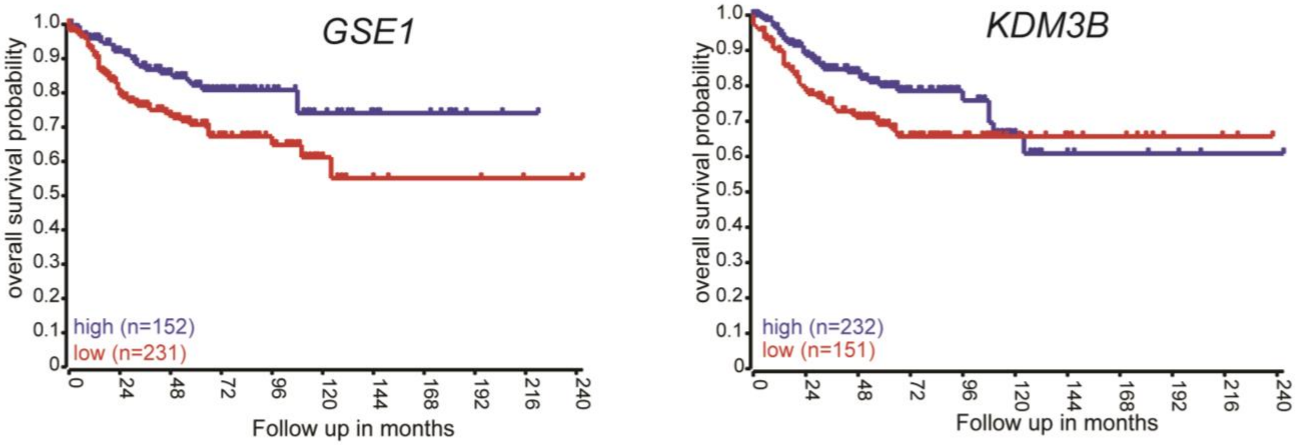

B

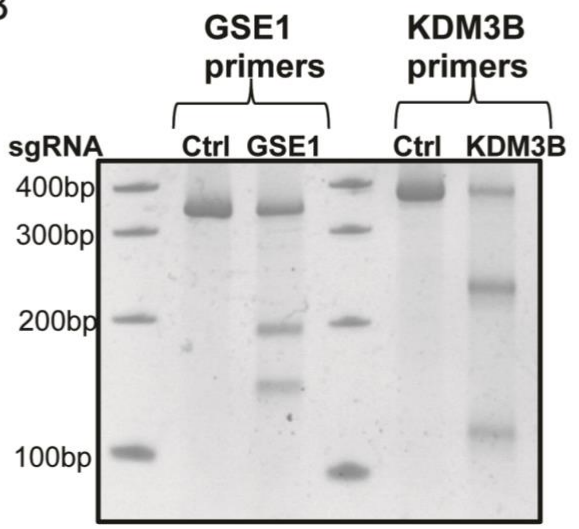

C

Target gene	% mutated allele	% frameshift alleles
GSE1	99.2%	87.9%
KDM3B	96.7%	69.8%

D

GSE1		*KDM3B*
AGCACCCCCCCCGTGGTGACCATCGCTCCAACCA	**REF**	ATACATCCTGGCCAATGTTGGGGACCAGTTCTGCCA
AGCACCCCCCC--------------------------CGCTCCAACCA	**Mut #1**	ATACATCCTGG--------------------------CCAGTTCTGCCA
AGCACCCCCCCCGTG---------CCATCGCTCCAACCA	**Mut #2**	ATACATCCTGGCCAATG----GGGGACCAGTTCTGCCA
AGCACCCCCCC-------------------CCATCGCTCCAACCA	**Mut #3**	ATACATCCTGGCCAATGT--GGGGACCAGTTCTGCCA
AGCACCCCCCCCGTG-----------CATCGCTCCAACCA	**Mut #4**	ATACATCCTGGCCAAT---------GGGACCAGTTCTGCCA
AGCACCCCCCCCG--------------------TCGCTCCAACCA	**Mut #5**	ATACATCCTGGCCAAT-----------GGACCAGTTCTGCCA

E

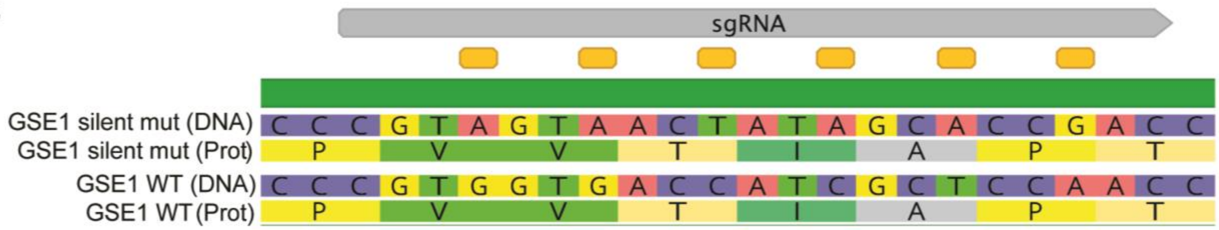

Figure S7

A

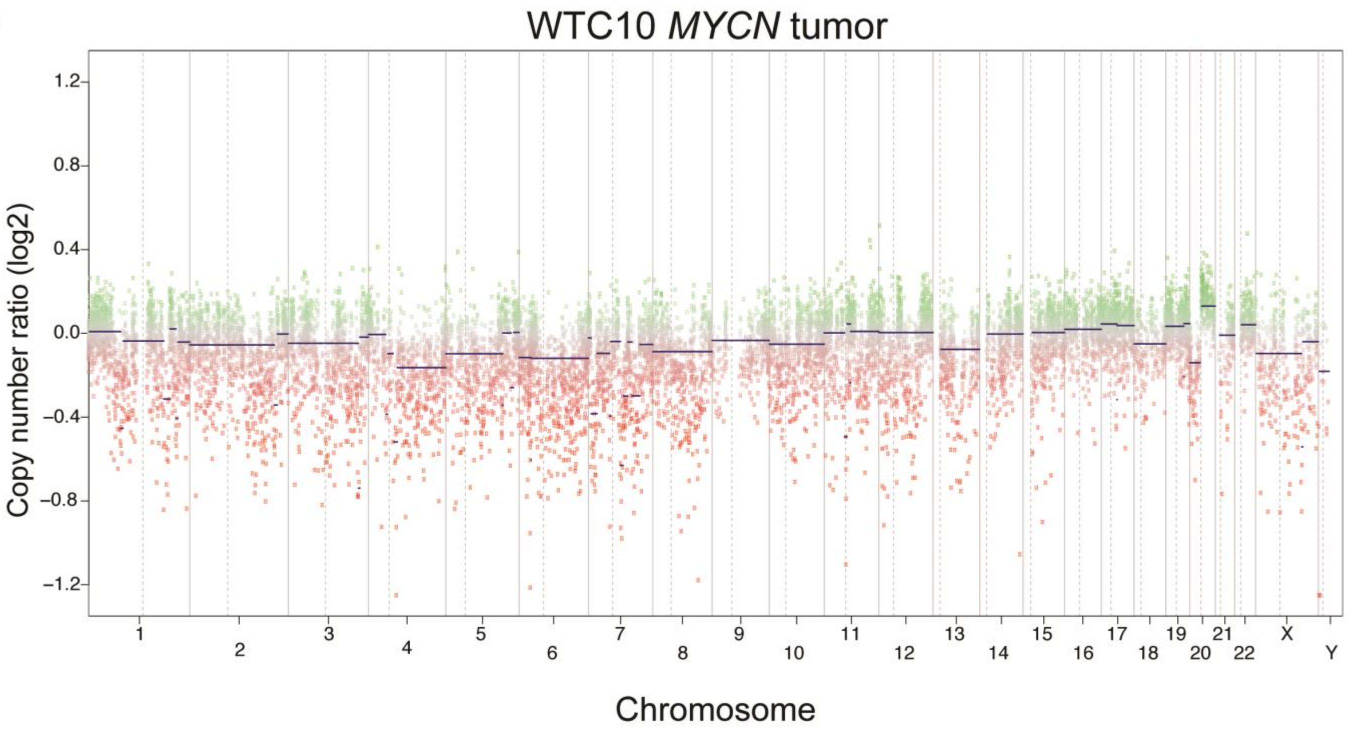
WTC10 *MYCN* tumor
Copy number ratio (log2)
1.2
0.8
0.4
0.0
−0.4
−0.8
−1.2
1 2 3 4 5 6 7 8 9 10 11 12 13 14 15 16 17 18 19 20 21 22 X Y
Chromosome

B

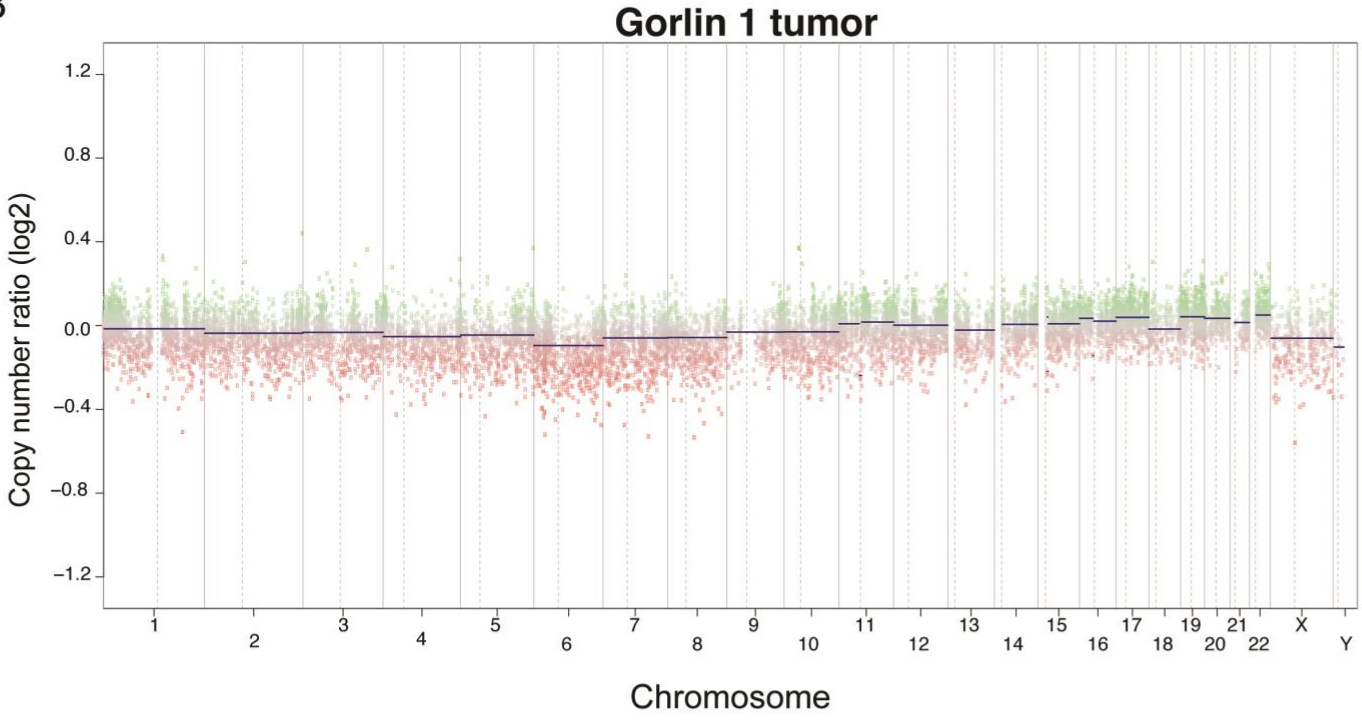
Gorlin 1 tumor
Copy number ratio (log2)
1.2
0.8
0.4
0.0
−0.4
−0.8
−1.2
1 2 3 4 5 6 7 8 9 10 11 12 13 14 15 16 17 18 19 20 21 22 X Y
Chromosome
