# Supplementary material for: Engineering Genetic Predisposition in Human Neuroepithelial Stem Cells Recapitulates Medulloblastoma Tumorigenesis
Source: Cell Stem Cell. 2019 Sep 5;25(3):433–446.e7. doi: 10.1016/j.stem.2019.05.013 (PMC6731167; doi:10.1016/j.stem.2019.05.013)
Supplement: Document S2. Article plus Supplemental Information [file mmc9.pdf]

Resource
Cell Stem Cell

# Engineering Genetic Predisposition in Human Neuroepithelial Stem Cells Recapitulates Medulloblastoma Tumorigenesis

## Graphical Abstract

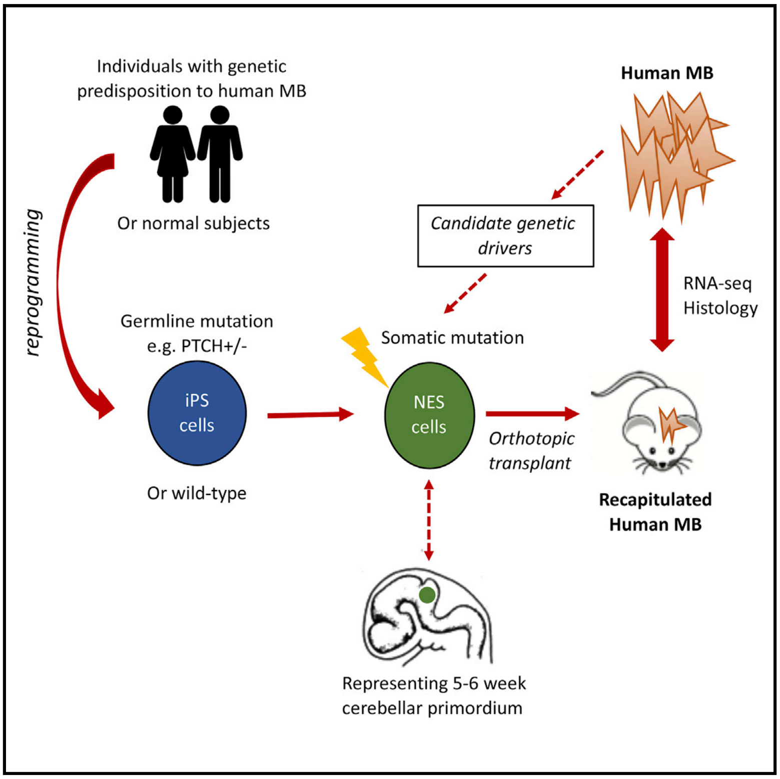


## Highlights

- *MYCN* drives SHH medulloblastoma tumorigenesis in human iPSC-derived NES cells
- NES cells from Gorlin syndrome (*PTCH1*$^{+/-}$) iPSCs generate SHH medulloblastoma
- Mutation of *DDX3X* or *GSE1* accelerates tumorigenesis in Gorlin NES cells

## Authors

Miller Huang, Jignesh Tailor, Qiqi Zhen, ..., Michael D. Taylor, Austin Smith, William A. Weiss

## Correspondence

waweiss@gmail.com

## In Brief

Huang, Tailor, et al. show that neuroepithelial stem (NES) cells derived from normal induced pluripotent stem cells (iPSCs) provide a renewable human cell-based resource to evaluate genetic mutations in medulloblastoma. Misexpression of *MYCN* in both otherwise-normal NES cells and *PTCH1*$^{+/-}$ NES cells derived from patients with Gorlin syndrome each generated medulloblastoma in mice.

Huang et al., 2019, Cell Stem Cell *25*, 433–446
September 5, 2019 © 2019 The Authors. Published by Elsevier Inc.
https://doi.org/10.1016/j.stem.2019.05.013

CellPress

# Engineering Genetic Predisposition in Human Neuroepithelial Stem Cells Recapitulates Medulloblastoma Tumorigenesis

Miller Huang,[1,30] Jignesh Tailor,[2,3,4,5,30] Qiqi Zhen,[1] Aaron H. Gillmor,[6,7,8] Matthew L. Miller,[1] Holger Weishaupt,[9] Justin Chen,[10] Tina Zheng,[1] Emily K. Nash,[1] Lauren K. McHenry,[1] Zhenyi An,[1] Fubaiyang Ye,[1] Yasuhiro Takashima,[2] James Clarke,[2] Harold Ayetey,[2] Florence M.G. Cavalli,[4,11] Betty Luu,[4,11] Branden S. Moriarity,[12,13,14] Shirin Ilkhanizadeh,[1] Lukas Chavez,[15,16] Chunying Yu,[4] Kathreena M. Kurian,[17] Thierry Magnaldo,[18] Nicolas Sevenet,[19] Philipp Koch,[20,21]

(Author list continued on next page)

[1]Department of Neurology and the Helen Diller Family Comprehensive Cancer Center, University of California, San Francisco, San Francisco, CA 94158, USA
[2]Wellcome Trust-MRC Stem Cell Institute, University of Cambridge, Tennis Court Road, Cambridge CB2 1QR, UK
[3]Institute of Cancer Research, Sutton, London SM2 5NG, UK
[4]Developmental & Stem Cell Biology Program, The Hospital for Sick Children, Toronto, ON, Canada
[5]Division of Neurosurgery, The Hospital for Sick Children, Toronto, ON, Canada
[6]Department of Biochemistry and Molecular Biology, University of Calgary, Calgary, AB, Canada
[7]Charbonneau Cancer Institute, University of Calgary, Calgary, AB, Canada
[8]Alberta Children's Hospital Research Institute, Calgary, AB, Canada
[9]Department of Immunology, Genetics and Pathology, Science for Life Laboratory, Uppsala University, 751 85 Uppsala, Sweden
[10]Department of Genetics, Stanford University School of Medicine, Stanford, CA 94305, USA
[11]The Arthur and Sonia Labatt Brain Tumour Research Centre, The Hospital for Sick Children, Toronto, ON, Canada
[12]Department of Pediatrics, University of Minnesota, Minneapolis, MN 55455, USA
[13]Center for Genome Engineering, University of Minnesota, Minneapolis, MN 55455, USA
[14]Masonic Cancer Center, University of Minnesota, Minneapolis, MN 55455, USA
[15]Hopp-Children's Cancer Center (KiTZ), Heidelberg, Germany
[16]Division of Pediatric Neurooncology, German Cancer Research Center (DKFZ), German Cancer Consortium (DKTK), Heidelberg, Germany
[17]Institute of Clinical Neurosciences, Level 1, Learning and Research Building, Southmead Hospital, University of Bristol, Bristol BS10 5NB, UK
[18]Institute for Research on Cancer and Aging, Nice UMR CNRS 7284 INSERM U1081 UNS/UCA, Nice, France
[19]Institut Bergonie & INSERM U1218, Universite de Bordeaux, 229 cours de l'Argonne, 33076 Bordeaux Cedex, France
[20]Central Institute of Mental Health, University of Heidelberg/Medical Faculty Mannheim and Hector Institut for Translational Brain Research (HITBR gGmbH), Mannheim, Germany
[21]German Cancer Research Center (DKFZ), Heidelberg, Germany
[22]MRC Centre for Regenerative Medicine and Cancer Research UK Edinburgh Centre, University of Edinburgh, Edinburgh, UK
[23]Division of Pediatric Neurology, Department of Pediatrics, Oregon Health & Science University, Portland, OR, USA
[24]Papé Family Pediatric Research Institute, Department of Pediatrics, Oregon Health & Science University, Portland, OR, USA

(Affiliations continued on next page)

## SUMMARY

Human neural stem cell cultures provide progenitor cells that are potential cells of origin for brain cancers. However, the extent to which genetic predisposition to tumor formation can be faithfully captured in stem cell lines is uncertain. Here, we evaluated neuroepithelial stem (NES) cells, representative of cerebellar progenitors. We transduced NES cells with *MYCN*, observing medulloblastoma upon orthotopic implantation in mice. Significantly, transcriptomes and patterns of DNA methylation from xenograft tumors were globally more representative of human medulloblastoma compared to a MYCN-driven genetically engineered mouse model. Orthotopic transplantation of NES cells generated from Gorlin syndrome patients, who are predisposed to medulloblastoma due to germline-mutated *PTCH1*, also generated medulloblastoma. We engineered candidate cooperating mutations in Gorlin NES cells, with mutation of *DDX3X* or loss of *GSE1* both accelerating tumorigenesis. These findings demonstrate that human NES cells provide a potent experimental resource for dissecting genetic causation in medulloblastoma.

## INTRODUCTION

Neural stem cell culture systems could potentially advance our understanding of human brain development and disease (Gage, 2000). The capture of self-renewing neural progenitor cells *in vitro* provides scalable cell populations for biochemical or genetic studies. Importantly, neural stem cells can be genetically manipulated or differentiated in a controlled environment


This is an open access article under the CC BY license (http://creativecommons.org/licenses/by/4.0/).

Steven M. Pollard,[22] Peter Dirks,[4,5,11] Michael P. Snyder,[10] David A. Largaespada,[12,13,14] Yoon Jae Cho,[23,24,25] Joanna J. Phillips,[26] Fredrik J. Swartling,[9] A. Sorana Morrissy,[4,6,7,8,11] Marcel Kool,[15,16] Stefan M. Pfister,[15,16,27] Michael D. Taylor,[4,5,11,28] Austin Smith,[2] and William A. Weiss[1,29,31,*]

[25]Knight Cancer Institute, Oregon Health & Science University, Portland, OR, USA
[26]Departments of Neurological Surgery and Pathology, University of California, San Francisco, CA 94158, USA
[27]Department of Pediatric Hematology and Oncology, Heidelberg University Hospital, Heidelberg, Germany
[28]Department of Laboratory Medicine and Pathobiology, University of Toronto, Toronto, ON, Canada
[29]Departments of Pediatrics, Neurosurgery and Brain Tumor Research Center, University of California, San Francisco, San Francisco, CA 94158, USA
[30]These authors contributed equally
[31]Lead Contact
*Correspondence: waweiss@gmail.com
https://doi.org/10.1016/j.stem.2019.05.013

and therefore allow functional studies that would not be possible in human brain.

It has been postulated that brain tumors could develop from neural progenitors that deviate from their developmental pathway (Reya et al., 2001). *Ex vivo* culture of cell populations that are susceptible to tumorigenesis may provide insight into how neural progenitors become malignant (Koso et al., 2012; Pollard et al., 2009). A specific subpopulation of long-term neuroepithelial stem (NES) cells can be captured from human pluripotent stem-cell-derived neural rosettes and propagated long-term in culture (Falk et al., 2012; Koch et al., 2009). These cells maintain neuroepithelial properties in culture; the expression of rosette-stage-specific markers such as *SOX1*, *PLZF1*, *DACH1*, and *MMNR1*; and high neurogenic potency. They exhibit hindbrain regional identity, including expression of *GBX2* and *KROX20*, and maintain responsiveness to ventral and dorsal cell fate cues in a similar way to the developing neuroepithelium (Koch et al., 2009). Furthermore, stem cells expanded directly from the rostral hindbrain neuroepithelium of 5- to 6-week human fetuses show characteristics similar to human induced pluripotent stem cell (iPSC)-derived NES cells, suggesting that these cells are indeed representative of neuroepithelial progenitors in the cerebellar primordium (Tailor et al., 2013). NES cells maintain potency for the cerebellar lineage both *in vitro* and following orthotopic transplantation, including differentiation to cerebellar granule neural precursor (GNP) cells (Tailor et al., 2013). Moreover, they are scalable, genetically stable after long-term passages, and amenable to gene editing and drug screening platforms (Danovi et al., 2010; Falk et al., 2012; McLaren et al., 2013). However, the tumorigenic potential of hindbrain NES cells in the context of tumor-predisposing mutations has not yet been explored.

The rostral hindbrain neuroepithelium (rhombomere 1) comprises two major germinal zones that generate cerebellar cells. The ventricular neuroepithelium lies at the roof of the developing fourth ventricle and harbors precursors of GABAergic Purkinje neurons, Golgi and Lugaro interneurons. By contrast, the upper rhombic lip is located at the interface between rhombomere 1 and the roof plate and generates all the glutamatergic cells of the cerebellum, including cerebellar GNP cells (Millen and Gleeson, 2008; Wang and Zoghbi, 2001; Wingate and Hatten, 1999). GNP cells are thought to be precursors of medulloblastoma, a common malignant brain tumor of childhood and young adults (reviewed in Northcott et al., 2019). GNP cells proliferate extensively in the external granule layer (EGL) of the post-natal brain in response to Sonic Hedgehog (SHH) ligand, a major regulator of cerebellar development (Dahmane and Ruiz i Altaba, 1999; Wechsler-Reya and Scott, 1999). SHH signaling occurs following interaction of the SHH ligand with PTCH1 receptor, which de-represses Smoothened (SMO) and activates downstream target genes (Hooper and Scott, 2005). Aberrations in SHH signaling are well described in medulloblastoma. In particular, inactivating mutations in the *PTCH1* gene leading to constitutive activity of SMO are found in ~25% of medulloblastoma (Cavalli et al., 2017; Northcott et al., 2017).

A germline mutation in *PTCH1* is responsible for an autosomal-dominant, tumor-prone condition, Gorlin syndrome (also known as nevoid basal cell carcinoma syndrome) (Hahn et al., 1996; Johnson et al., 1996). Patients with this syndrome develop multiple basal cell carcinomas of the skin and are also predisposed to medulloblastoma. Analogously, ~15% of *Ptch1*$^{+/-}$ transgenic mice also develop medulloblastoma (Goodrich et al., 1997). Pre-neoplastic lesions can be identified in the EGL of over 50% of these mice in early post-natal life (Oliver et al., 2005), suggesting that the GNP cell population is particularly susceptible to the effects of SHH overactivity. Conditional knockout of *Ptch1* in GNP cells led to the formation of medulloblastoma in all mice by 3 months of age, confirming that GNP cells are susceptible to oncogenic transformation in the context of SHH overactivity (Yang et al., 2008). Interestingly, *Ptch1* deletion in precursors of GNP cells located in the ventricular zone of the dorsal hindbrain also initiated medulloblastoma (Li et al., 2013). Similar results have been observed with overexpression of *Smo* in multipotent cerebellar progenitors (Schüller et al., 2008).

We hypothesized that NES cells, as progenitors of the cerebellar primordium with competence for generation of GNP cells, could provide a human model system to study medulloblastoma initiation and development. We tested this idea, first by transducing NES cells with *MYCN* and second by deriving NES cells from patients with Gorlin syndrome, bearing germline mutations in *PTCH1*, and in each case performing orthotopic transplantation in mice. We then explored the opportunity for functional validation of candidate drivers of medulloblastoma that co-occur with *PTCH1* mutations.

## RESULTS

### *MYCN* Drives Transformation of Normal Human iPSC-Derived NES Cells to SHH Medulloblastoma

We first asked whether neuroepithelial stem (NES) cells can be transformed into brain-tumor-initiating cells by a known driver

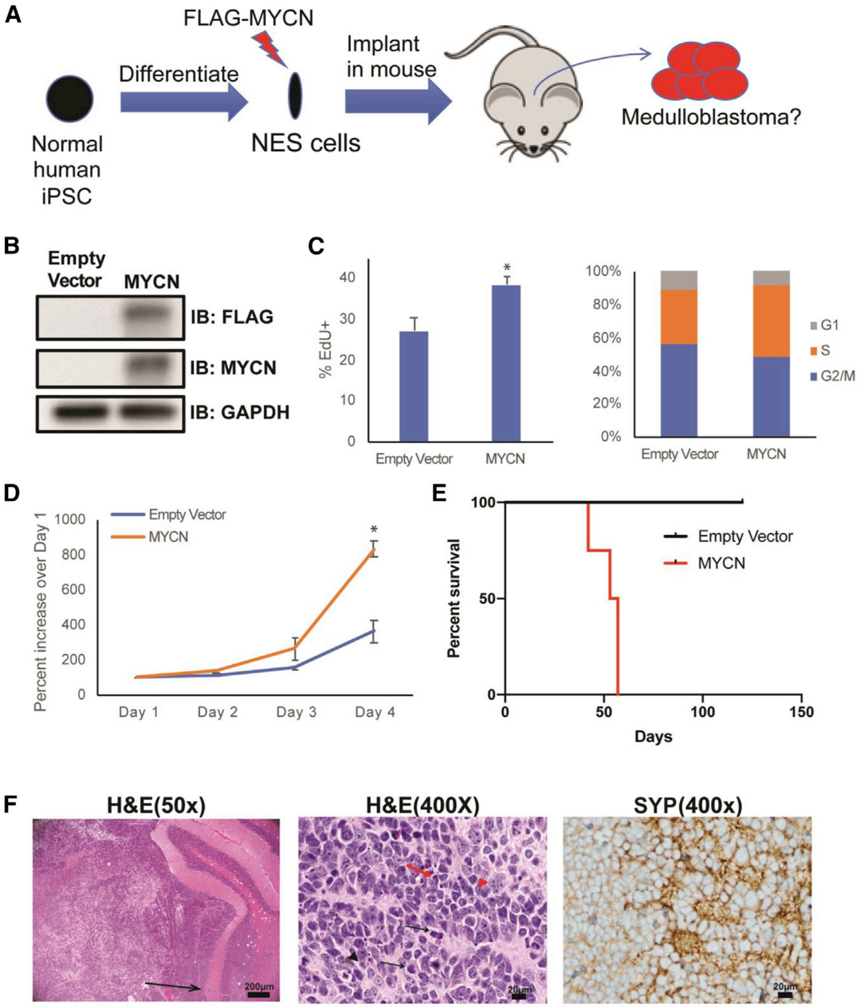


**Figure 1. *MYCN* Drives Transformation of NES Cells to Medulloblastoma**

(A) Schematic showing differentiation of iPSCs to NES cells, transduction with FLAG-MYCN, and orthotopic implantation into mice.

(B) Western blot showing misexpressed MYCN in normal WTC10 NES cells.

(C) Empty vector and *MYCN* NES cells were treated with 5-ethynyl-2′-deoxyuridine (EdU) for 1 h and analyzed via flow cytometry. *MYCN* NES cells show increased EdU incorporation and S-phase fraction. Data are presented as mean ± SEM; $^*p < 0.05$ (t test).

(D) CyQuant Direct cell proliferation analysis showing increased proliferation in *MYCN* NES cells. Data are presented as mean ± SEM; $^*p < 0.05$ (t test).

(E) Kaplan-Meier survival curve of mice injected with empty vector and *MYCN* NES cells (n = 4). p = 0.004 (log-rank test).

(F) (Left) Low magnification (50×) of H&E staining of WTC10 *MYCN* tumors showing implanted *MYCN* NES cells expanding and distorting the mouse cerebellum and invading down Virchow-Robin spaces (arrow). (Middle) High magnification (400×) of H&E staining revealing anaplastic features including frequent mitoses (arrows), cell-cell wrapping (black arrowhead), prominent nuclei (red arrowhead), and karyorrhexis (red arrow) characteristic of large cell and/or anaplastic medulloblastoma. (Right) Immunohistochemical staining for synaptophysin (SYP) highlighting neuroblastic pseudorosettes. Scale bars represent 200 μm for 50× images and 20 μm for 400× images.

See also Figures S1 and S2 and Tables S1, S4, and S6.

of medulloblastoma. Amplification of *MYCN* correlates with high-risk SHH medulloblastoma, and MYCN can drive medulloblastoma in germline and non-germline genetically engineered mouse models (GEMMs) (Swartling et al., 2010; 2012). Human iPSCs derived from keratinocytes of a karyotypically normal adult (WTC10) using episomes (Hayashi et al., 2016) were converted into NES cells as described previously (Falk et al., 2012; Koch et al., 2009) and retained a normal karyotype. Subsequently, NES cells were transduced with FLAG-tagged *MYCN* (Figures 1A and 1B), leading to increased proliferation and a higher proportion of cells in S phase compared to NES cells transduced with empty vector (Figures 1C and 1D). We implanted empty vector control and *MYCN* NES cells orthotopically in immunocompromised mice. *MYCN* NES cells generated tumors between 42 and 57 days post-injection (Figure 1E). Tumors extracted from cerebellum were transplantable, indicating malignancy. Histological analysis revealed an embryonal neoplasm with anaplastic features and immunopositive for synaptophysin, a neuronal marker characteristic of human medulloblastoma, and for FLAG-tagged MYCN (Figures 1F, S1A, and S1C). Although human pluripotent stem cells have been described to spontaneously acquire dominant-negative p53 mutations leading to basal expression of p53 (Merkle et al., 2017), p53 was undetectable by immunohistochemical analysis (Figure S1B and S1C).

Next, we compared molecular characteristics of tumors derived from *MYCN* NES cells (referred as WTC10 *MYCN* tumors) with human medulloblastoma patient tumors. GEMMs have been previously reported to lack DNA methylation changes found in human medulloblastoma (Diede et al., 2013). Using Illumina methylation arrays, we identified 66 differentially methylated regions (DMRs) comparing human patient SHH medulloblastoma (amplified for *MYCN*) to normal cerebellum, whereas 130 DMRs were identified in WTC10 *MYCN* tumors (data not shown). The majority of these DMRs were hypermethylated in both sets of tumors. WTC10 *MYCN* tumors showed similar focal changes in methylation in regions that overlapped 41% (27/66) of the DMRs found in medulloblastoma tumors from patients (Figure 2A).

We then performed RNA sequencing (RNA-seq) and differential gene expression analysis of WTC10 *MYCN* tumors and parental NES cells. Among the 10 most upregulated genes in WTC10 *MYCN* tumors compared to NES cells (Table S1), genes previously linked to cancer progression and malignancy include *AVP*, *BARHL1*, *HELT*, and *LMX1A* (Pöschl et al., 2011; Sun et al., 2007, 2017; Tsai et al., 2013). Global transcriptome analysis also indicated that WTC10 *MYCN* tumors resembled medulloblastoma

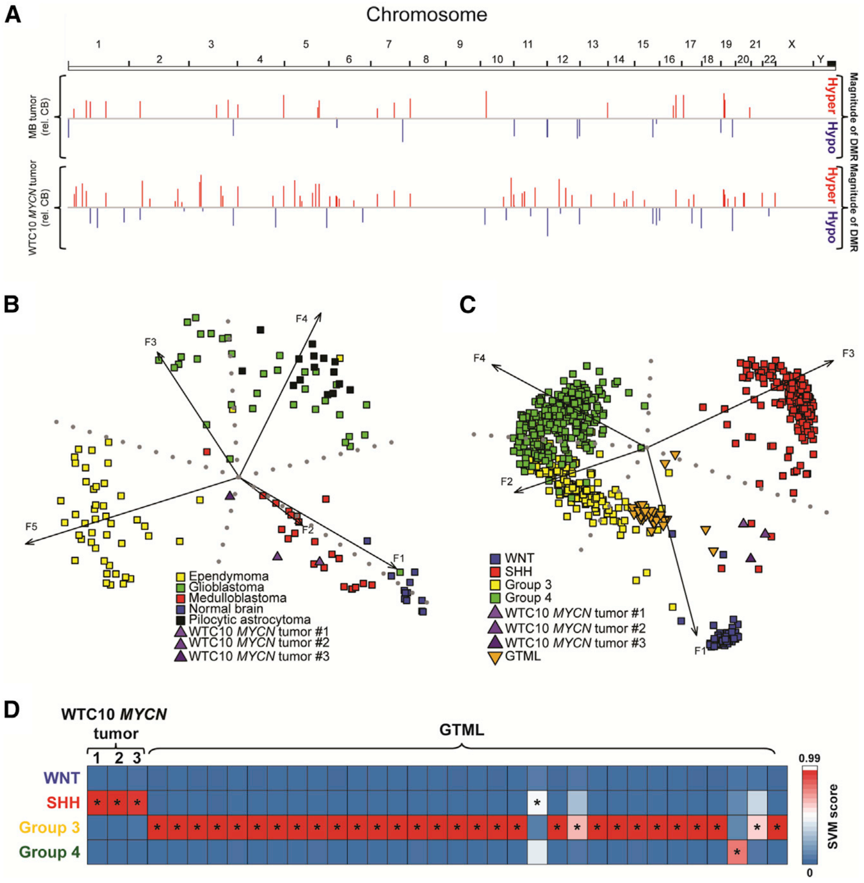


**Figure 2. WTC10 *MYCN* Tumors Align with SHH Medulloblastoma**

(A) Genomic DNA was extracted from WTC10 *MYCN* tumors and analyzed via Illumina methylation arrays. Differentially methylated regions (DMRs) relative to normal human cerebellum were identified in SHH medulloblastoma (MB) from patients with *MYCN*-amplified tumors (top) and compared with WTC10 *MYCN* tumors (bottom). The complexity of overall methylation differences (comparing normal cerebellum with each tumor type) was similar in WTC10 *MYCN* tumors and human medulloblastoma tumors, contrasting a published report comparing GEMM models (including MYCN-driven GEMMs) with human tumors (Diede et al., 2013).

(B) Comparing transcriptomes of WTC10 *MYCN* tumors with other pediatric brain tumors using principal component analysis (PCA) showed WTC10 *MYCN* tumors aligned best with medulloblastoma.

(C and D) Comparison of transcriptomes of human WTC10 *MYCN* tumors and murine MYCN-driven GEMM tumors (GTML, Swartling et al., 2010) with four major subgroups of medulloblastoma using (C) PCA and (D) support vector machine (SVM) classification. WTC10 *MYCN* tumors aligned with SHH subgroup, whereas GTML aligned with group 3 medulloblastoma. For SVM, colors indicate class prediction probabilities (blue, low; red, high), and asterisks denote the predicted class.

See also Figures S2 and S7 and Tables S1, S4, and S6.

more closely than normal brain and other pediatric brain tumors (glioblastoma, pilocytic astrocytoma, and ependymoma; Figures 2B and S2A).

Previously, we generated a GEMM of medulloblastoma driven by misexpression of *MYCN* (referred as GTML) (Swartling et al., 2010). The transcriptome of GTML GEMM tumors aligned with group 3 medulloblastoma, a subgroup in which patient tumors commonly amplify *CMYC* and only rarely amplify *MYCN* (Northcott et al., 2017). In contrast, WTC10 *MYCN* tumors clustered with SHH medulloblastoma (Figures 2C, 2D, and S2B). In support of WTC10 *MYCN* tumors representing SHH medulloblastoma, WTC10 *MYCN* tumors, compared to parental NES cells, showed increased expression of *ATOH1*, a marker of GNP cells, a cell of origin for SHH medulloblastoma (Table S1). Thus, the human stem cell-based model of medulloblastoma showed greater similarity to the relevant primary tumor, as compared to a tumor created in mouse cells using the same oncogenic driver.

## Human iPSCs from Patients with Gorlin Syndrome

Next, we explored if NES cells with mutant *PTCH1* would generate medulloblastoma. Mutation of *PTCH1* occurs frequently in SHH medulloblastoma (Cavalli et al., 2017; Kool et al., 2014; Northcott et al., 2017), and *Ptch1*$^{+/-}$ drives medulloblastoma in mice (Goodrich et al., 1997). To determine whether *PTCH1* loss also generates medulloblastoma in a human stem cell system, we isolated keratinocytes from a healthy control (KTM1, referred as control) and from two different patients with Gorlin syndrome (KAS537 and KAS573 referred as Gorlin 1 and Gorlin 2, respectively; Figures S3A and S3B), both of whom were predisposed to medulloblastoma due to heterozygous germline mutations in *PTCH1* (Cowan et al., 1997; Hahn et al., 1996; Johnson et al., 1996; Wu et al., 2017). Keratinocytes from both Gorlin patients had distinct nucleotide insertions within an exon of one *PTCH1* allele (1762dupG for Gorlin 1 and 1925dupC for Gorlin 2; Figure S3B), resulting in a frameshift and premature STOP codon (V588G_fsX39 for Gorlin 1, P643T_fsX11 for Gorlin 2, Figure S3B). We used Sendai virus to generate iPSCs, confirmed by expression of pluripotent markers NANOG, OCT4, and SOX2 (Figures S3C and S3D). The Sendai virus antigen could not be detected by immunofluorescence in the clonal iPSC lines after five passages (data not shown). When differentiated to embryoid bodies, both Gorlin iPSC lines showed expression of markers for neuroectoderm (SOX1 and TUJ1), mesoderm (TBRA), and endoderm (SOX17) (Figure S3E). We next implanted Gorlin iPSCs into the kidney capsule of immunocompromised mice and obtained teratomas expressing markers of all three germ layers (Figure S3F). These results validated that Gorlin iPSC showed pluripotency.

## Gorlin NES Cells Display Neural Characteristics with Enhanced Proliferation and SHH Signaling

We generated NES cells from Gorlin iPSC (Figure S3G). Similar to control NES cells, both Gorlin NES cells expressed neural markers (NESTIN, SOX2, SOX1, and PAX6) while suppressing pluripotency markers (OCT4 and NANOG; Figure 3A). Importantly, NES cells

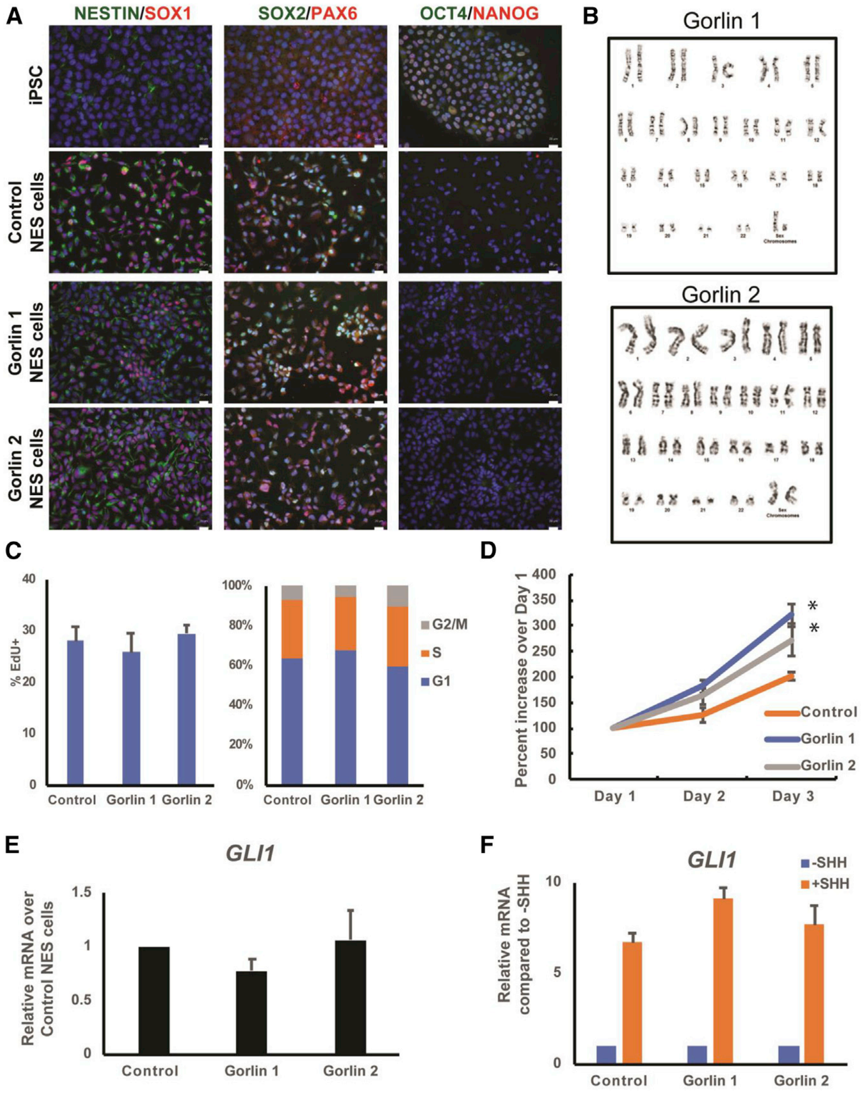


**Figure 3. Gorlin NES Cells Display Neural Characteristics with Enhanced Proliferation and SHH Signaling**

(A) Similar to Control NES cells, Gorlin NES cells expressed NES cell-related markers (NESTIN, SOX1, SOX2, and PAX6), but not pluripotency markers (OCT4 and NANOG). Scale bars represent 20 μm.

(B) Normal karyotypes for both Gorlin NES lines scored from 20 spreads each.

(C) Control and Gorlin NES cells were treated with EdU for 1 h and analyzed by flow cytometry. No significant differences were seen in cell-cycle profile of each cell line. Data are presented as mean ± SEM.

(D) CyQuant Direct cell proliferation assay of control and Gorlin NES cells show both Gorlin NES cell lines grew faster than control NES cells. Data are presented as mean ± SEM. *$p < 0.05$ (t test).

(E) RT-qPCR quantitation of *GLI1* mRNA expression in Control and Gorlin NES cells.

(F) RT-qPCR quantitation of *GLI1* mRNA expression in Control and Gorlin NES cells untreated or treated with SHH ligand (800 ng/mL) for 2 days. Gorlin 1 NES cells show modestly increased sensitivity to stimulation with SHH ligand compared with control NES cells. Data are presented as mean ± SEM.

See also Figure S3 and Table S2.

from both Gorlin iPSC lines retained a normal karyotype (Figure 3B). Cell-cycle profiles were not different between normal and Gorlin NES cells (Figure 3C); however, both Gorlin lines showed a proliferative advantage in the CyQuant Direct cell proliferation assay as compared to control (Figure 3D). Gorlin NES cells maintained expression of neuroepithelial stage (*PLZF1* and *MMNR1*) and hindbrain (*GBX2*) markers, similar to NES cells derived from fetal hindbrain (Tailor et al., 2013) (Figure S3H).

Despite heterozygosity for *PTCH1*, Gorlin NES cells did not show a basal increase in abundance of the SHH downstream target *GLI1* (Figure 3E). In response to stimulation with SHH ligand, one of two Gorlin NES cells (Gorlin 1) showed a modest increased abundance of *GLI1* mRNA compared to control NES cells (Figure 3F). These data show that Gorlin NES cells had a modest growth advantage and may be more sensitive to SHH ligand stimulation compared to control NES cells.

## Gorlin NES Cells Generate SHH Medulloblastoma *In Vivo*

We investigated whether Gorlin NES cells could generate tumors *in vivo*. Control NES cells and NES cells from both Gorlin patients were injected orthotopically into hindbrains of immunocompromised mice (Figure 4A). Mice developed signs of tumor between 102 and 206 days for Gorlin 1 and 254 and 360 days for Gorlin 2 NES cells (Figure 4B). Luminescence imaging revealed that both Gorlin 1 and Gorlin 2 NES cells xenografted equally well, suggesting the difference in penetrance and latency was due to the proliferative advantage of Gorlin 1 NES cells (data not shown; Figure 3D). To further investigate differences between Gorlin 1 and Gorlin 2 NES cells, we performed RNA-seq analysis on both Gorlin NES cell lines. Among the 10 most upregulated genes in Gorlin 1 NES cells compared to Gorlin 2 NES cells, the genes linked to tumor growth and malignancy that might contribute to the difference in latency included *CYP24A1*, *FZD10*, and *HIST1H3C* (Decock et al., 2012; Shiratsuchi et al., 2017; Terasaki et al., 2002) (Table S2). Another possible explanation for the increased penetrance from Gorlin 1 NES cells is due to significantly reduced expression of *PTCH2* (>4-fold) in Gorlin 1 NES cells compared to Gorlin 2 NES cells (Table S2). Loss of one or both copies of *Ptch2* has been shown to accelerate tumorigenesis in $Ptch1^{+/-}$ mice (Lee et al., 2006).

H&E staining revealed an embryonal neoplasm with mild to moderate nuclear pleomorphism and frequent mitoses characteristic of medulloblastoma (Marshall et al., 2014) (Figure 4C). Immunohistochemistry showed abundant expression of synaptophysin (Figure 4C). RNA-seq analysis aligned Gorlin 1 tumors with human SHH medulloblastoma as compared to other subtypes of medulloblastoma and other types of pediatric brain

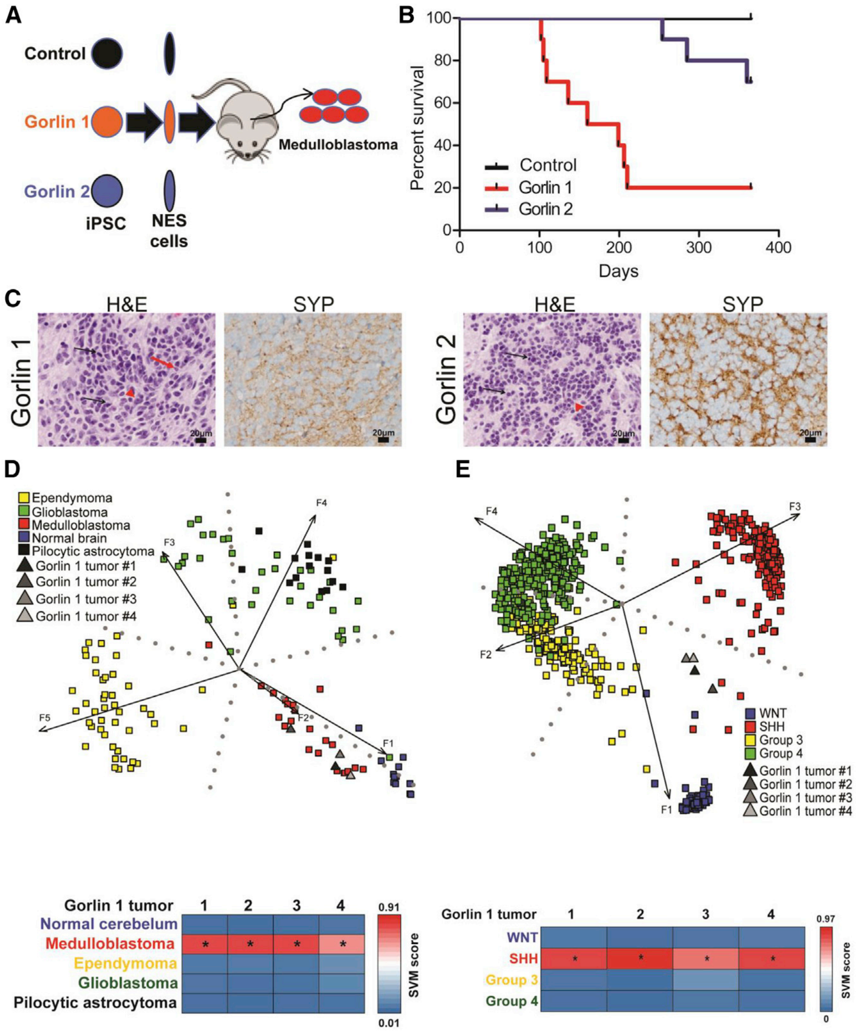


**Figure 4. Gorlin NES Cells Generate SHH Medulloblastoma *In Vivo***

(A) Schematic of differentiation of control and Gorlin iPSC toward NES cells, implantation into hindbrains of immunocompromised mice, and generation of tumors.

(B) Kaplan-Meier survival curve of mice (n = 10) implanted with each cell line. $p < 0.0001$ (log-rank test).

(C) Gorlin tumors showed a hypercellular embryonal neoplasm with indistinct cell borders, frequent mitoses (arrows), prominent nuclei (red arrowhead), karyorrhexis (red arrow), and synaptophysin (SYP) positivity. Scale bars represent 20 μm.

(D) Comparison of transcriptomes of Gorlin 1 tumors with other pediatric brain tumors using (top) PCA and (bottom) SVM classification. Gorlin 1 tumors align with medulloblastoma.

(E) Comparison of transcriptomes of Gorlin 1 tumors with four major subgroups of medulloblastoma using (top) PCA and (bottom) SVM classification. Gorlin 1 tumors aligned with SHH subgroup. For SVM, colors indicate class prediction probabilities (blue, low; red, high), and asterisks denote the predicted class.

See also Figures S4 and S7 and Tables S2, S5, and S7.

tumors (Figures 4D, 4E, S4A, and S4B). Similar to the WTC10 *MYCN* tumors, Gorlin 1 tumors showed elevated expression of the GNP cell marker *ATOH1* compared to the Gorlin 1 NES cells (Table S2). Thus, Gorlin tumors resembled SHH medulloblastoma.

## Human NES Cells Are Not Committed to the GNP Lineage

Since the WTC10 *MYCN* and Gorlin tumors both aligned with SHH medulloblastoma, we sought to determine whether NES cells were committed or "primed" to be in a GNP-like state. We first compared the transcriptomes of WTC10 NES cells, Gorlin 1 NES cells, and Gorlin 2 NES cells with mouse GNPs (Carter et al., 2018). Hierarchical clustering analysis showed the transcriptome profiles of all three NES cells were distinct from GNPs (Figure 5A). Next, for each NES cell line, we either maintained undifferentiated, differentiated spontaneously, or differentiated directly with Wnt3a and GDF7, factors known to stimulate expression of the GNP marker *ATOH1* (Tailor et al., 2013). Upon stimulation of NES cells with Wnt3a and GDF7, analysis by RT-qPCR revealed a substantial increase in expression of *ATOH1* compared to spontaneously differentiated or undifferentiated NES cells (Figure 5B). Thus, while NES cells clearly have the capacity to generate GNPs, they are not committed to that lineage.

## Mutation of *DDX3X* and *GSE1*, but Not *KDM3B*, Accelerates Tumorigenesis in Gorlin 1 NES Cells

We then asked whether the Gorlin NES cell model could be used to test candidate genetic drivers. Sporadic mutation of *PTCH1* occurs mainly in adult medulloblastoma (Cavalli et al., 2017; Kool et al., 2014; Northcott et al., 2017). Among these adult patients, co-occurring mutations are found commonly in candidate genes, including *DDX3X*, an RNA helicase; genetic suppressor element 1 (*GSE1*), a coiled-coiled protein known to interact with HDAC1 (Bantscheff et al., 2011); and lysine demethylase 3B (*KDM3B*), a demethylase with both tumor-suppressive and tumor-promoting effects (Kim et al., 2012; Xu et al., 2018). Whereas *DDX3X* mutations are always missense, mutations in *GSE1* and *KDM3B* are typically frameshifts, nonsense, or deletions (Jones et al., 2012; Kool et al., 2014; Northcott et al., 2012; Pugh et al., 2012; Robinson et al., 2012).

Mutations in *DDX3X* typically occur within its two RNA helicase domains. In SHH medulloblastoma, the most frequent mutations at the N-terminal and C-terminal helicase domains are $DDX3X^{R351W}$ and $DDX3X^{R534S}$, respectively (Kool et al., 2014; Northcott et al., 2017). To evaluate effects of these mutations, we misexpressed FLAG-tagged $DDX3X^{WT}$, $DDX3X^{R351W}$, or $DDX3X^{R534S}$ at similar levels in Gorlin 1 NES cells (Figure 6A). While NES cells with mutant *DDX3X* did not exhibit altered proliferation *in vitro* (Figures 6B and 6C), mutant *DDX3X* accelerated

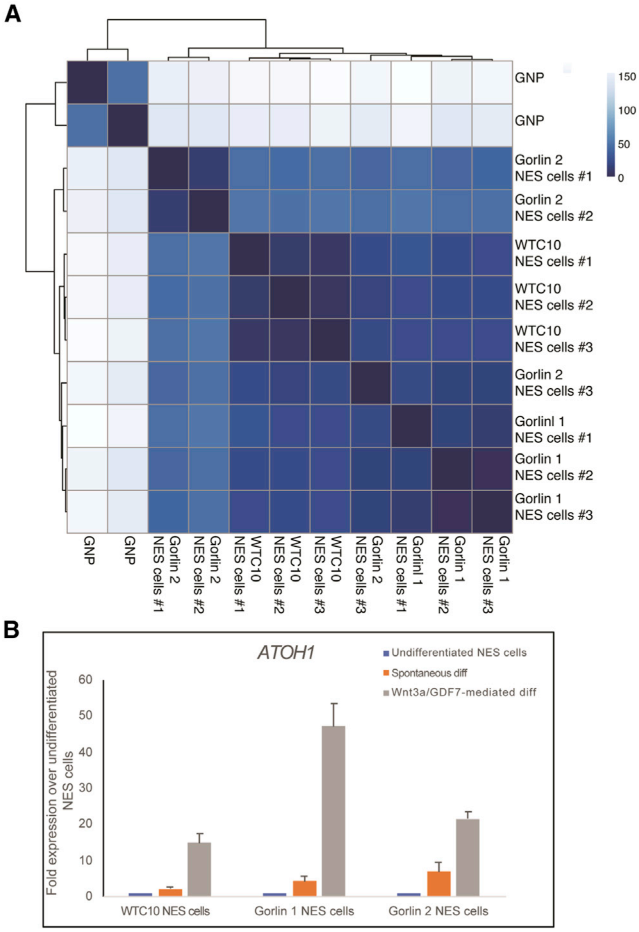


**Figure 5. Human NES Cells Are Not Committed to the GNP Lineage**

(A) Hierarchical clustering heatmap showing human WTC10 NES and both Gorlin NES cells have transcriptome profiles distinct from mouse GNPs (Carter et al., 2018).

(B) WTC10 NES cells and both Gorlin NES cells were differentiated spontaneously or stimulated with Wnt3a (20 ng/mL) and GDF7 (100 ng/mL) for 2 days. Analysis by RT-qPCR shows stimulation with Wnt3a and GDF7 substantially increased GNP marker *ATOH1* compared to spontaneously differentiated cells and parental NES cells. Data are presented as mean ± SEM.

tumorigenesis *in vivo* (Figure 6D). Principal component analysis, support vector machine, and hierarchical clustering show the *DDX3X* mutant tumors aligned with SHH medulloblastoma (Figures 6E, 6F, and S5A). Among the 10 most upregulated genes in Gorlin 1 *DDX3X*$^{R351W}$ tumors (compared to Gorlin 1 tumors), genes previously implicated in tumor growth and malignancy include *HOXA3*, *HOXB3*, *KRT6A*, and *S100A9* (Chen et al., 2013; Inanc et al., 2014; Lim et al., 2016; Zhang et al., 2018) (Table S3). Among the 10 most upregulated genes in Gorlin 1 *DDX3X*$^{R534S}$ tumors (compared to Gorlin 1 tumors), genes previously linked with cancer include *DDX43*, *KRT7*, *HOXB3*, and *NNAT* (Ambrosini et al., 2014; Huang et al., 2016; Lindblad et al., 2015; Siu et al., 2008) (Table S3). These experiments validate *DDX3X* mutations as drivers of SHH medulloblastoma.

Expression of GSE1 and KDM3B correlates inversely with survival in patients with medulloblastoma (Figure S6A). Therefore, we created lentiviral CRISPR/Cas9 plasmids targeting *GSE1* or *KDM3B* to generate indels in each gene (Figure S6B). Transduction of Gorlin 1 NES cells followed by selection with puromycin resulted in cells with frameshift mutations in both genes, as well as decreased levels of GSE1 and KDM3B proteins (Figure 7A). Amplicon sequencing of the target regions (50,000 reads each) revealed mutation frequencies of 96.7% (69.8% for frameshift) for *KDM3B* and 99.2% (87.9% for frameshift) for *GSE1* (Figures S6C and S6D). Neither loss of *GSE1* nor loss of KDM3B significantly affected proliferation in Gorlin 1 NES cells (Figures 7B and 7C). Next, we tested whether either of these mutations influenced tumorigenesis *in vivo*. We injected each Gorlin NES cell line (transduced with Ctrl single guide RNA [sgRNA], GSE1 sgRNA, or KDM3B sgRNA) into hindbrains of immunocompromised mice. Targeting of *GSE1*, but not *KDM3B*, significantly decreased the latency of Gorlin 1 tumors (Figure 7D). RNA-seq analysis of three *GSE1* mutant tumors (referred as *GSE1*$^{-/-}$ tumors) shows that the tumors again clustered with the SHH subgroup (Figures 7E and S5B). Among the 10 most upregulated genes in *GSE1*$^{-/-}$ tumors compared to Gorlin 1 tumors, genes associated with cancer include *CTSG*, *ELANE*, *HIST1H3C*, *NNAT*, *PEG3*, *PRTN3*, and *RNLS* (Alatrash et al., 2017; Decock et al., 2012; Guo et al., 2016; Houghton et al., 2010; Hu et al., 2018; Özata et al., 2017; Siu et al., 2008) (Table S3).

To exclude the possibility that accelerated tumorigenesis could have resulted as an off-target effect, we re-expressed wild-type GSE1 (referred as *GSE1*$^{WT}$) to determine whether shortened latency could be rescued. We first generated cell lines from the *GSE1*$^{-/-}$ tumors. Since these cell lines had stable expression of Cas9 and sgRNA targeting *GSE1*, we generated a plasmid of *GSE1*$^{WT}$ containing silent mutations at the sgRNA binding site (Figure S6E). *GSE1*$^{-/-}$ cells were transduced with the *GSE1*$^{WT}$ construct with silent mutations or with empty vector. Rescue of GSE1 expression was confirmed via western blot (Figure 7F). Empty vector (*GSE1*$^{-/-}$) cells generated tumors between 60 and 63 days post-implantation, whereas re-expressed *GSE1*$^{WT}$ cell lines generated tumors between 67 and 131 days post-transplant, suggesting GSE1 acts as a tumor suppressor (Figure 7G). From these analyses, we conclude that mutant *DDX3X* and loss of *GSE1* act as drivers of SHH medulloblastoma tumorigenesis, while mutations in *KDM3B* may represent a passenger.

## Genomic Analysis of NES Cell-Derived Tumors

We next performed the comparative genomic hybridization array (CGH) and whole exome sequencing (WES; 100× coverage) to identify copy number changes and mutations in WTC10 *MYCN* and Gorlin 1 tumors that may have been acquired *in vitro* or *in vivo*. At the chromosomal level, both sets of tumors lack copy number changes observed by array CGH (Figure S7). At the base pair level, the overall mutation rates were quite low. Each of the three WTC10 *MYCN* tumors had 0 mutations per megabase, and the four Gorlin 1 tumors showed 0.6, 6.7, 0.5, and 16 mutations per megabase (Tables S4 and S5), suggesting the mutant NES cells did not require additional mutations to generate tumors. We then investigated the mutation status of *PTCH1* and *TP53*, as both genes are frequently mutated in SHH medulloblastoma (Cavalli et al., 2017; Kool et al., 2014; Northcott et al., 2017), and dominant-negative mutations of *TP53* are known to be acquired *in vitro* in human pluripotent

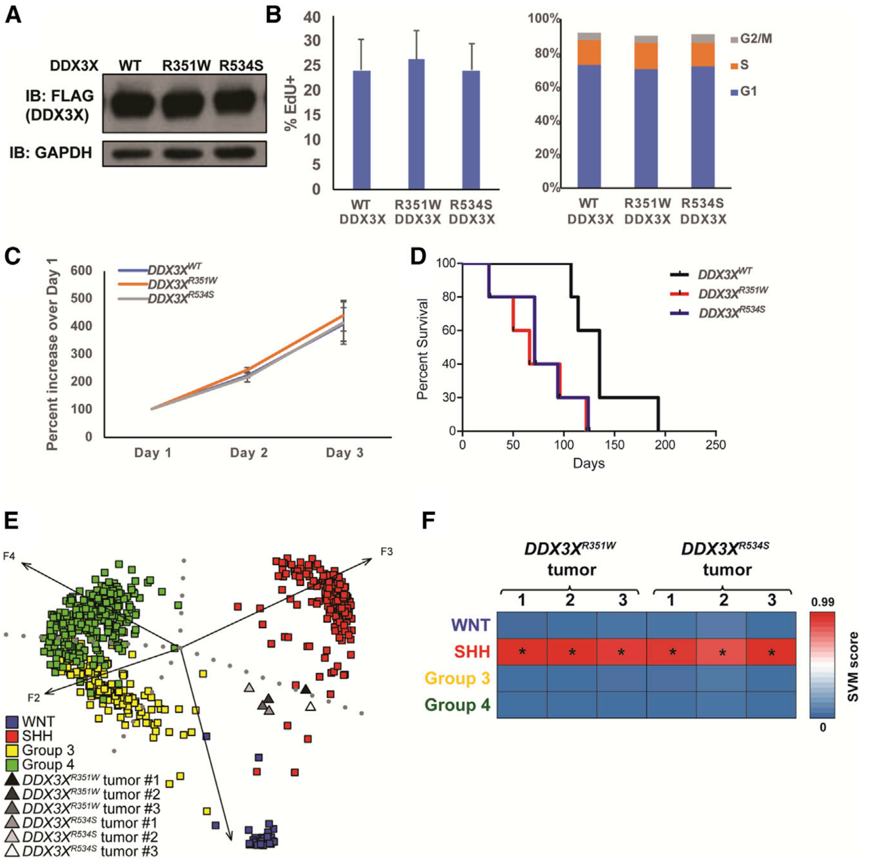


**Figure 6. Mutation of *DDX3X* in Gorlin 1 NES Cells Accelerates Tumorigenesis**

(A) Western blot of FLAG (DDX3X) expression in Gorlin 1 NES cells with $DDX3X^{WT}$, $DDX3X^{R351W}$, or $DDX3X^{R534S}$.

(B) EdU assay of Gorlin 1 NES cells with *DDX3X* mutants. Data are presented as mean ± SEM.

(C) CyQuant Direct cell proliferation assay of Gorlin 1 NES cells with *DDX3X* mutants. Data are presented as mean ± SEM.

(D) Kaplan-Meier survival curve showing accelerated tumorigenesis in mice implanted with NES cells harboring *DDX3X* mutations (n = 5). p = 0.029 (log-rank test).

(E and F) Comparison of transcriptomes of three $DDX3X^{R351W}$ and three $DDX3X^{R534S}$ tumors with the four major subgroups of medulloblastoma using (E) PCA and (F) SVM classification. PCA and SVM classification show all six *DDX3X* mutant tumors subgrouped with SHH medulloblastoma. For SVM, colors indicate class prediction probabilities (blue, low; red, high), and asterisks denote the predicted class.

See also Figure S5 and Table S3.

stem cells (Merkle et al., 2017). Interestingly, we identified a SNP at a coding exon in *TP53* (rs1042522, P72R) in both WTC10 *MYCN* tumors and Gorlin 1 tumors (Tables S4 and S5). This SNP is unlikely to act as a dominant negative, as (1) it is located outside of the DNA-binding domain, where most dominant-negative p53 mutations occur (Petitjean et al., 2007); (2) p53 was not detected by immunohistochemistry (IHC) in WTC10 *MYCN* tumors (Figures S1B and S1C); and (3) control WTC10 NES cells did not generate tumors (Figure 1C). We confirmed rs1042522 was present in the parental WTC10 NES cells by WES and the original iPSC by Sanger sequencing (Table S6; data not shown) and thus was not acquired during differentiation to NES cells or upon implantation in mice. The SNP is not considered damaging according to the International Agency for Research on Cancer TP53 database (http://p53.iarc.fr) and is predicted to be benign by ClinVar (https://www.ncbi.nlm.nih.gov/clinvar/). The minor allele frequency for rs1042522 ranges from 54% to 66% in multiple databases (Table S6).

As expected, WES revealed the insertion mutation at *PTCH1* in the Gorlin 1 tumors leading to a premature STOP codon (Table S7). In addition, a frameshift deletion in *PTCH2* was identified at exon 15, likely explaining the reduced expression levels and at least a contribution to the higher tumor penetrance compared to Gorlin 2 NES cells (Tables S2 and S7).

A previous report by Zindy and collaborators (Zindy et al., 2007) showed that SHH medulloblastoma induced by enforced expression of *N-Myc* in mouse GNP cells sustained loss of *Ptch1*. To address whether human WTC10 *MYCN* tumors similarly showed loss of *PTCH1*, we analyzed WTC10 *MYCN* tumors, identifying a SNP in a coding exon of *PTCH1* (rs357564, P1315L) (Table S4). This SNP was also detected in the original parental WTC10 NES cells by WES (Table S6) and in iPSCs by Sanger sequencing (data not shown). ClinVar lists rs357564 as benign, and minor allele frequencies range from 30%–40% of populations surveyed in similar databases described for the *TP53* SNP. Since both the *PTCH1* SNP and the *TP53* SNP did not produce a tumor in our empty vector WTC10 NES cells (Figure 1C), both occur at high frequencies in the general population, and (for *TP53*) the SNP is located outside the hotspot region for dominant-negative mutations, neither is likely to affect tumorigenesis. Thus, our analysis suggests the mutations we introduced into NES cells represent the major drivers of diseases observed.

## DISCUSSION

Pediatric embryonal cancers may occur when progenitors harbor mutations that cause deviation in their normal developmental program (Northcott et al., 2019). GEMMs have provided support for this model but show clear differences with human tumor phenotypes. Here, we demonstrate that defined genetic perturbations in a specific class of human progenitor cells lead to the formation of a distinct human cancer phenotype.

Medulloblastoma is among the best characterized of all cancers genetically. While the overall 5-year survival rate for medulloblastoma is 80% (Drezner and Packer, 2016), standard of care treatment with intracranial surgery, radiation, and intensive chemotherapy significantly impacts cognition and growth. Tumors can be divided into four main molecular subgroups (SHH, WNT, group 3, and group 4), although further genetic heterogeneity exists within each group (Cavalli et al., 2017;

CellPress

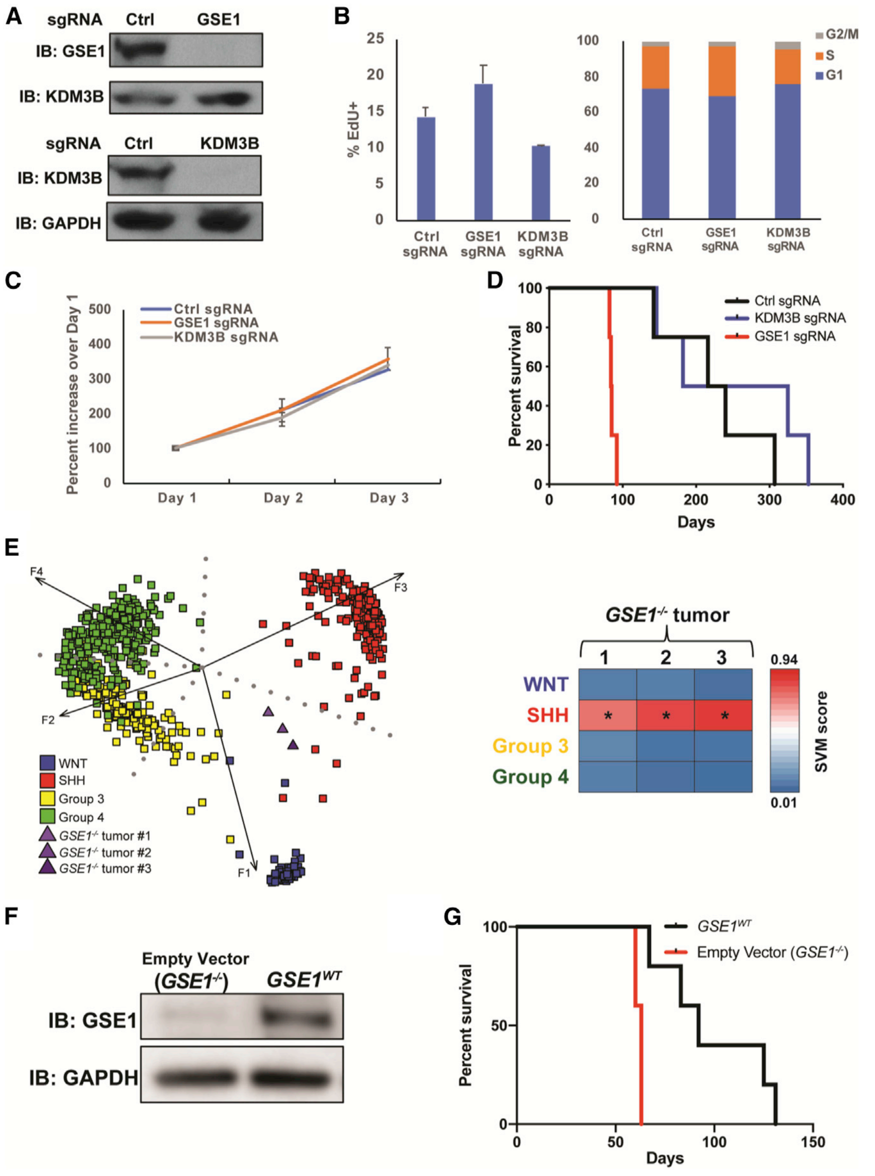


**Figure 7. Knockout of *GSE1*, but not *KDM3B*, in Gorlin 1 NES Cells Accelerates Tumorigenesis**

(A) Western blot showing loss of expression of *GSE1* (top) and KDM3B (bottom) in response to CRISPR/Cas9 knockout.

(B) EdU assay of Gorlin 1 NES cells with control (Ctrl), *GSE1*, or *KDM3B* sgRNA. Data are presented as mean ± SEM.

(C) CyQuant Direct cell proliferation assay of Gorlin 1 NES cells with Ctrl, *GSE1* or *KDM3B* sgRNA. Data are presented as mean ± SEM.

(D) Kaplan-Meier curve showing reduced survival of mice injected with Gorlin 1 NES cells harboring *GSE1* sgRNA compared with Ctrl sgRNA or *KDM3B* sgRNA (n = 5). p = 0.0032 (log-rank test).

(E) PCA (left) and SVM (right) classification showing three $GSE1^{-/-}$ tumors subgrouped with SHH medulloblastoma.

(F and G) $GSE1^{-/-}$ cell lines were transduced with empty vector or the silent mutant *GSE1* ($GSE1^{WT}$). Both cell lines were (F) analyzed by western blot analysis and (G) injected into mice (n = 5). p = 0.0031 (log-rank test).

See also Figures S5 and S6 and Table S3.

Northcott et al., 2017). Germline mutation in *PTCH1* predisposes patients to medulloblastoma, with mutation in *PTCH1* also occurring commonly in sporadic disease. In a recent clinical trial to treat mutant *PTCH1* tumors using the SMO inhibitor vismodegib, tumors showed regression initially but eventually relapsed (Robinson et al., 2015). Moreover, no targeted treatments exist for children with *MYCN*-amplified SHH tumors, a highly lethal subtype. Our findings establish two human stem cell-based genetic models of SHH medulloblastoma that could be used as tools for genetic screening or drug discovery for targeted therapies.

Misexpression of *MYCN* in human NES cells transplanted orthotopically in mice generated medulloblastoma *in vivo*. Amplification of *MYCN* is normally found in SHH or group 4 medulloblastoma and rarely in group 3 tumors (Northcott et al., 2017). Consistent with these associations, transcriptome analysis of human WTC10 *MYCN* tumors from NES cells aligned closest to SHH medulloblastoma. In contrast, the GTML model, our previously characterized GEMM for medulloblastoma also driven by *MYCN*, more closely resembled group 3 medulloblastoma (Figures 2C and 2D), a subgroup of medulloblastoma associated more commonly with amplification of *CMYC*. Furthermore, while GTML tumors showed much fewer regions of hypermethylation than human medulloblastoma patient tumors (Diede et al., 2013), the human WTC10 *MYCN* tumors exhibited DMRs at almost half the sites found in patient-derived tumors (Figure 2A). Thus, the human WTC10 *MYCN* tumor model of medulloblastoma recapitulates the specific tumor subtype and epigenetic profile more accurately than the *MYCN* GEMM.

We then derived iPSCs from patients with Gorlin syndrome and differentiated them to NES cells. We showed that Gorlin NES cells recapitulated medulloblastoma predisposition and generated tumors following orthotropic transplantation. This model was leveraged to test candidate cooperating factors found in SHH tumors with somatic mutations in *PTCH1*. Missense mutation in the RNA helicase *DDX3X* and loss of *GSE1* both cooperated with *PTCH1* heterozygosity to drive tumorigenesis, whereas loss of candidate driver *KDM3B* did not (Figures 6D and 7D). Thus, we have generated two genetically distinct models for medulloblastoma using a human stem cell system, exemplified functional evaluation of candidate cooperating genes in tumor initiation, and describe new experimental resources for the dissection of cooperative events in human medulloblastoma tumorigenesis.

Interestingly, chromosome copy number and single nucleotide variation analysis of tumors derived from human NES cells revealed few additional mutations compared to NES cells grown *in vitro* (Figure S7; Tables S4 and S5). The low mutation rate suggests the specific genetic manipulations we introduced into the NES cells were sufficient to drive tumorigenesis. Although both *MYC* and *PTCH1*$^{+/-}$ usually require additional genetic mutations for transformation, NES cells are a stable and highly proliferative cell population (Koch et al., 2009), and thus, the genes responsible for self-renewal could conceivably sensitize NES cells to transform from only a single genetic event. In contrast, mouse models of MYCN-driven medulloblastoma targeting progenitors of NES cells exhibited copy number alterations at the chromosomal level that likely cooperated with MYCN to generate tumors (Swartling et al., 2010; Zindy et al., 2007). Although a previous study found a population of Nestin-expressing progenitors (NEPs) in mice to be more genomically unstable than GNPs, these NEPs are a quiescent population and less proliferative than GNPs (Li et al., 2013).

Both NES cells and neural stem cells (NSCs) are multipotent and have the capacity to generate neurons, astrocytes, and oligodendrocytes. However, NES cells have a significantly stronger tendency to differentiate toward neurons (Koch et al., 2009). This bias toward neuronal lineages may explain why transformed NES cells (both Gorlin and MYCN driven) resemble medulloblastoma more than other brain tumor types thought to be derived from glial lineages such as glioblastoma, ependymoma, and pilocytic astrocytoma (Figures 2B, S2B, 4D, and S4A). Similarly, the NEPs described by Li et al. are also more likely to differentiate toward neurons, and knockout of *Ptch1* in NEPs generated tumors resembling medulloblastoma (Li et al., 2013). Thus, a human stem cell-based model of a glial-derived brain tumor would likely require a cell type other than NES cells.

In addition to the association between Gorlin syndrome and SHH medulloblastoma, other medulloblastoma subtypes are also known to develop through genetic predisposition. Patients with Turcot's syndrome, who have a germline mutation in the adenomatous polyposis of the colon (*APC*) gene (Hamilton et al., 1995), are predisposed to WNT-subtype medulloblastoma, which has a more indolent clinical course than SHH-subtype medulloblastoma (Northcott et al., 2019). While it will be of interest to model the precursors of WNT-subtype medulloblastoma via iPSCs derived from individuals with Turcot's syndrome, studies in mice suggest the molecular and clinical differences between medulloblastoma subtypes may be due to different developmental cells of origin in distinct regions of the cerebellum (lower rhombic lip for WNT, external germinal layer [EGL] for SHH, ventricular zone or EGL for group 3, and upper rhombic lip or nuclear transitory zone for group 4; Azzarelli et al., 2018; Gibson et al., 2010; Kawauchi et al., 2012; Lin et al., 2016; Pei et al., 2012; Schüller et al., 2008; Yang et al., 2008). Thus, the transcriptome of these various cell types may provide the appropriate environment for specific mutations to transform the cells to medulloblastoma and would explain why some mutations are specific to a particular subgroup.

These divergent subpopulations of hindbrain cells could be generated from iPSCs or NES cells that express gene-specific reporter proteins to further investigate the influence of cellular origin on human medulloblastoma phenotype. Comparing isogenic lines that model distinct cells of origin could address whether mutations occur early at the NES cell stage to direct differentiation down a particular cell lineage or whether mutations occur after NES cells differentiated to the appropriate cell type. Although our study showed the NES cells can generate SHH medulloblastoma, these cells were not primed to generate GNP cells, as the transcriptomes were quite distinct and spontaneous differentiation of NES cells does not lead to maximal expression of the GNP marker *ATOH1* (Figures 5A and 5B). Instead, NES cells are prone to generating neurons of different lineages and have the capacity to generate GNPs.

The modeling of different medulloblastoma subtypes will have important implications for future targeted therapy. Xenograft models of human medulloblastoma in mice could be tested with specific genetic or drug therapies developed through screening of predisposed or transformed stem cells. In addition, the recapitulation of human medulloblastoma *in vitro* and *in vivo* could lead to methods for halting progression of premalignant cells. These strategies may help to prevent the progression of tumors in children and obviate the need for adjuvant chemotherapy or radiotherapy with the associated harmful side effects.

In conclusion, we demonstrate that human NES cells with genetic tumor predisposition generate bona fide medulloblastoma. Using known drivers of disease (*PTCH1* and *MYCN*), we demonstrate robust generation of SHH-subtype medulloblastoma and cooperativity between heterozygosity for *PTCH1* with mutations in *DDX3X* or *GSE1*. Thus, human NES cell-based models offer a powerful system for refined analyses of human medulloblastoma tumorigenesis, with the prospect of future applications in screening candidate therapeutic compounds.

## STAR★METHODS

Detailed methods are provided in the online version of this paper and include the following:


- KEY RESOURCES TABLE
- LEAD CONTACT AND MATERIALS AVAILABILITY
- EXPERIMENTAL MODEL AND SUBJECT DETAILS
  - Animals
  - iPSC culture
- METHOD DETAILS
  - Gorlin iPSC derivation
  - Teratoma formation assay
  - Embryoid body formation and differentiation
  - Differentiation to and maintenance of neuroepithelial stem (NES) cells
  - Spontaneous and direct differentiation of NES cells
  - CRISPR plasmid and mutation detection
  - Karyotype analysis
  - RT-qPCR
  - Cell proliferation assays
  - RNA-seq
  - Differential gene expression analysis of NES cells and tumors derived from NES cells
  - Comparison of transcriptomes of tumors from NES cells with different brain tumor types and medulloblastoma subgroups
  - Microarray expression preprocessing
  - Cross-dataset classifications

- ○ Granular Neural Precursor (GNP) comparison
- ○ Whole Exome Sequencing (WES) and variant discovery
- ○ Copy number variation analysis
- ○ Methylation array analysis
- ● QUANTIFICATION AND STATISTICAL ANALYSIS
  - ○ Mutational rate estimates
  - ○ Quantitation of IHC for p53 and FLAG-MYCN
  - ○ Statistical analysis
- ● DATA AND CODE AVAILABILITY

## SUPPLEMENTAL INFORMATION

Supplemental Information can be found online at https://doi.org/10.1016/j.stem.2019.05.013.

## ACKNOWLEDGMENTS

We would like to thank Noemi Fusaki for the Sendai virus vectors. This study was supported in part by the HDFCCC Laboratory for Cell Analysis Shared Resource Facility through NIH grant P30CA082103. M.H. was supported by a postdoctoral fellowship (PF-13-295-01–TBG) from the American Cancer Society, an Alex's Lemonade Stand Foundation Young Investigator Award, a Family Support Award from the Research Evaluation & Allocation Committee at UCSF, and a K99 Pathway to Independence Award from the NIH NCI (K99CA197484). J.T. was supported by a clinical research fellowship from the National Institute of Health Research (United Kingdom) and a postdoctoral research training fellowship from the Wellcome Trust (United Kingdom). T.M. was supported by the French Government (National Research Agency [ANR]; CNRS; INSERM) through the Investments for the Future LABEX SIGNALIFE (program reference ANR-11-LABX-0028-01), the UNS (Université de Nice Sophia Antipolis) and Association René Tourraine, Fondation ARC (SFI201212055859), the Fondation de l'Avenir, the Société Française de Dermatologie, and the Institut National Du Cancer. P.K. was supported by the Hector Stiftung II gGmbH. Histopathologic processing and analysis were supported in part by the Pediatric Brain Tumor Foundation and UCSF Brain Tumor SPORE (P50 CA097257 to J.J.P.). A.S. is a Medical Research Council Professor and is supported by the Medical Research Council of the United Kingdom (G1001028). W.A.W. and M.D.T. were supported by a grant from the V Foundation (T2017-020) and by R01CA159859 and R01NS106155. W.A.W. was supported by the NIH grants R01NS089868, U01CA217864, and P30CA82103; Brain Tumour Charity grant GN356; the Evelyn and Mattie Anderson Chair; the Ross K. MacNeill Foundation; the Pediatric Brain Tumor Foundation; the V Foundation; and the Samuel G. Waxman Foundation.

## AUTHOR CONTRIBUTIONS

Conceptualization, M.H., J.T., A.S., and W.A.W.; Methodology, M.H., J.T., and P.K.; Writing – Original Draft, M.H., J.T., A.S., and W.A.W.; Writing – Review & Editing, M.H., J.T., A.S., and W.A.W.; Validation: M.H., J.T., Q.Z., A.H.G., H.W., N.S., P.K., A.S.M., and N.S.; Formal Analysis: M.H., J.T., A.H.G., H.W., J.C., F.M.G.C., L.C., J.J.P., and A.S.M.; Investigation: M.H., J.T., Q.Z., M.L.M., T.Z., E.K.N., L.K.M., Z.A., F.Y., Y.T., H.A., B.L., S.I., C.Y., K.M.K., and S.M. Pollard; Resources: Y.T., B.S.M., T.M., N.S., P.K., P.D., M.P.S., D.A.L., Y.J.C., F.J.S., A.S.M., M.K., S.M. Pfister, M.D.T., A.S., and W.A.W.; Visualization: M.H., J.T., A.H.G., H.W., J.C., and J.J.P.; Supervision: F.J.S., A.S.M., M.K., S.M. Pfister, M.D.T., A.S., and W.A.W.; Project Administration: M.H., J.T., J.C., A.S., and W.A.W.; Funding Acquisition, M.H., J.T., P.D., A.S., and W.A.W.

## DECLARATION OF INTERESTS

W.A.W. is a co-founder of StemSynergy Therapeutics.

Received: April 18, 2018
Revised: March 15, 2019
Accepted: May 13, 2019
Published: June 13, 2019

## REFERENCES

1000 Genomes Project Consortium, Auton, A., Brooks, L.D., Durbin, R.M., Garrison, E.P., Kang, H.M., Korbel, J.O., Marchini, J.L., McCarthy, S., McVean, G.A., and Abecasis, G.R. (2015). A global reference for human genetic variation. Nature *526*, 68–74.

Alatrash, G., Garber, H.R., Zhang, M., Sukhumalchandra, P., Qiu, Y., Jakher, H., Perakis, A.A., Becker, L., Yoo, S.Y., Dwyer, K.C., et al. (2017). Cathepsin G is broadly expressed in acute myeloid leukemia and is an effective immunotherapeutic target. Leukemia *31*, 234–237.

Ambrosini, G., Khanin, R., Carvajal, R.D., and Schwartz, G.K. (2014). Overexpression of DDX43 mediates MEK inhibitor resistance through RAS Upregulation in uveal melanoma cells. Mol. Cancer Ther. *13*, 2073–2080.

Azzarelli, R., Simons, B.D., and Philpott, A. (2018). The developmental origin of brain tumours: a cellular and molecular framework. Development *145*, dev162693.

Bantscheff, M., Hopf, C., Savitski, M.M., Dittmann, A., Grandi, P., Michon, A.-M., Schlegl, J., Abraham, Y., Becher, I., Bergamini, G., et al. (2011). Chemoproteomics profiling of HDAC inhibitors reveals selective targeting of HDAC complexes. Nat. Biotechnol. *29*, 255–265.

Carter, R.A., Bihannic, L., Rosencrance, C., Hadley, J.L., Tong, Y., Phoenix, T.N., Natarajan, S., Easton, J., Northcott, P.A., and Gawad, C. (2018). A single-cell transcriptional atlas of the developing murine cerebellum. Curr. Biol. *28*, 2910–2920.e2.

Cavalli, F.M.G., Remke, M., Rampasek, L., Peacock, J., Shih, D.J.H., Luu, B., Garzia, L., Torchia, J., Nor, C., Morrissy, A.S., et al. (2017). Intertumoral heterogeneity within medulloblastoma subgroups. Cancer Cell *31*, 737–754.e6.

Chen, J., Zhu, S., Jiang, N., Shang, Z., Quan, C., and Niu, Y. (2013). HoxB3 promotes prostate cancer cell progression by transactivating CDCA3. Cancer Lett. *330*, 217–224.

Cowan, R., Hoban, P., Kelsey, A., Birch, J.M., Gattamaneni, R., and Evans, D.G. (1997). The gene for the naevoid basal cell carcinoma syndrome acts as a tumour-suppressor gene in medulloblastoma. Br. J. Cancer *76*, 141–145.

Dahmane, N., and Ruiz i Altaba, A. (1999). Sonic hedgehog regulates the growth and patterning of the cerebellum. Development *126*, 3089–3100.

Danovi, D., Falk, A., Humphreys, P., Vickers, R., Tinsley, J., Smith, A.G., and Pollard, S.M. (2010). Imaging-based chemical screens using normal and glioma-derived neural stem cells. Biochem. Soc. Trans. *38*, 1067–1071.

Decock, A., Ongenaert, M., Hoebeeck, J., De Preter, K., Van Peer, G., Van Criekinge, W., Ladenstein, R., Schulte, J.H., Noguera, R., Stallings, R.L., et al. (2012). Genome-wide promoter methylation analysis in neuroblastoma identifies prognostic methylation biomarkers. Genome Biol. *13*, R95.

DePristo, M.A., Banks, E., Poplin, R., Garimella, K.V., Maguire, J.R., Hartl, C., Philippakis, A.A., del Angel, G., Rivas, M.A., Hanna, M., et al. (2011). A framework for variation discovery and genotyping using next-generation DNA sequencing data. Nat. Genet. *43*, 491–498.

Diede, S.J., Yao, Z., Keyes, C.C., Tyler, A.E., Dey, J., Hackett, C.S., Elsaesser, K., Kemp, C.J., Neiman, P.E., Weiss, W.A., et al. (2013). Fundamental differences in promoter CpG island DNA hypermethylation between human cancer and genetically engineered mouse models of cancer. Epigenetics *8*, 1254–1260.

Dobin, A., Davis, C.A., Schlesinger, F., Drenkow, J., Zaleski, C., Jha, S., Batut, P., Chaisson, M., and Gingeras, T.R. (2013). STAR: ultrafast universal RNA-seq aligner. Bioinformatics *29*, 15–21.

Drezner, N.L., and Packer, R.J. (2016). The impact of molecular analysis on the survival of children with embryonal tumors. Transl. Pediatr. *5*, 5–8.

Durinck, S., Moreau, Y., Kasprzyk, A., Davis, S., De Moor, B., Brazma, A., and Huber, W. (2005). BioMart and Bioconductor: a powerful link between biological databases and microarray data analysis. Bioinformatics *21*, 3439–3440.

Falk, A., Koch, P., Kesavan, J., Takashima, Y., Ladewig, J., Alexander, M., Wiskow, O., Tailor, J., Trotter, M., Pollard, S., et al. (2012). Capture of neuroepithelial-like stem cells from pluripotent stem cells provides a versatile system for in vitro production of human neurons. PLoS ONE *7*, e29597.

Fusaki, N., Ban, H., Nishiyama, A., Saeki, K., and Hasegawa, M. (2009). Efficient induction of transgene-free human pluripotent stem cells using a vector based on Sendai virus, an RNA virus that does not integrate into the host genome. Proc. Jpn. Acad., Ser. B, Phys. Biol. Sci. *85*, 348–362.

Gage, F.H. (2000). Mammalian neural stem cells. Science *287*, 1433–1438.

Gibson, P., Tong, Y., Robinson, G., Thompson, M.C., Currle, D.S., Eden, C., Kranenburg, T.A., Hogg, T., Poppleton, H., Martin, J., et al. (2010). Subtypes of medulloblastoma have distinct developmental origins. Nature *468*, 1095–1099.

Goodrich, L.V., Milenković, L., Higgins, K.M., and Scott, M.P. (1997). Altered neural cell fates and medulloblastoma in mouse patched mutants. Science *277*, 1109–1113.

Griesinger, A.M., Birks, D.K., Donson, A.M., Amani, V., Hoffman, L.M., Waziri, A., Wang, M., Handler, M.H., and Foreman, N.K. (2013). Characterization of distinct immunophenotypes across pediatric brain tumor types. J. Immunol. *191*, 4880–4888.

Guo, X., Hollander, L., MacPherson, D., Wang, L., Velazquez, H., Chang, J., Safirstein, R., Cha, C., Gorelick, F., and Desir, G.V. (2016). Inhibition of renalase expression and signaling has antitumor activity in pancreatic cancer. Sci. Rep. *6*, 22996.

Hahn, H., Wicking, C., Zaphiropoulous, P.G., Gailani, M.R., Shanley, S., Chidambaram, A., Vorechovsky, I., Holmberg, E., Unden, A.B., Gillies, S., et al. (1996). Mutations of the human homolog of Drosophila patched in the nevoid basal cell carcinoma syndrome. Cell *85*, 841–851.

Hamilton, S.R., Liu, B., Parsons, R.E., Papadopoulos, N., Jen, J., Powell, S.M., Krush, A.J., Berk, T., Cohen, Z., Tetu, B., et al. (1995). The molecular basis of Turcot's syndrome. N. Engl. J. Med. *332*, 839–847.

Hayashi, Y., Hsiao, E.C., Sami, S., Lancero, M., Schlieve, C.R., Nguyen, T., Yano, K., Nagahashi, A., Ikeya, M., Matsumoto, Y., et al. (2016). BMP-SMAD-ID promotes reprogramming to pluripotency by inhibiting p16/INK4A-dependent senescence. Proc. Natl. Acad. Sci. USA *113*, 13057–13062.

Hooper, J.E., and Scott, M.P. (2005). Communicating with Hedgehogs. Nat. Rev. Mol. Cell Biol. *6*, 306–317.

Horst, R. (2013). UQ eSpace.

Houghton, A.M., Rzymkiewicz, D.M., Ji, H., Gregory, A.D., Egea, E.E., Metz, H.E., Stolz, D.B., Land, S.R., Marconcini, L.A., Kliment, C.R., et al. (2010). Neutrophil elastase-mediated degradation of IRS-1 accelerates lung tumor growth. Nat. Med. *16*, 219–223.

Hovestadt, V., Remke, M., Kool, M., Pietsch, T., Northcott, P.A., Fischer, R., Cavalli, F.M.G., Ramaswamy, V., Zapatka, M., Reifenberger, G., et al. (2013). Robust molecular subgrouping and copy-number profiling of medulloblastoma from small amounts of archival tumour material using high-density DNA methylation arrays. Acta Neuropathol. *125*, 913–916.

Hu, D., Ansari, D., Pawłowski, K., Zhou, Q., Sasor, A., Welinder, C., Kristl, T., Bauden, M., Rezeli, M., Jiang, Y., et al. (2018). Proteomic analyses identify prognostic biomarkers for pancreatic ductal adenocarcinoma. Oncotarget *9*, 9789–9807.

Huang, B., Song, J.H., Cheng, Y., Abraham, J.M., Ibrahim, S., Sun, Z., Ke, X., and Meltzer, S.J. (2016). Long non-coding antisense RNA KRT7-AS is activated in gastric cancers and supports cancer cell progression by increasing KRT7 expression. Oncogene *35*, 4927–4936.

Inanc, M., Ozkan, M., Karaca, H., Berk, V., Bozkurt, O., Duran, A.O., Ozaslan, E., Akgun, H., Tekelioglu, F., and Elmali, F. (2014). Cytokeratin 5/6, c-Met expressions, and PTEN loss prognostic indicators in triple-negative breast cancer. Med. Oncol. *31*, 801.

Johnson, R.L., Rothman, A.L., Xie, J., Goodrich, L.V., Bare, J.W., Bonifas, J.M., Quinn, A.G., Myers, R.M., Cox, D.R., Epstein, E.H., Jr., and Scott, M.P. (1996). Human homolog of patched, a candidate gene for the basal cell nevus syndrome. Science *272*, 1668–1671.

Jones, D.T.W., Jäger, N., Kool, M., Zichner, T., Hutter, B., Sultan, M., Cho, Y.-J., Pugh, T.J., Hovestadt, V., Stütz, A.M., et al. (2012). Dissecting the genomic complexity underlying medulloblastoma. Nature *488*, 100–105.

Jühling, F., Kretzmer, H., Bernhart, S.H., Otto, C., Stadler, P.F., and Hoffmann, S. (2016). metilene: fast and sensitive calling of differentially methylated regions from bisulfite sequencing data. Genome Res. *26*, 256–262.

Karczewski, K.J., Francioli, L.C., Tiao, G., Cummings, B.B., Alföldi, J., Wang, Q., Collins, R.L., Laricchia, K.M., Ganna, A., Birnbaum, D.P., et al. (2019). Variation across 141,456 human exomes and genomes reveals the spectrum of loss-of-function intolerance across human protein-coding genes. bioRxiv. https://doi.org/10.1101/531210.

Kawauchi, D., Robinson, G., Uziel, T., Gibson, P., Rehg, J., Gao, C., Finkelstein, D., Qu, C., Pounds, S., Ellison, D.W., et al. (2012). A mouse model of the most aggressive subgroup of human medulloblastoma. Cancer Cell *21*, 168–180.

Kim, J.-Y., Kim, K.-B., Eom, G.H., Choe, N., Kee, H.J., Son, H.-J., Oh, S.-T., Kim, D.-W., Pak, J.H., Baek, H.J., et al. (2012). KDM3B is the H3K9 demethylase involved in transcriptional activation of lmo2 in leukemia. Mol. Cell. Biol. *32*, 2917–2933.

Koboldt, D.C., Zhang, Q., Larson, D.E., Shen, D., McLellan, M.D., Lin, L., Miller, C.A., Mardis, E.R., Ding, L., and Wilson, R.K. (2012). VarScan 2: somatic mutation and copy number alteration discovery in cancer by exome sequencing. Genome Res. *22*, 568–576.

Koboldt, D.C., Larson, D.E., and Wilson, R.K. (2013). Using VarScan 2 for germline variant calling and somatic mutation detection. Curr. Protoc. Bioinformatics *44*, 15.4.1–15.4.17.

Koch, P., Opitz, T., Steinbeck, J.A., Ladewig, J., and Brüstle, O. (2009). A rosette-type, self-renewing human ES cell-derived neural stem cell with potential for in vitro instruction and synaptic integration. Proc. Natl. Acad. Sci. USA *106*, 3225–3230.

Kool, M., Jones, D.T.W., Jäger, N., Northcott, P.A., Pugh, T.J., Hovestadt, V., Piro, R.M., Esparza, L.A., Markant, S.L., Remke, M., et al.; ICGC PedBrain Tumor Project (2014). Genome sequencing of SHH medulloblastoma predicts genotype-related response to smoothened inhibition. Cancer Cell *25*, 393–405.

Koso, H., Takeda, H., Yew, C.C.K., Ward, J.M., Nariai, N., Ueno, K., Nagasaki, M., Watanabe, S., Rust, A.G., Adams, D.J., et al. (2012). Transposon mutagenesis identifies genes that transform neural stem cells into glioma-initiating cells. Proc. Natl. Acad. Sci. USA *109*, E2998–E3007.

Langmead, B., and Salzberg, S.L. (2012). Fast gapped-read alignment with Bowtie 2. Nat. Methods *9*, 357–359.

Lee, Y., Miller, H.L., Russell, H.R., Boyd, K., Curran, T., and McKinnon, P.J. (2006). Patched2 modulates tumorigenesis in patched1 heterozygous mice. Cancer Res. *66*, 6964–6971.

Lek, M., Karczewski, K.J., Minikel, E.V., Samocha, K.E., Banks, E., Fennell, T., O'Donnell-Luria, A.H., Ware, J.S., Hill, A.J., Cummings, B.B., et al.; Exome Aggregation Consortium (2016). Analysis of protein-coding genetic variation in 60,706 humans. Nature *536*, 285–291.

Li, H. (2013). Aligning sequence reads, clone sequences and assembly contigs with BWA-MEM. arXiv, arXiv:1303.3997. https://arxiv.org/abs/1303.3997.

Li, H., Handsaker, B., Wysoker, A., Fennell, T., Ruan, J., Homer, N., Marth, G., Abecasis, G., and Durbin, R.; 1000 Genome Project Data Processing Subgroup (2009). The Sequence Alignment/Map format and SAMtools. Bioinformatics *25*, 2078–2079.

Li, P., Du, F., Yuelling, L.W., Lin, T., Muradimova, R.E., Tricarico, R., Wang, J., Enikolopov, G., Bellacosa, A., Wechsler-Reya, R.J., and Yang, Z.J. (2013). A population of Nestin-expressing progenitors in the cerebellum exhibits increased tumorigenicity. Nat. Neurosci. *16*, 1737–1744.

Liao, Y., Smyth, G.K., and Shi, W. (2013). The Subread aligner: fast, accurate and scalable read mapping by seed-and-vote. Nucleic Acids Res. *41*, e108.

Lim, S.Y., Yuzhalin, A.E., Gordon-Weeks, A.N., and Muschel, R.J. (2016). Tumor-infiltrating monocytes/macrophages promote tumor invasion and migration by upregulating S100A8 and S100A9 expression in cancer cells. Oncogene *35*, 5735–5745.

Lin, C.Y., Erkek, S., Tong, Y., Yin, L., Federation, A.J., Zapatka, M., Haldipur, P., Kawauchi, D., Risch, T., Warnatz, H.-J., et al. (2016). Active medulloblastoma enhancers reveal subgroup-specific cellular origins. Nature *530*, 57–62.

Lindblad, O., Chougule, R.A., Moharram, S.A., Kabir, N.N., Sun, J., Kazi, J.U., and Rönnstrand, L. (2015). The role of HOXB2 and HOXB3 in acute myeloid leukemia. Biochem. Biophys. Res. Commun. *467*, 742–747.

Love, M.I., Huber, W., and Anders, S. (2014). Moderated estimation of fold change and dispersion for RNA-seq data with DESeq2. Genome Biol. *15*, 550.

Marshall, G.M., Carter, D.R., Cheung, B.B., Liu, T., Mateos, M.K., Meyerowitz, J.G., and Weiss, W.A. (2014). The prenatal origins of cancer. Nat. Rev. Cancer *14*, 277–289.

McKenna, A., Hanna, M., Banks, E., Sivachenko, A., Cibulskis, K., Kernytsky, A., Garimella, K., Altshuler, D., Gabriel, S., Daly, M., and DePristo, M.A. (2010). The Genome Analysis Toolkit: a MapReduce framework for analyzing next-generation DNA sequencing data. Genome Res. *20*, 1297–1303.

McLaren, D., Gorba, T., Marguerie de Rotrou, A., Pillai, G., Chappell, C., Stacey, A., Lingard, S., Falk, A., Smith, A., Koch, P., et al. (2013). Automated large-scale culture and medium-throughput chemical screen for modulators of proliferation and viability of human induced pluripotent stem cell-derived neuroepithelial-like stem cells. J. Biomol. Screen. *18*, 258–268.

Merkle, F.T., Ghosh, S., Kamitaki, N., Mitchell, J., Avior, Y., Mello, C., Kashin, S., Mekhoubad, S., Ilic, D., Charlton, M., et al. (2017). Human pluripotent stem cells recurrently acquire and expand dominant negative P53 mutations. Nature *545*, 229–233.

Millen, K.J., and Gleeson, J.G. (2008). Cerebellar development and disease. Curr. Opin. Neurobiol. *18*, 12–19.

Morris, T.J., Butcher, L.M., Feber, A., Teschendorff, A.E., Chakravarthy, A.R., Wojdacz, T.K., and Beck, S. (2014). ChAMP: 450k chip analysis methylation pipeline. Bioinformatics *30*, 428–430.

Northcott, P.A., Shih, D.J.H., Peacock, J., Garzia, L., Morrissy, A.S., Zichner, T., Stütz, A.M., Korshunov, A., Reimand, J., Schumacher, S.E., et al. (2012). Subgroup-specific structural variation across 1,000 medulloblastoma genomes. Nature *488*, 49–56.

Northcott, P.A., Buchhalter, I., Morrissy, A.S., Hovestadt, V., Weischenfeldt, J., Ehrenberger, T., Gröbner, S., Segura-Wang, M., Zichner, T., Rudneva, V.A., et al. (2017). The whole-genome landscape of medulloblastoma subtypes. Nature *547*, 311–317.

Northcott, P.A., Robinson, G.W., Kratz, C.P., Mabbott, D.J., Pomeroy, S.L., Clifford, S.C., Rutkowski, S., Ellison, D.W., Malkin, D., Taylor, M.D., et al. (2019). Medulloblastoma. Nat. Rev. Dis. Primers *5*, 11.

Oliver, T.G., Read, T.-A., Kessler, J.D., Mehmeti, A., Wells, J.F., Huynh, T.T.T., Lin, S.M., and Wechsler-Reya, R.J. (2005). Loss of patched and disruption of granule cell development in a pre-neoplastic stage of medulloblastoma. Development *132*, 2425–2439.

Özata, D.M., Li, X., Lee, L., Liu, J., Warsito, D., Hajeri, P., Hultman, I., Fotouhi, O., Marklund, S., Ährlund-Richter, L., et al. (2017). Loss of miR-514a-3p regulation of PEG3 activates the NF-kappa B pathway in human testicular germ cell tumors. Cell Death Dis. *8*, e2759.

Pei, Y., Moore, C.E., Wang, J., Tewari, A.K., Eroshkin, A., Cho, Y.-J., Witt, H., Korshunov, A., Read, T.-A., Sun, J.L., et al. (2012). An animal model of MYC-driven medulloblastoma. Cancer Cell *21*, 155–167.

Petitjean, A., Mathe, E., Kato, S., Ishioka, C., Tavtigian, S.V., Hainaut, P., and Olivier, M. (2007). Impact of mutant p53 functional properties on TP53 mutation patterns and tumor phenotype: lessons from recent developments in the IARC TP53 database. Hum. Mutat. *28*, 622–629.

Pollard, S.M., Yoshikawa, K., Clarke, I.D., Danovi, D., Stricker, S., Russell, R., Bayani, J., Head, R., Lee, M., Bernstein, M., et al. (2009). Glioma stem cell lines expanded in adherent culture have tumor-specific phenotypes and are suitable for chemical and genetic screens. Cell Stem Cell *4*, 568–580.

Pöschl, J., Lorenz, A., Hartmann, W., von Bueren, A.O., Kool, M., Li, S., Peraud, A., Tonn, J.C., Herms, J., Xiang, M., et al. (2011). Expression of BARHL1 in medulloblastoma is associated with prolonged survival in mice and humans. Oncogene *30*, 4721–4730.

Pugh, T.J., Weeraratne, S.D., Archer, T.C., Pomeranz Krummel, D.A., Auclair, D., Bochicchio, J., Carneiro, M.O., Carter, S.L., Cibulskis, K., Erlich, R.L., et al. (2012). Medulloblastoma exome sequencing uncovers subtype-specific somatic mutations. Nature *488*, 106–110.

Reble, E., Castellani, C.A., Melka, M.G., O'Reilly, R., and Singh, S.M. (2017). VarScan2 analysis of de novo variants in monozygotic twins discordant for schizophrenia. Psychiatr. Genet. *27*, 62–70.

Reya, T., Morrison, S.J., Clarke, M.F., and Weissman, I.L. (2001). Stem cells, cancer, and cancer stem cells. Nature *414*, 105–111.

Robinson, G., Parker, M., Kranenburg, T.A., Lu, C., Chen, X., Ding, L., Phoenix, T.N., Hedlund, E., Wei, L., Zhu, X., et al. (2012). Novel mutations target distinct subgroups of medulloblastoma. Nature *488*, 43–48.

Robinson, G.W., Orr, B.A., Wu, G., Gururangan, S., Lin, T., Qaddoumi, I., Packer, R.J., Goldman, S., Prados, M.D., Desjardins, A., et al. (2015). Vismodegib exerts targeted efficacy against recurrent Sonic Hedgehog-subgroup medulloblastoma: results from phase II Pediatric Brain Tumor Consortium studies PBTC-025B and PBTC-032. J. Clin. Oncol. *33*, 2646–2654.

Schüller, U., Heine, V.M., Mao, J., Kho, A.T., Dillon, A.K., Han, Y.-G., Huillard, E., Sun, T., Ligon, A.H., Qian, Y., et al. (2008). Acquisition of granule neuron precursor identity is a critical determinant of progenitor cell competence to form Shh-induced medulloblastoma. Cancer Cell *14*, 123–134.

Server, E.V., and Project, N.G.E.S. (2013). Seattle, WA.

Sherry, S.T., Ward, M.H., Kholodov, M., Baker, J., Phan, L., Smigielski, E.M., and Sirotkin, K. (2001). dbSNP: the NCBI database of genetic variation. Nucleic Acids Res. *29*, 308–311.

Shiratsuchi, H., Wang, Z., Chen, G., Ray, P., Lin, J., Zhang, Z., Zhao, L., Beer, D., Ray, D., and Ramnath, N. (2017). Oncogenic potential of CYP24A1 in lung adenocarcinoma. J. Thorac. Oncol. *12*, 269–280.

Siu, I.-M., Bai, R., Gallia, G.L., Edwards, J.B., Tyler, B.M., Eberhart, C.G., and Riggins, G.J. (2008). Coexpression of neuronatin splice forms promotes medulloblastoma growth. Neuro-oncol. *10*, 716–724.

Sturm, D., Witt, H., Hovestadt, V., Khuong-Quang, D.-A., Jones, D.T.W., Konermann, C., Pfaff, E., Tönjes, M., Sill, M., Bender, S., et al. (2012). Hotspot mutations in H3F3A and IDH1 define distinct epigenetic and biological subgroups of glioblastoma. Cancer Cell *22*, 425–437.

Sun, H., Ghaffari, S., and Taneja, R. (2007). bHLH-orange transcription factors in development and cancer. Transl. Oncogenomics *2*, 107–120.

Sun, N.-H., Wang, S.-H., Liu, J.-N., Liu, A., Gong, W.-J., Liu, Y., Sun, P., and Li, H. (2017). The productions of atrial natriuretic peptide and arginine vasopressin in small cell lung cancer with brain metastases and their associations with hyponatremia. Eur. Rev. Med. Pharmacol. Sci. *21*, 4104–4112.

Swartling, F.J., Grimmer, M.R., Hackett, C.S., Northcott, P.A., Fan, Q.-W., Goldenberg, D.D., Lau, J., Masic, S., Nguyen, K., Yakovenko, S., et al. (2010). Pleiotropic role for MYCN in medulloblastoma. Genes Dev. *24*, 1059–1072.

Swartling, F.J., Savov, V., Persson, A.I., Chen, J., Hackett, C.S., Northcott, P.A., Grimmer, M.R., Lau, J., Chesler, L., Perry, A., et al. (2012). Distinct neural stem cell populations give rise to disparate brain tumors in response to N-MYC. Cancer Cell *21*, 601–613.

Tailor, J., Kittappa, R., Leto, K., Gates, M., Borel, M., Paulsen, O., Spitzer, S., Karadottir, R.T., Rossi, F., Falk, A., and Smith, A. (2013). Stem cells expanded from the human embryonic hindbrain stably retain regional specification and high neurogenic potency. J. Neurosci. *33*, 12407–12422.

Tamayo, P., Scanfeld, D., Ebert, B.L., Gillette, M.A., Roberts, C.W.M., and Mesirov, J.P. (2007). Metagene projection for cross-platform, cross-species characterization of global transcriptional states. Proc. Natl. Acad. Sci. USA *104*, 5959–5964.

Terasaki, H., Saitoh, T., Shiokawa, K., and Katoh, M. (2002). Frizzled-10, up-regulated in primary colorectal cancer, is a positive regulator of the WNT - beta-catenin - TCF signaling pathway. Int. J. Mol. Med. *9*, 107–112.

Tsai, W.-C., Lin, C.-K., Yang, Y.-S., Chan, D.-C., Gao, H.-W., Chang, F.-N., and Jin, J.-S. (2013). The correlations of LMX1A and osteopontin expression to the clinicopathologic stages in pancreatic adenocarcinoma. Appl. Immunohistochem. Mol. Morphol. *21*, 395–400.

Wang, V.Y., and Zoghbi, H.Y. (2001). Genetic regulation of cerebellar development. Nat. Rev. Neurosci. *2*, 484–491.

Wang, K., Li, M., and Hakonarson, H. (2010). ANNOVAR: functional annotation of genetic variants from high-throughput sequencing data. Nucleic Acids Res. *38*, e164.

Wechsler-Reya, R.J., and Scott, M.P. (1999). Control of neuronal precursor proliferation in the cerebellum by Sonic Hedgehog. Neuron *22*, 103–114.

Wingate, R.J., and Hatten, M.E. (1999). The role of the rhombic lip in avian cerebellum development. Development *126*, 4395–4404.

Wu, F., Zhang, Y., Sun, B., McMahon, A.P., and Wang, Y. (2017). Hedgehog Signaling: from basic biology to cancer therapy. Cell Chem. Biol. *24*, 252–280.

Xu, X., Nagel, S., Quentmeier, H., Wang, Z., Pommerenke, C., Dirks, W.G., Macleod, R.A.F., Drexler, H.G., and Hu, Z. (2018). KDM3B shows tumor-suppressive activity and transcriptionally regulates HOXA1 through retinoic acid response elements in acute myeloid leukemia. Leuk. Lymphoma *59*, 204–213.

Yang, Z.-J., Ellis, T., Markant, S.L., Read, T.-A., Kessler, J.D., Bourboulas, M., Schüller, U., Machold, R., Fishell, G., Rowitch, D.H., et al. (2008). Medulloblastoma can be initiated by deletion of Patched in lineage-restricted progenitors or stem cells. Cancer Cell *14*, 135–145.

Zhang, X., Liu, G., Ding, L., Jiang, T., Shao, S., Gao, Y., and Lu, Y. (2018). HOXA3 promotes tumor growth of human colon cancer through activating EGFR/Ras/Raf/MEK/ERK signaling pathway. J. Cell. Biochem. *119*, 2864–2874.

Zindy, F., Uziel, T., Ayrault, O., Calabrese, C., Valentine, M., Rehg, J.E., Gilbertson, R.J., Sherr, C.J., and Roussel, M.F. (2007). Genetic alterations in mouse medulloblastomas and generation of tumors de novo from primary cerebellar granule neuron precursors. Cancer Res. *67*, 2676–2684.

# STAR★METHODS

## KEY RESOURCES TABLE

| REAGENT or RESOURCE | SOURCE | IDENTIFIER |
|---|---|---|
| Antibodies | | |
| FLAG | Sigma | Cat# F1804, RRID:AB_262044 |
| MYCN | Santa Cruz Biotechnology | Cat# sc-53993, RRID:AB_831602 |
| GAPDH | Millipore | Cat# CB1001, RRID:AB_2107426 |
| SYNAPTOPHYSIN | Thermo Fisher Scientific | MA5-14532, RRID:AB_10983675 |
| P53 | Agilent | Cat# M7001, RRID:AB_2206626 |
| NESTIN | R&D systems | Cat# MAB1259, RRID:AB_2251304 |
| SOX1 | R&D systems | Cat# AF3369, RRID:AB_2239879 |
| SOX2 | R&D systems | Cat# MAB2018, RRID:AB_358009 |
| PAX6 | Proteintech Group | 12323-1-AP, RRID:AB_2159695 |
| OCT4 | Santa Cruz BIotechnology | Cat# sc-5279, RRID:AB_628051 |
| NANOG | R&D systems | Cat# AF1997, RRID:AB_355097 |
| TUJ1 | R&D systems | Cat# MAB1195, RRID:AB_357520 |
| TBRA | R&D systems | Cat# AF2085, RRID:AB_2200235 |
| SOX17 | R&D systems | Cat# AF1924, RRID:AB_35506 |
| GSE1 | Proteintech | Cat# 24947-1-AP |
| KDM3B | Cell Signaling Technology | Cat# 3100, RRID:AB_1264192 |
| Donkey anti-mouse Alexa Fluor 488 | Thermo Fisher Scientific | Cat# A-21202 |
| Donkey anti-rabbit Alexa Fluor 647 | Thermo Fisher Scientific | Cat # A-31573 |
| Donkey anti-goat Alexa Fluor 647 | Thermo Fisher Scientific | Cat# A32849 |
| Chemicals, Peptides, and Recombinant Proteins | | |
| mTeSR1 | StemCell Technologies, Inc. | Cat# 85850 |
| GelTrex | Thermo Fisher Scientific | Cat# A1413202 |
| DMEM/F-12 + Glutamax | Thermo Fisher Scientific | Cat# (Tailor et al., 2013) |
| N-2 Supplement | Thermo Fisher Scientific | Cat# 17502048 |
| B27 supplement w/o Vitamin A | Thermo Fisher Scientific | Cat# 12587010 |
| Knockout DMEM/F-12 | Thermo Fisher Scientific | Cat# 12660012 |
| Glucose | Thermo Fisher Scientific | Cat# A2494001 |
| Neurobasal-A medium | Thermo Fisher Scientific | Cat# A2477501 |
| Knockout serum replacement | Thermo Fisher Scientific | Cat# 10828028 |
| 2-Mercaptoethanol | Thermo Fisher Scientific | Cat# 31350010 |
| DPBS (without calcium/magnesium) | Thermo Fisher Scientific | Cat#14190-144 |
| DPBS (with calcium/magnesium) | Thermo Fisher Scientific | Cat#14040-133 |
| Accutase | Innovative Cell Technologies | Cat# AT-104 |
| Thiazovivin | Stemcell technologies | Cat# 72254 |
| SB431542 | StemRD | Cat# SB-050 |
| LDN-193189 | Stemgent | Cat# 04-0074 |
| Laminin | Sigma Aldrich | Cat# L2020 |
| Poly-l-ornithine hydrobromide | Sigma Aldrich | Cat# P3655 |
| bFGF | Peprotech | Cat# 100-18B |
| EGF | Peprotech | Cat# 100-15 |
| TrypLE Express | Thermo Fisher Scientific | Cat# 12604013 |
| Glutamax | Thermo Fisher Scientific | *Cat# 35050061* |
| Trypsin-EDTA | Thermo Fisher Scientific | *Cat# 25300054* |
| Collagenase, Type IV | Thermo Fisher Scientific | Cat#17104019 |

(*Continued on next page*)

*Continued*

| REAGENT or RESOURCE | SOURCE | IDENTIFIER |
|---|---|---|
| DMEM | Thermo Fisher Scientific | Cat# 11965-092 |
| FBS | Seradigm | Cat# 1500-500 |
| ViralBoost | Alstem | Cat# VB100 |
| Lentivirus Precipitation Solution | Alstem | Cat# VC100 |
| TransIT-Lenti | Mirus Bio | Cat# 6603 |
| SHH (C-24II) | GenScript | Cat# Z03067 |
| Wnt3a | R&D systems | Cat# 5036-WN |
| GDF7 | R&D systems | Cat# 8386-G7 |
| DAPI | Thermo Fisher Scientific | Cat# D1306 |
| BMP7 | R&D Systems | Cat#354-BP |
| EpiLife Medium with 60uM Calcium | Thermo Fisher Scientific | Cat # M-EPI-500-CA |
| EpiLife Defined Growth Supplement (EDGS) | Thermo Fisher Scientific | Cat # S-012-5 |
| Recombinant human collagen type 1 Coating Matrix kit | Thermo Fisher Scientific | Cat# R-011-K |
| Accuprime HiFi | Thermo Fisher Scientific | *Cat# 12346094* |
| Puromycin | Sigma Aldrich | Cat# P9620 |
| GelRed | Biotium | Cat# 41002 |
| Vilo Superscript | Thermo Fisher Scientific | *Cat# 11755050* |
| SYBR | KAPA Biosystems | Cat# KK4600 |
| Critical Commercial Assays | | |
| Click-iT EdU Alexa Fluor 647 Flow Cytometry Assay kit | Thermo Fisher Scientific | Cat # C10424 |
| Cyquant Direct Cell Proliferation Assay | Thermo Fisher Scientific | Cat# C35011 |
| AllPrep DNA/RNA Mini Kit | QIAGEN | Cat# 80204 |
| Surveyor mutation detection kit | Integrated DNA Technologies | Cat# 706020 |
| Quick DNA miniprep kit | Zymo research | Cat# D3024 |
| Quick RNA miniprep kit | Zymo research | Cat #R1054 |
| Agilent RNA 6000 Nano kit | Agilent | Cat# 5067-1511 |
| Deposited Data | | |
| Mouse granule neural precursor transcriptome data | https://www.ncbi.nlm.nih.gov/pubmed/30220501 | ENA: PRJEB23051 |
| Raw RNaseq | This paper | EGAS00001003620 |
| Raw Whole Exome Sequencing | This paper | EGAS00001003620 |
| Raw amplicon sequencing | This paper | EGAS00001003620 |
| GTML tumor RNA expression data | https://www.ncbi.nlm.nih.gov/pubmed/22624711 | GEO: GSE36594 |
| Human brain tumor expression data | https://www.ncbi.nlm.nih.gov/pubmed/24078694 | GEO: GSE50161 |
| Human medulloblastoma tumor subgroup expression data | https://www.ncbi.nlm.nih.gov/pubmed/28609654 | GEO: GSE85217 |
| Experimental Models: Cell Lines | | |
| WTC10 iPSC | Conklin Lab | https://www.ncbi.nlm.nih.gov/pubmed/27794120 |
| Gorlin 1 iPSC | This paper | N/A |
| Gorlin 2 iPSC | This paper | N/A |
| WTC10 NES cells | This paper | N/A |
| Control NES cells | This paper | N/A |
| Gorlin 1 NES cells | This paper | N/A |
| Gorlin 2 NES cells | This paper | N/A |
| Sai2 NES cells | Smith Lab | https://www.ncbi.nlm.nih.gov/pubmed/23884946 |

(*Continued on next page*)

| ***Continued*** | | |
|---|---|---|
| REAGENT or RESOURCE | SOURCE | IDENTIFIER |
| Oligonucleotides | | |
| GSE1 sgRNA (TTGGAGCGATGGTCACCACG) | Integrated DNA Technologies | N/A |
| KDM3B sgRNA (GCAGAACTGGTCCCCAACAT) | Integrated DNA Technologies | N/A |
| GSE1 surveyor F (ctgcacgtggctgtcact); GSE1 surveyor R (actcaacctcgaaagctcca) | Integrated DNA Technologies | N/A |
| KDM3B surveyor F (gctcctgcgatttaccatgt); KDM3B surveyor R (ccccaatcttcccgttaagt) | Integrated DNA Technologies | N/A |
| GLi1 qPCR F (cagggaggaaagcagactga); GLI1 qPCR R (Actgctgcaggatgactgg) | Integrated DNA Technologies | N/A |
| GAPDH qPCR F (CCATGGGGAAGGTGAAGGTC); GAPDH qPCR R (TGAAGGGGTCATTGATGGCA) | Integrated DNA Technologies | N/A |
| Recombinant DNA (plasmids) | | |
| pLentiCRISPR v2 | Addgene | RRID:Addgene_52961 |
| pCDH-CAG-3xFLAG-MYCN-mScarlet-Luciferase | This paper | N/A |
| pCDH-CAG-mScarlet-Luciferase | This paper | N/A |
| pCDH-CAG-3xFLAG-DDX3X (WT)-EF1a-Luciferase-Blast | This paper | N/A |
| pCDH-CAG-3xFLAG-DDX3X (R534S)-EF1a-Luciferase-Blast | This paper | N/A |
| pCDH-CAG-3xFLAG-DDX3X (R351W)-EF1a-Luciferase-Blast | This paper | N/A |
| pCDH-CAG-3xFLAG-DDX3X (R534S)-EF1a-Luciferase-Blast | This paper | N/A |
| pCDH-CAG-GSE1 (silent mut)-EF1a- Blast | This paper | N/A |
| Software and Algorithms | | |
| Chip Analysis Methylation Pipeline (ChAMP v. 2.6.4) | Morris et al., 2014 | https://bioconductor.org/packages/release/bioc/html/ChAMP.html |
| Metilene (v. 0.26) | Jühling et al., 2016 | https://www.bioinf.uni-leipzig.de/Software/metilene/ |
| Samtools | Li et al., 2009 | http://samtools.sourceforge.net/ |
| Burrows-Wheeler Aligner | Li, H. 2013 | http://bio-bwa.sourceforge.net/ |
| Picard tools | Broad Institute | https://broadinstitute.github.io/picard/ |
| Mutect2 (GATK4) | McKenna et al., 2010; DePristo et al., 2011; Reble et al., 2017 | https://github.com/broadinstitute/gatk/ |
| Varscan2 | Koboldt et al., 2012; Koboldt et al., 2013. | http://varscan.sourceforge.net/ |
| Annovar | Wang et al., 2010 | http://annovar.openbioinformatics.org/en/latest/ |
| dbSNP | Sherry et al., 2001 | https://www.ncbi.nlm.nih.gov/snp/ |
| Exome Aggregation Consortium (ExAC) | Lek et al., 2016 | http://exac.broadinstitute.org/ |
| Exome Sequencing Project (ESP) | NHLBI Exome sequencing project, 2019 | https://evs.gs.washington.edu/EVS/ |
| 1000 Genomes Project | 1000 Genomes Project Consortium et al., 2015 | http://www.internationalgenome.org/ |
| STAR | Dobin et al., 2013 | https://github.com/alexdobin/STAR |
| DESeq | Love et al., 2014 | https://bioconductor.org/packages/release/bioc/html/DESeq2.html |
| Conumee | Hovestadt et al., 2013 | https://www.bioconductor.org/packages/release/bioc/html/conumee.html |
| Bowtie2 | Langmead and Salzberg, 2012 | http://bowtie-bio.sourceforge.net/bowtie2/index.shtml |
| **Script for Metagene projection** | Tamayo et al., 2007 | https://www.ncbi.nlm.nih.gov/pubmed/17389406 |

(*Continued on next page*)

| *Continued* | | |
|---|---|---|
| REAGENT or RESOURCE | SOURCE | IDENTIFIER |
| Subread (v 1.5.2) | Liao et al., 2013 | http://subread.sourceforge.net/ |
| biomaRt (v 2.34.2) | Durinck et al., 2005 | http://bioconductor.org/packages/release/bioc/html/biomaRt.html |
| Other | | |

## LEAD CONTACT AND MATERIALS AVAILABILITY

Further information and requests for reagents may be directed to and will be fulfilled by the Lead Contact, Dr. William A. Weiss (waweiss@gmail.com).

## EXPERIMENTAL MODEL AND SUBJECT DETAILS

### Animals

Immunocompromised (NOD-scid IL2Rgamma$^{null}$ or NSG) 6-8 week old female mice used for transplantation were purchased from Jackson Labs. Mice were maintained in the Animal Facility at UCSF. All experiments were performed in accordance with national guidelines and regulations, and with the approval of the IACUC at UCSF. 300,000 cells in 5uL of NES cell medium were injected per mouse. Injections were performed using a stereotactic machine starting from lambda 2mm right, 2mm down and 2mm deep. Mice were euthanized at endpoint, which was either signs of tumor growth (e.g., hunched back, weight loss, head tilt, etc) or 1 year post transplantation.

### iPSC culture

Male WTC10 iPSC (Hayashi et al., 2016) were maintained on GelTrex coated 6-well plates in mTeSR1 media in a humidified 37°C incubator with 5% $CO_2$. Cells were passaged every 4-5 days using Accutase and plated at 200-300,000 cells/well of a 6-well plate in 2mL mTeSR1 with 2uM of Thiazovivin. The Gorlin iPSC (male Gorlin 1 derived from KAS537 keratinocytes, female Gorlin 2 derived from KAS573 keratinocytes) and female control (derived from KTM1 keratinocytes) were maintained on MEF feeders in KSR medium (DMEM/F12, knockout serum replacement (KSR) 20%, L-glutamine 2mM, NEAA (0.1mM), 2-mercaptoethanol (0.1mM) supplemented with FGF2 (10ng/ml). The iPSC colonies were passaged 1:3 every 4-5 days using dissociation buffer (1mM calcium chloride, 0.025% trypsin, 1mg/ml collagenase IV, 1:5 KSR media, made in PBS solution). Gorlin iPSC were authenticated by Sanger sequencing of genomic DNA for *PTCH1* at c.G1762 (Gorlin 1) and c.C1925 (Gorlin 2). All experiments using iPSC in the William A Weiss lab (UCSF) were approved by Human Gamete, Embryo and Stem Cell Research Committee of the UCSF Stem Cell Research Oversight Committee. All experiments using iPSC in the Austin Smith lab (University of Cambridge) were performed under ethical approval from the Cambridgeshire Research Ethics Committee (Reference 96/085). The storage and use of human tissue was approved by the Human Tissue Authority, UK (License number 12196). Although we observed a difference in tumorigenic penetrance using a male (Gorlin 1) and female (Gorlin 2) NES cells (Figure 4B), this is likely due to differences in transcriptomes unrelated to the sex identity.

## METHOD DETAILS

### Gorlin iPSC derivation

Gorlin (KAS537, KAS573) and normal human keratinocytes (KTM1) were obtained from patients and healthy controls respectively as previously described (Hovestadt et al., 2013; Sturm et al., 2012). Primary human adult keratinocytes obtained from Thierry Magnaldo's laboratory were cultured in recombinant human collagen type I- plated tissue culture flasks in EpiLife Medium supplemented with EpiLife Defined Growth Supplement (EDGS). Transgene-free reprogramming of keratinocytes to iPSC was achieved using Sendai virus (SeV), a negative sense single-stranded RNA virus that replicates in the cytoplasm without a DNA phase (Fusaki et al., 2009). In brief, approximately $5 \times 10^4$ to $10 \times 10^4$ keratinocytes were seeded per well of a six well plate in EpiLife media supplemented with EDGS. Each well was then infected with separate SeV constructs carrying the pluripotency genes Oct4, Klf4, Sox2 and c-Myc at a multiplicity of infection (MOI) of 5 for 24 hours in 1ml EpiLife media per well. Virus containing media was removed and the cells were washed gently twice with PBS and replaced with fresh Epilife media. On day 4 post-infection, keratinocytes were collected, counted and seeded at a density of $4 \times 10^5$ initially in EpiLife media on inactivated MEFs. Epilife media was replaced with KSR medium supplemented with FGF2 (10ng/ml) after a further 24 hours. KSR/FGF2 media was changed every two days and pluripotent stem cell colonies emerged after 4-6 weeks. Keratinocyte derived iPSC were picked and expanded on MEF feeders in KSR/FGF2 media. Informed consent was obtained from all subjects.

## Teratoma formation assay

Human iPSC were injected into the kidney capsule of NOD/SKID mice using a protocol adapted from (Morris et al., 2014). Mice were sacrificed after 3 months, or when the mice developed a visible abdominal mass. The teratomas were fixed with 10% formalin and prepared for paraffin embedded sections.

## Embryoid body formation and differentiation

iPSC colonies were floated in KSR media (without FGF2) on untreated tissue culture plates at 37 degrees for 1-2 weeks. The embryoid bodies were then plated on tissue culture flasks coated with poly-L-ornithine and laminin in N2B27 media (1:1 Neurobasal:DMEM/F12, L-glutamine 2mM, N-2 supplement 0.5x, B27 w/o vitamin A supplement 0.5x, 2-mercaptoethanol (0.1mM)) for further 1-2 weeks.

## Differentiation to and maintenance of neuroepithelial stem (NES) cells

iPSC differentiation to NES cells was performed as previously described (Koch et al., 2009). Briefly, iPSC were cultured as embryoid bodies in KSR medium with SB431542 (10uM), LDN193189 (500nM) for 3 days, N2 medium (DMEM/F-12 (0.5x) & Neurobasal medium (0.5x), N2 supplement (0.5x), B27 supplement (0.5x)) for 6 days and plated on poly-L-ornithine/laminin coated plates for another 6 days in N2 medium. Rosettes were picked, dissociated in Accutase for 5 min at 37°C and plated on poly-L-ornithine/laminin coated wells in NES cell medium (DMEM/F-12, Glutamax, 1x N2 supplement, 0.05x B27 w/o vitamin A supplement, 1.6g/L Glucose, 10-20ng/mL EGF, 10-20ng/mL FGF2) in a humidified 37°C incubator with 5% $CO_2$. NES cells were fed daily and passaged every 3-4 days using TrypLE Express and plated at 500-600,000 cells/well of a 6-well plate in 2mL NES cell medium.

## Spontaneous and direct differentiation of NES cells

NES cells were differentiated spontaneously or directly in Figure 5. For spontaneous differentiation, NES cells were cultured in N2 medium for 2 days. For direct differentiation, NES cells were grown in N2 medium supplemented with Wnt3a (20ng/mL) and GDF7 (100ng/mL) for 2 days.

## CRISPR plasmid and mutation detection

sgRNA targeting GSE1 and KDM3B were designed using http://crispor.org and cloned into pLentiCRISPR v2. After NES cells were transduced with plasmids encoding Cas9 and sgRNA, we selected the cell population with puromycin. Genomic DNA was extracted, and PCR was performed across the targeted junction using Accuprime HiFi Taq. Primers for sgRNA and PCR were obtained from IDT Technologies. PCR products were digested using Surveyor Nuclease mutation detection kit and visualized on 10% TBE gels stained in GelRed. To identify mutations and quantitate efficiency of mutations, amplicon sequencing was performed using services provided by GENEWIZ (https://www.genewiz.com/en).

## Karyotype analysis

G-band karyotype analysis of NES cells were analyzed by Cincinnati Children's Hospital (https://www.cincinnatichildrens.org/service/d/diagnostic-labs/cytogenetics),

## RT-qPCR

RNA was extracted using an RNA extraction kit (Zymo Research). 500ng of RNA was converted to cDNA using Vilo Superscript (Thermo Fisher) in a 20uL final volume and the following settings: 25°C for 10min, 42°C for 60 min and 85°C for 5 min. cDNA was then diluted in 80uL of water and qPCR was performed in a 384 well plate using SYBR green (KAPA Biosystems) on an AB7900HT machine with the following settings: 95°C for 1 min, 40 cycles (95°C for 3 s and 60°C for 1 min). Each qPCR reaction occurred in final volume of 10uL containing 5uL SYBR mastermix, 5uM of each forward and reverse primer, and 0.4uL of the cDNA. Gene expression was normalized to GAPDH and represented as fold increase over control cell lines.

## Cell proliferation assays

EdU and CyQuant Direct cell proliferation kits were used according to manufacturer protocol. For EdU assays, cells were treated with 10uM EdU for 1hours, fixed and undergone Click-iT chemistry to stain for EdU in cells. Subsequently, cells were counterstained in DAPI and run on BD FACSAria III to obtain the cell populations in G1, S and G2/M phase. For CyQuant Direct, cells were plated on 3-4 poly-l-ornithine/laminin coated 96 well plates at 20,000 cells per well. Each day, one plate was fixed and stained using the CyQuant Direct nucleic acid stain and background suppression dye and analyzed on a plate reader for green fluorescence in the nucleic acid stain. Raw data was normalized to Day 1 measurements to determine the percentage of increase in cells on subsequent days.

## RNA-seq

Total RNA was extracted from flash frozen tissue using a AllPrep DNA/RNA Mini Kit. Quality of total RNA samples was checked on an Agilent Bioanalyzer 2100 RNA Nano chip (Agilent). RNA samples with RNA Integrity Numbers of at least 7 were sent to Novogene (https://en.novogene.com/) for library preparation (polyA enrichment) and RNA sequencing (150 base pair Paired End reads, 50 million reads total).

### Differential gene expression analysis of NES cells and tumors derived from NES cells

To examine transcriptomic differences, cDNA reads were aligned to hg19 using STAR alignment(v2.6.1) to generate bam files (Dobin et al., 2013). Unnormalized gene read counts were generated using STAR. Differentially expressed genes were normalized and analyzed using the DESeq2(v1.22.1) package in R(v3.5.1) (Love et al., 2014).

### Comparison of transcriptomes of tumors from NES cells with different brain tumor types and medulloblastoma subgroups

Raw RNA-seq reads were mapped to the human genome assembly GRCh37/hg19 using STAR v2.5.3a. The alignment was performed with a two-pass approach, where splice junctions discovered during a first alignment guide the forming of a final second alignment. Finally, reads unmapped by STAR were subjected to a final round of alignment via bowtie2 2.3.4.3 (Langmead and Salzberg, 2012). Read counts for genes were extracted using the featureCounts function from the subread 1.5.2 package and utilizing h19 gene annotations from GENCODE (Liao et al., 2013). Subsequently, read counts were converted to measures of transcripts per million (TPM). Ensembl gene ids were translated to official gene symbols (HUGO Gene Nomenclature Committee; HGNC) in R using the biomaRt package (Durinck et al., 2005).

### Microarray expression preprocessing

CEL files from three microarray expression datasets were downloaded from GEO: medulloblastoma samples with subgroup annotation (GEO: GSE85217, 763 samples, (Cavalli et al., 2017)), brain tumors & normal brain (GEO: GSE50161, 130 samples, (Griesinger et al., 2013)), GTML (GEO: GSE36594, 32 samples, (Swartling et al., 2012)). CEL files were preprocessed with the Robust Multichip Average (RMA) protocol in the Affymetrix Expression Console (AEC). Gene symbols in the resulting gene expression matrix were translated to official HGNC gene symbols and multiple rows associated with the same gene symbol were collapsed using the average.

### Cross-dataset classifications

Tumor samples were classified and compared with other brain tumors or medulloblastoma subgroups in R using a package for cross-platform comparisons of transcriptional profiles (Tamayo et al., 2007). Specifically, the script was employed using either the GEO: GSE85217 or GEO: GSE50161 as model and then projecting the gene expression data of tumor samples onto these model data, respectively. From the various outputs generated by the R package, we employed the hierarchical clustering plot, principal component analyses plot, and results of the support vector machine classification in order to characterize our tumor samples.

### Granular Neural Precursor (GNP) comparison

Raw single cell RNA-seq data of GNP cells from normal mouse cerebellum were obtained from ENA: PRJEB23051 (Carter et al., 2018). Clusters 21 and 22 were found to be indicative of GNP cells and were subsequently compared to RNA-sequencing of our NES cells. Differentially expressed genes were analyzed using the DESeq2(v1.22.1) package in R(v3.5.1) (Love et al., 2014). To account for the coverage difference in single cell versus bulk RNA-sequencing, we elected to not filter for coverage in GNP expression comparisons.

### Whole Exome Sequencing (WES) and variant discovery

Genomic DNA was isolated from tumors and NES cells using AllPrep DNA/RNA Mini Kit. Quality of genomic DNA was checked on a 1% agarose gel. Exome capture, library preparation and whole exome sequencing (150 base pair Paired End reads at 100x coverage) was conducted using Agilent SureSelect Human All Exon V6 Kit by Novogene (https://en.novogene.com/).

To examine genomic variants, exome paired-end reads were aligned to hg19/grch37 using BWA-MEM(v0.7.15) and sorted using SAMtools to produced sorted-mapped bams (Li, 2013; Li et al., 2009). The sorted-mapped bams were processed by marking duplicates and recalibrating bases using Picard tools(v2.18.25) (http://broadinstitute.github.io/picard/). Single nucleotide variants and indels (insertions and deletions) were called using Mutect2(v4.1) and Varscan2(v2.4.3) following best practices (DePristo et al., 2011; Koboldt et al., 2013; 2012; McKenna et al., 2010; Reble et al., 2017). Somatic variants were called using NES cells as the normal sample and the transformed or implanted cells as the tumor sample. Tumor-only variants of each sample were also called in tumor-only mode. Somatic and tumor-only variants were annotated using annovar(v.2015) (Wang et al., 2010). Population frequency of each variant was gathered from multiple databases (1000 Genomes Project Consortium et al., 2015; Horst, 2013; Karczewski et al., 2019; Lek et al., 2016; Server and Project, 2013; Sherry et al., 2001).

### Copy number variation analysis

DNA was extracted from tumors and analyzed for genome-wide DNA methylation patterns using the Illumina HumanMethylationEPIC BeadChip (850k) array. Processing of DNA methylation data was performed with custom approaches as previously described (Hovestadt et al., 2013; Sturm et al., 2012), and copy number profiles were generated using the ‘conumee’ package for R (https://www.bioconductor.org/packages/release/bioc/html/conumee.html).

### Methylation array analysis

Raw methylation array data were processed, filtered, and normalized using the Chip Analysis Methylation Pipeline (ChAMP v. 2.6.4) (Morris et al., 2014) within Bioconductor and in R (v. 3.3.3). Human cerebellum and human medulloblastoma tumors were processed separately using 450K methods whereas iPSC-based tumors used EPIC methods. Overlapping normalized beta values from probes that passed QC (n = 348,212) were combined from all groups. Differential methylation analysis was performed using metilene (v. 0.26)(Jühling et al., 2016) with a minimum CpG length of 10, a minimum absolute difference of 0.1, and adjusted p values < 0.05. Overlapping DMRs were identified using the GenomicRanges package (v 1.26.4).

## QUANTIFICATION AND STATISTICAL ANALYSIS

### Mutational rate estimates

The mutational rate of the NES cell tumors were determined by extracting the nonsynonymous, synonymous, stop modulating, somatic variants; these variants are further filtered by excluding variants found above 1% in population frequency databases (1000 Genomes Project Consortium et al., 2015; Horst, 2013; Karczewski et al., 2019; Lek et al., 2016; Server and Project, 2013; Sherry et al., 2001). Somatic variants were filtered for false positives by excluding variants with fewer than 10 reads and at a variant allele frequency of 15% or lower. The mutational rate was estimated by dividing the remaining somatic variants by the size of the exome (30 megabases).

### Quantitation of IHC for p53 and FLAG-MYCN

For quantitation of nuclear p53 and FLAG-MYCN staining in IHC, the number of positive cells was divided by the number of total cells. Positive and total cells were counted manually. A minimum of three fields of view were analyzed per tumor, with approximately 400 cells in each field of view. Data represent mean ± standard error of mean.

### Statistical analysis

For qPCR, EdU and CyQuant Direct Cell proliferation assays, data points represent the average of 3 independent experiments ± standard error of mean and p values were generated by one-tailed t test (unequal variance) For survival curves, p values were calculated by Log-rank (Mantel-Cox test) using GraphPad Prism. For all statistical analyses, p value less than 0.05 was interpreted as statistically significant.

## DATA AND CODE AVAILABILITY

Raw data for whole exome sequencing, RNA-seq and amplicon sequencing are stored in the European Genome Archive (https://ega-archive.org). The accession number for the sequencing data reported in this paper is EGA: EGAS00001003620. Previously published datasets used are listed in the Key Resources Table.

Cell Stem Cell, Volume *25*

## Supplemental Information

# Engineering Genetic Predisposition

# in Human Neuroepithelial Stem Cells

# Recapitulates Medulloblastoma Tumorigenesis

**Miller Huang, Jignesh Tailor, Qiqi Zhen, Aaron H. Gillmor, Matthew L. Miller, Holger Weishaupt, Justin Chen, Tina Zheng, Emily K. Nash, Lauren K. McHenry, Zhenyi An, Fubaiyang Ye, Yasuhiro Takashima, James Clarke, Harold Ayetey, Florence M.G. Cavalli, Betty Luu, Branden S. Moriarity, Shirin Ilkhanizadeh, Lukas Chavez, Chunying Yu, Kathreena M. Kurian, Thierry Magnaldo, Nicolas Sevenet, Philipp Koch, Steven M. Pollard, Peter Dirks, Michael P. Snyder, David A. Largaespada, Yoon Jae Cho, Joanna J. Phillips, Fredrik J. Swartling, A. Sorana Morrissy, Marcel Kool, Stefan M. Pfister, Michael D. Taylor, Austin Smith, and William A. Weiss**

**SUPPLEMENTARY FIGURES**

**Figure S1. Expression of FLAG-MYCN and p53 in WTC10 *MYCN* tumors. (Related to Figure 1).** Immunohistochemical staining of WTC10 *MYCN* tumors show **(A)** positive staining for FLAG (MYCN), and **(B)** negative staining for p53. Scale bars indicate 20um. **(C)** Quantitation of staining for FLAG and p53. A minimum of three fields of 400x view were analyzed per tumor, with approximately 400 cells in each field of view. Data represent mean + SEM.

**Figure S2*.* Hierarchical clustering analysis of WTC10 *MYCN* tumors (Related to Figures 1 and 2).** Hierarchical clustering of transcriptomes of WTC10 *MYCN* tumors with **(A)** various pediatric brain tumors and **(B)** four major medulloblastoma subgroups showing WTC10 *MYCN* tumors resemble SHH medulloblastoma.

**Figure S3. Keratinocytes from Gorlin syndrome patients can be converted to iPSC (Related to Figure 3)**. **(A)** Phase contrast images of keratinocytes from a healthy control (WT) and a patient with Gorlin syndrome (Gorlin 1). **(B)** Table showing the PTCH1 status in WT and Gorlin keratinocytes. **(C)** Protocol for reprogramming keratinocytes to iPSC with Sendai virus. **(D)** Both Gorlin iPSC clones expressed pluripotency markers OCT4, SOX2, and NANOG and showed typical morphology of human iPSCs. **(E)** Differentiated embryoid bodies from Gorlin 1 iPSC were evaluated for markers of the three germ layers. Images shown are (left) immunofluorescence detection for markers of the neuroectoderm (SOX1, TUJ1), mesoderm (TBRA) and endoderm (SOX17), and (right) phase contrast images. **(F)** Teratoma after injection into kidney capsules of NOD/SCID mice contain tissue of all three germ layers including neuroectodermal rosettes (ectoderm) (i), intestinal epithelium (endoderm) (ii), and mesenchymal tissue (mesoderm) (iii). **(G)** Phase contrast image of NES cells derived from Gorlin 1 iPSC, displaying typical rosette forming features of NES cells. **(H)** RT-qPCR analysis of NES cell lines derived from Gorlin 1 and Gorlin 2 iPSC show typical neuroepithelial (SOX1), rosette-stage (PLZF1, MMNR1) and hindbrain regional markers (GBX2) similar to that found in fetal hindbrain NES cells (Sai2) (Tailor et al., 2013), and low expression of radial-glial markers (GFAP, S100B). (Scale bars: D – 100um, E – 50um (top four panels), 75uM (lower two panels), G – 50um).

**Figure S4. Hierarchical clustering analysis of Gorlin 1 tumors for brain tumor type and medulloblastoma subgroup (Related to Figure 4).** Hierarchical clustering of transcriptomes of Gorlin 1 tumors with **(A)** pediatric brain tumors and **(B)** four major medulloblastoma subgroups. Gorlin tumors align with SHH medulloblastoma.

**Figure S5. Hierarchical clustering analysis of Gorlin 1 *DDX3X* mutant and *GSE1*$^{-/-}$ tumors for medulloblastoma subgroup (Related to Figures 6 and 7).** Hierarchical clustering analysis of transcriptomes comparing **(A)** Gorlin *DDX3X* mutant tumors, and **(B)** Gorlin *GSE1*$^{-/-}$ tumors with the four major subgroups of medulloblastoma.

**Figure S6. Characterization of GSE1 and KDM3B expression in medulloblastoma survival and mutations generated in NES cells (Related to Figure 7). (A)** Kaplan Meier curves of human medulloblastoma patients stratified by expression levels for *GSE1* or *KDM3B*. **(B)** Gorlin 1 NES cells were transduced with Cas9 and either control (Ctrl) sgRNA or sgRNA binding *GSE1* or *KDM3B*. Genomic DNA was extracted and PCR was performed using primers specific to the intended cut site on *GSE1* or *KDM3B.* PCR products were digested with Surveyor nuclease and run on a 10% TBE gel. Cleavage products confirmed indels at the desired locations. **(C)** Quantitation of the number of alleles mutated or showing a frameshift at *GSE1* and *KDM3B* (50,000 total reads per gene). **(D)** Examples from amplicon sequencing of mutations near the sgRNA target regions for *GSE1* and *KDM3B*. **(E)** Schematic of GSE1 construct showing silent mutations preventing sgRNA binding while expressing WT amino acid sequence for GSE1.

**Figure S7: Copy number variations in MYCN and Gorlin 1 tumors (Related to Figure 2, and Figure 4)** Representative copy number analysis of **(A)** *MYCN* tumors and **(B)** Gorlin 1 tumors showing no chromosome copy number changes.

Figure S1

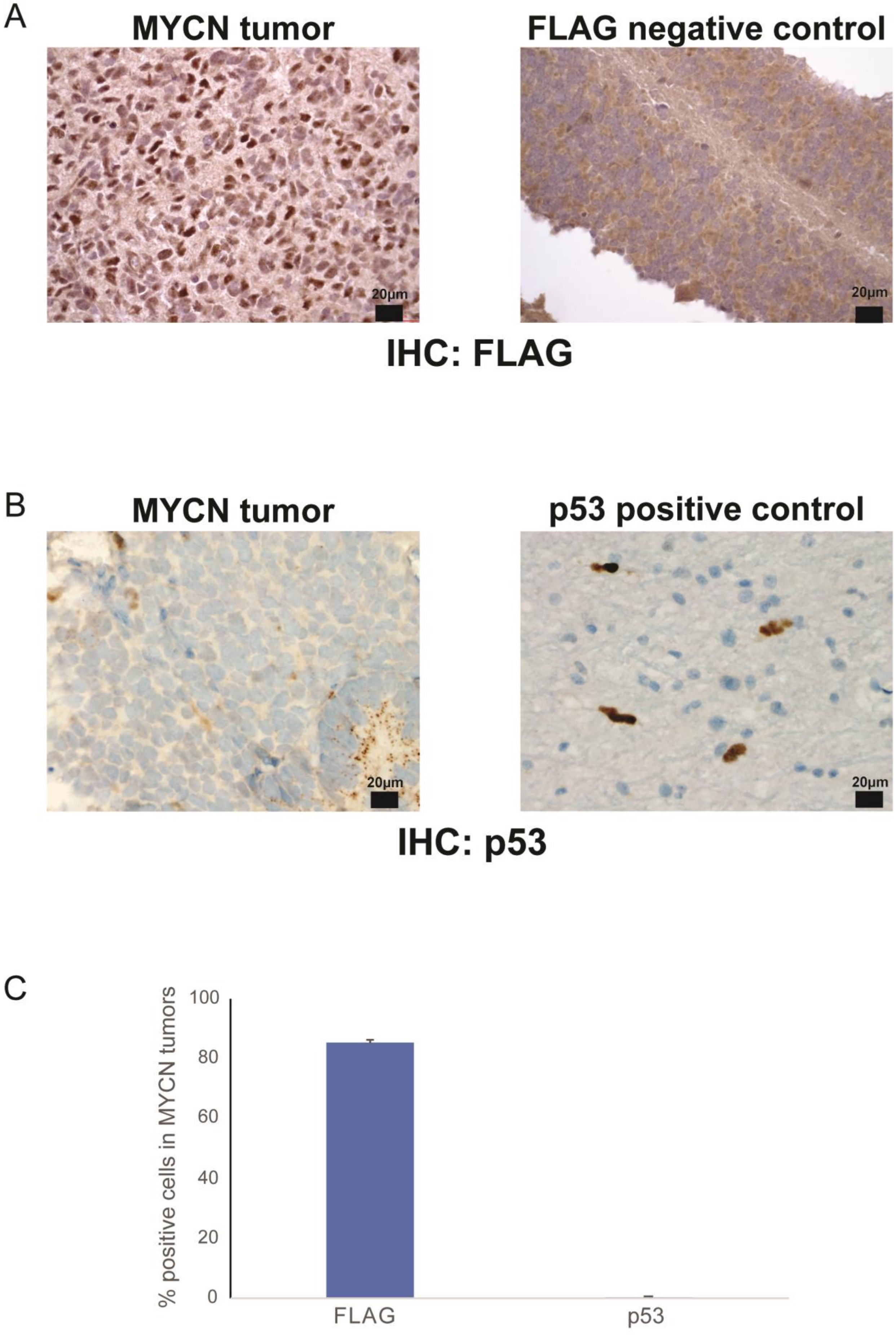
A
MYCN tumor
FLAG negative control
20μm
20μm
IHC: FLAG
B
MYCN tumor
p53 positive control
20μm
20μm
IHC: p53
C
% positive cells in MYCN tumors
100
80
60
40
20
0
FLAG
p53

# Figure S2

A

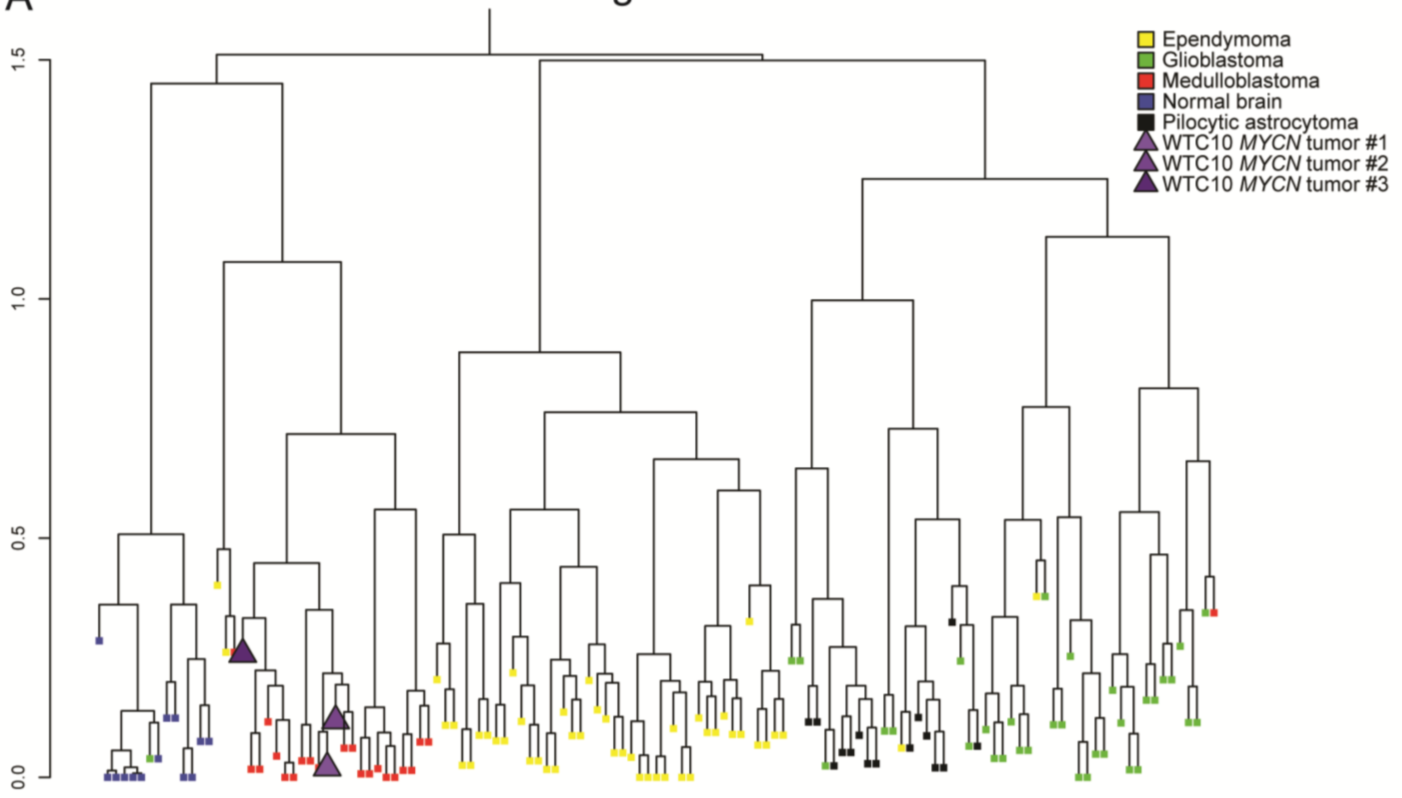

1.5
1.0
0.5
0.0
Ependymoma
Glioblastoma
Medulloblastoma
Normal brain
Pilocytic astrocytoma
WTC10 *MYCN* tumor #1
WTC10 *MYCN* tumor #2
WTC10 *MYCN* tumor #3


B

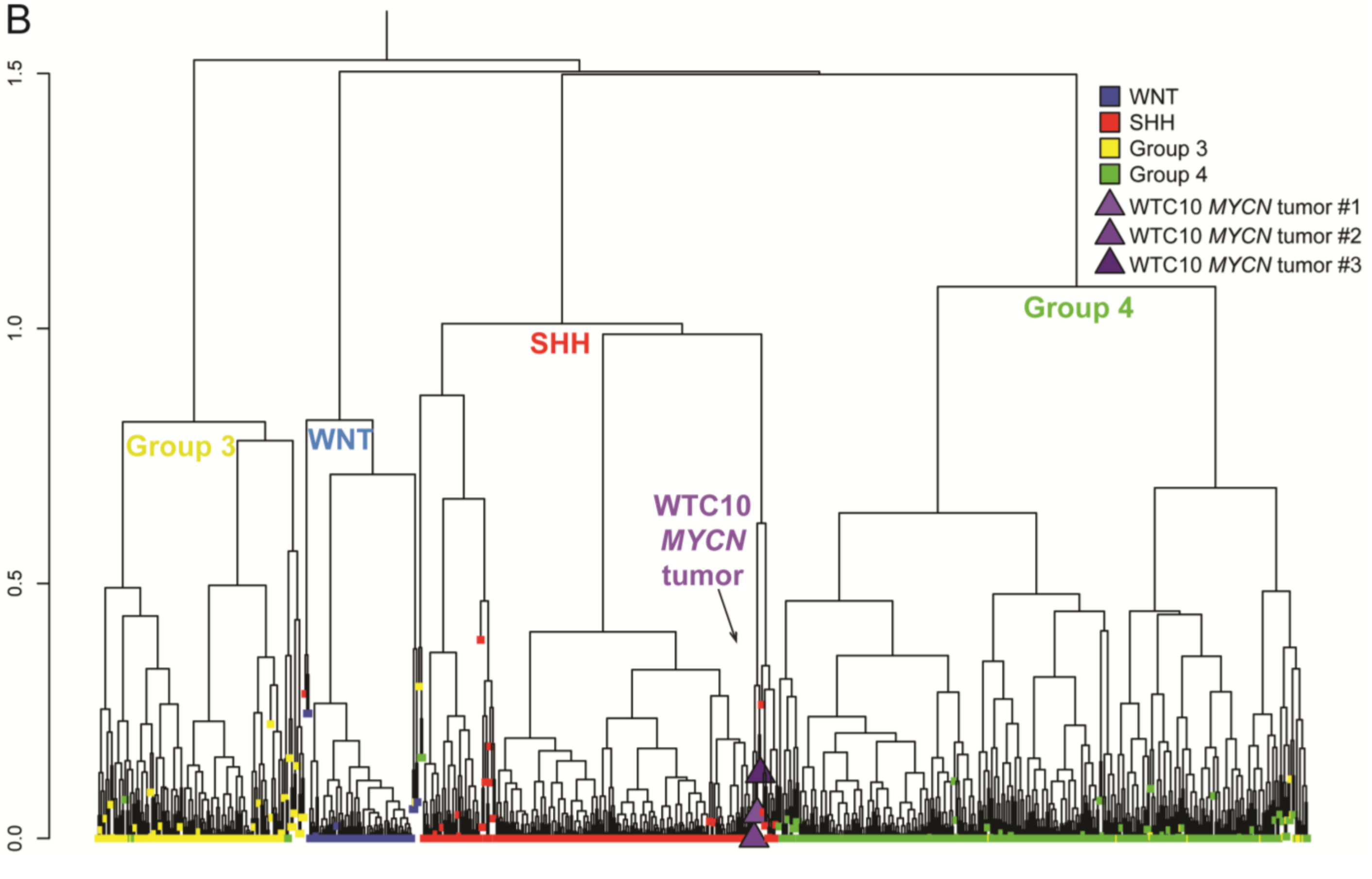

1.5
1.0
0.5
0.0
WNT
SHH
Group 3
Group 4
WTC10 *MYCN* tumor #1
WTC10 *MYCN* tumor #2
WTC10 *MYCN* tumor #3
Group 4
SHH
Group 3
WNT
WTC10
*MYCN*
tumor

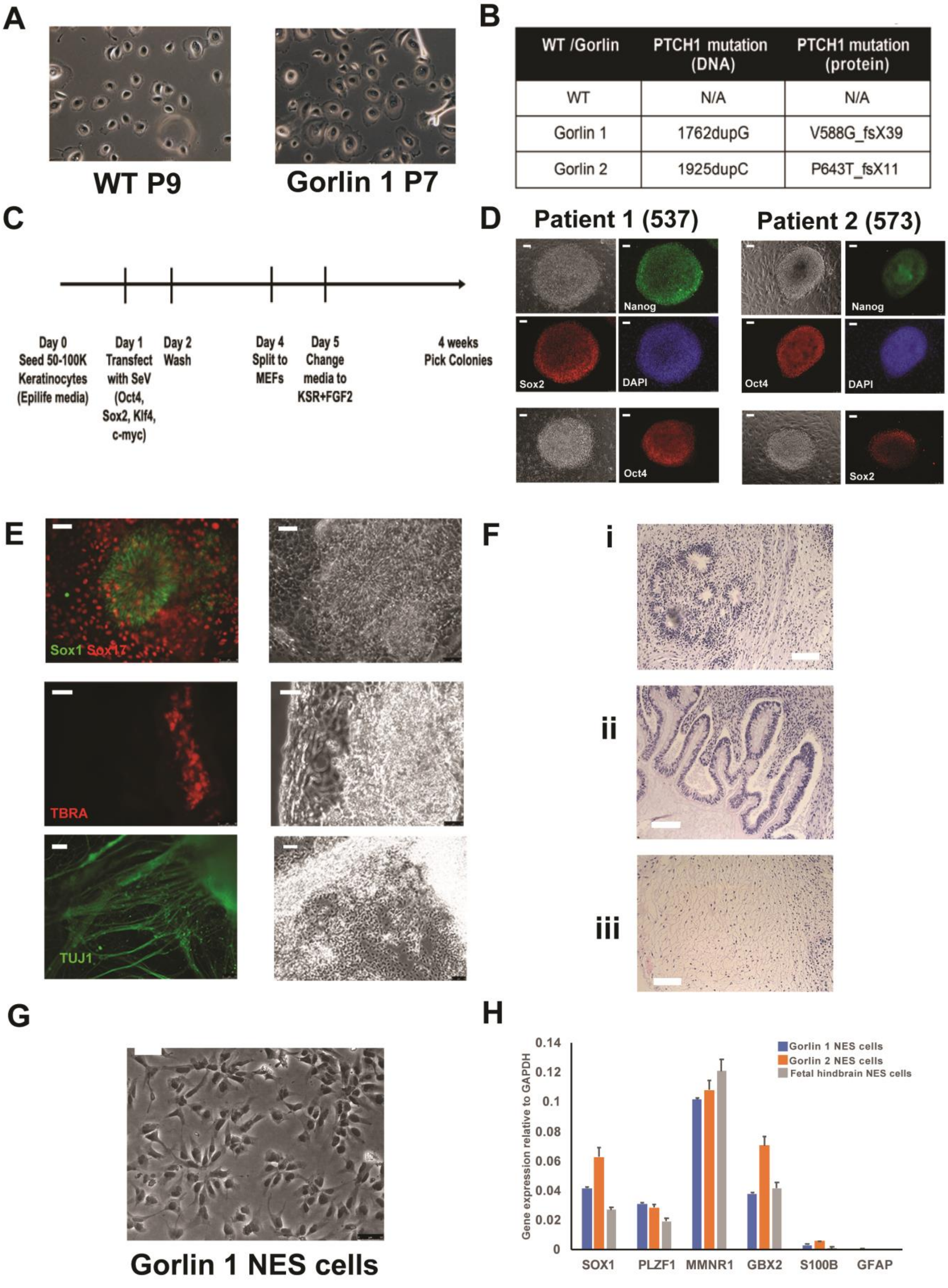


| WT /Gorlin | PTCH1 mutation (DNA) | PTCH1 mutation (protein) |
|---|---|---|
| WT | N/A | N/A |
| Gorlin 1 | 1762dupG | V588G_fsX39 |
| Gorlin 2 | 1925dupC | P643T_fsX11 |

Figure S3

# Figure S4

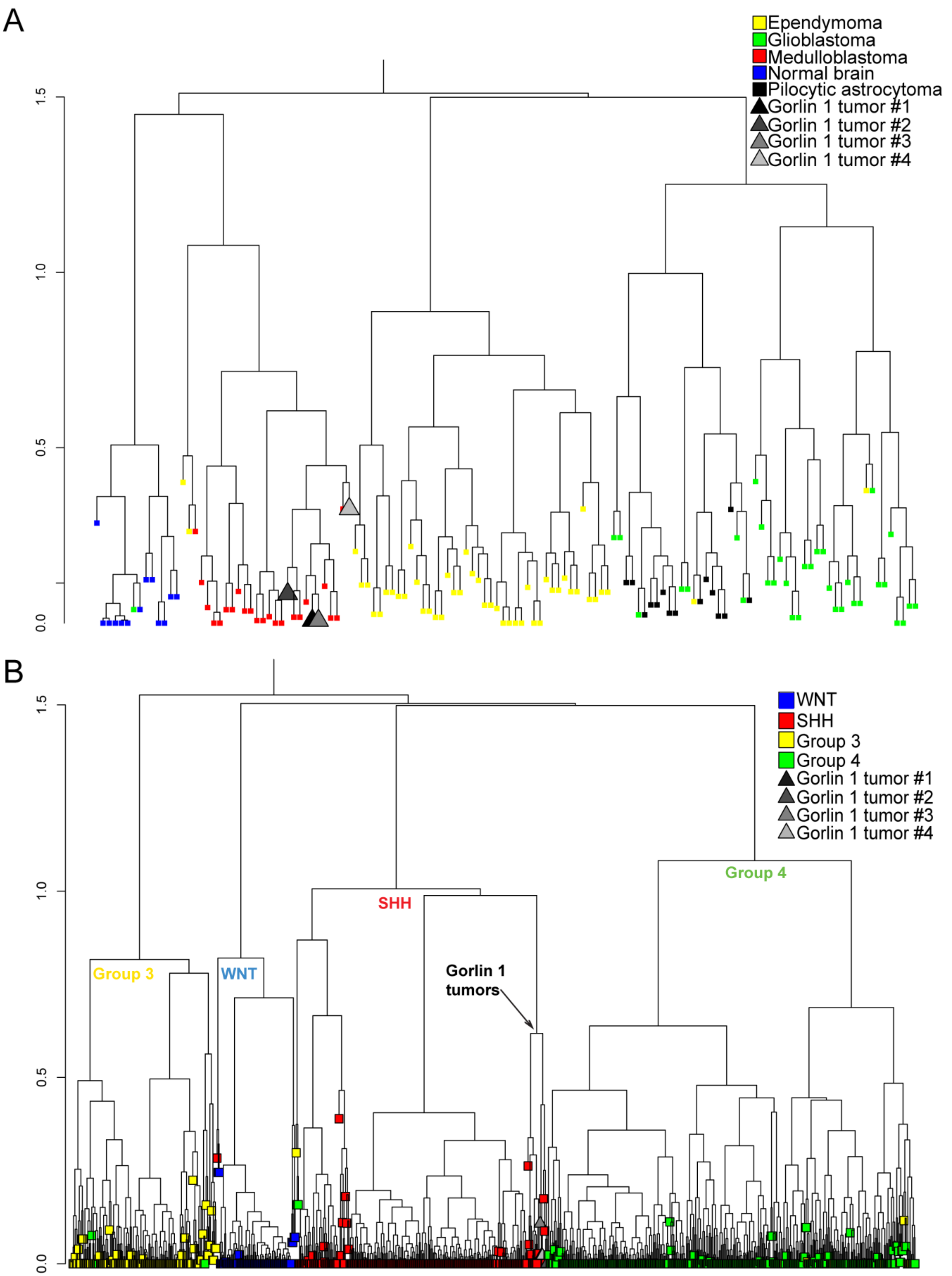
A
Ependymoma
Glioblastoma
Medulloblastoma
Normal brain
Pilocytic astrocytoma
Gorlin 1 tumor #1
Gorlin 1 tumor #2
Gorlin 1 tumor #3
Gorlin 1 tumor #4
1.5
1.0
0.5
0.0
B
WNT
SHH
Group 3
Group 4
Gorlin 1 tumor #1
Gorlin 1 tumor #2
Gorlin 1 tumor #3
Gorlin 1 tumor #4
Group 3
WNT
SHH
Gorlin 1 tumors
Group 4
1.5
1.0
0.5
0.0

Figure S5

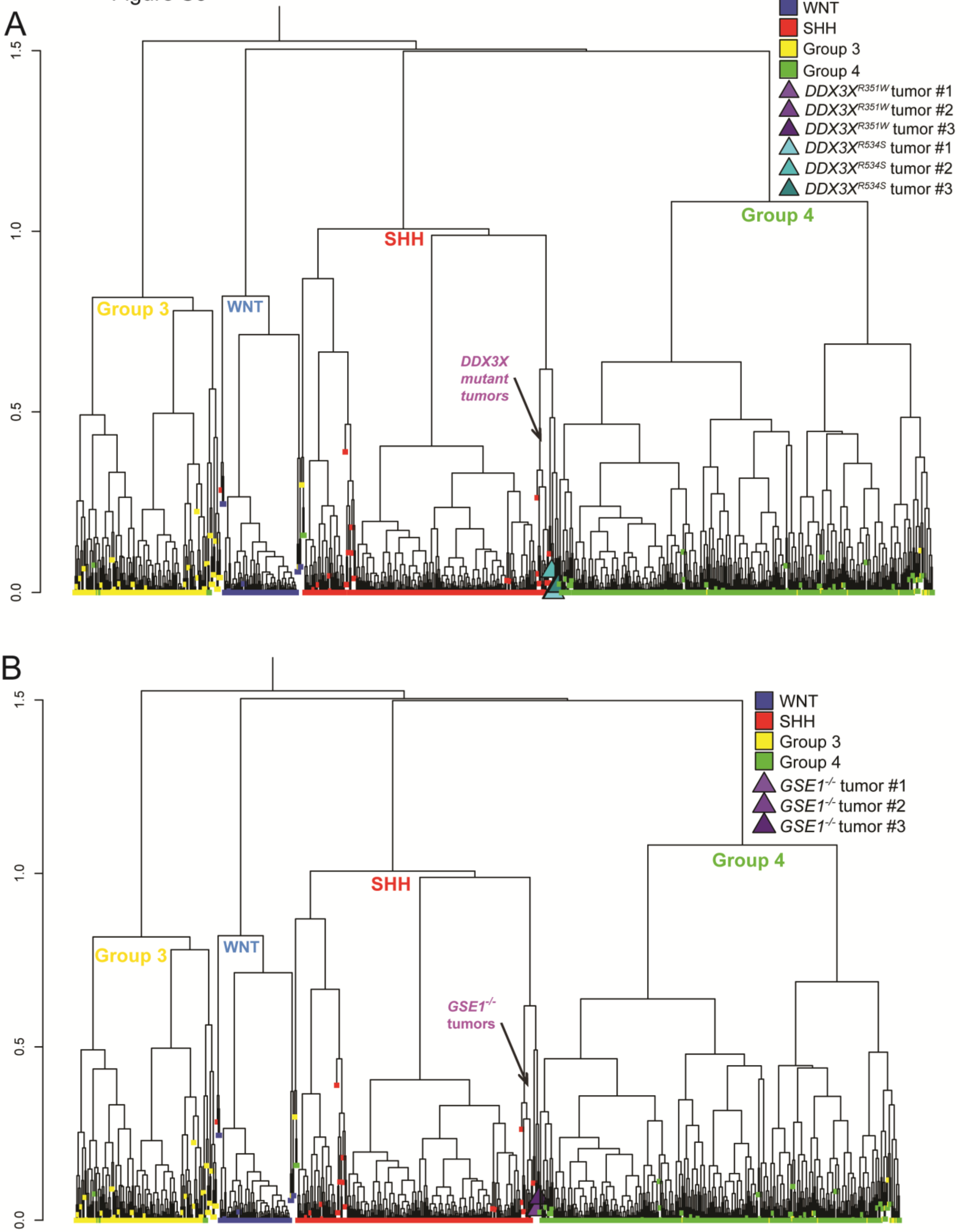
A
1.5
1.0
0.5
0.0
WNT
SHH
Group 3
Group 4
DDX3X^R351W tumor #1
DDX3X^R351W tumor #2
DDX3X^R351W tumor #3
DDX3X^R534S tumor #1
DDX3X^R534S tumor #2
DDX3X^R534S tumor #3
Group 4
SHH
Group 3
WNT
DDX3X mutant tumors
B
1.5
1.0
0.5
0.0
WNT
SHH
Group 3
Group 4
GSE1-/- tumor #1
GSE1-/- tumor #2
GSE1-/- tumor #3
Group 4
SHH
Group 3
WNT
GSE1-/- tumors

Figure S6

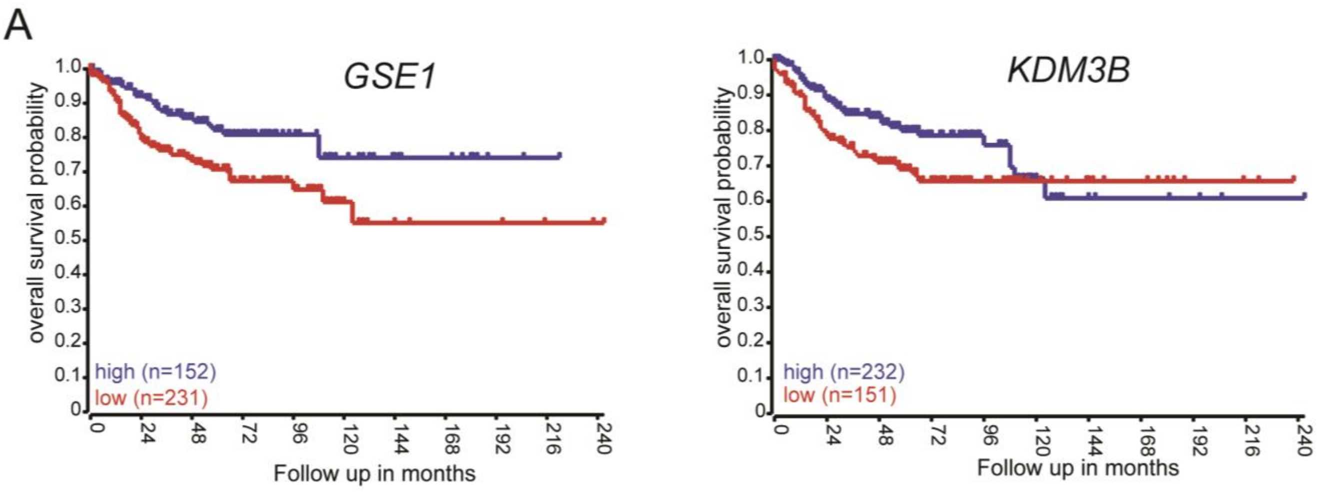


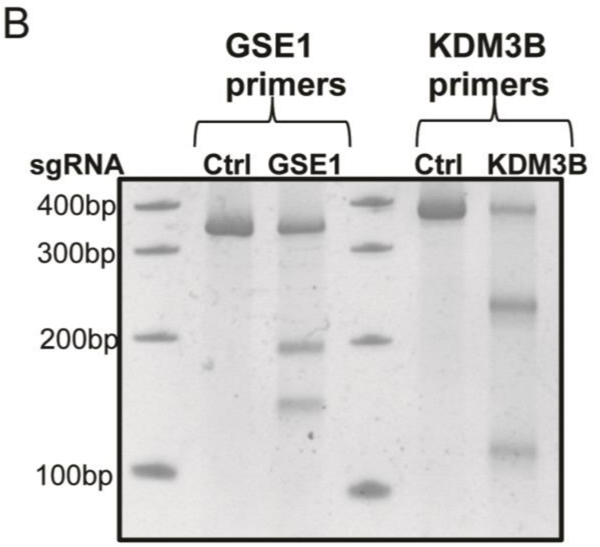


C

| Target gene | % mutated allele | % frameshift alleles |
| --- | --- | --- |
| *GSE1* | 99.2% | 87.9% |
| *KDM3B* | 96.7% | 69.8% |

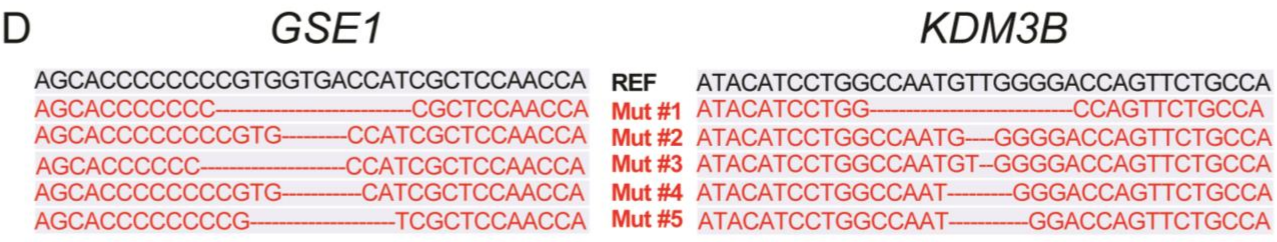


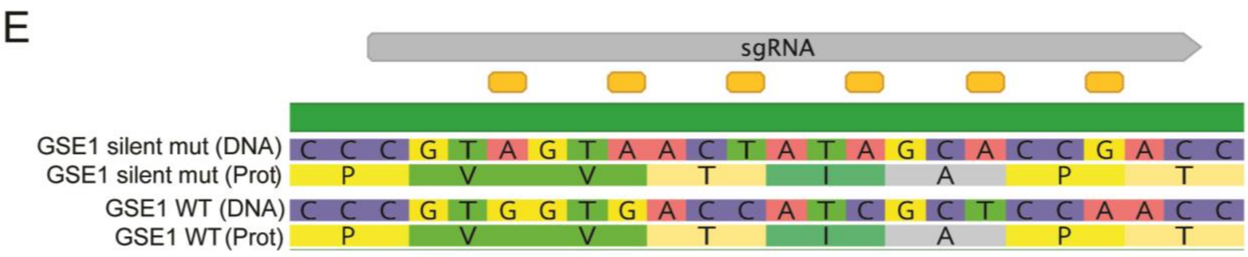

Figure S7

A

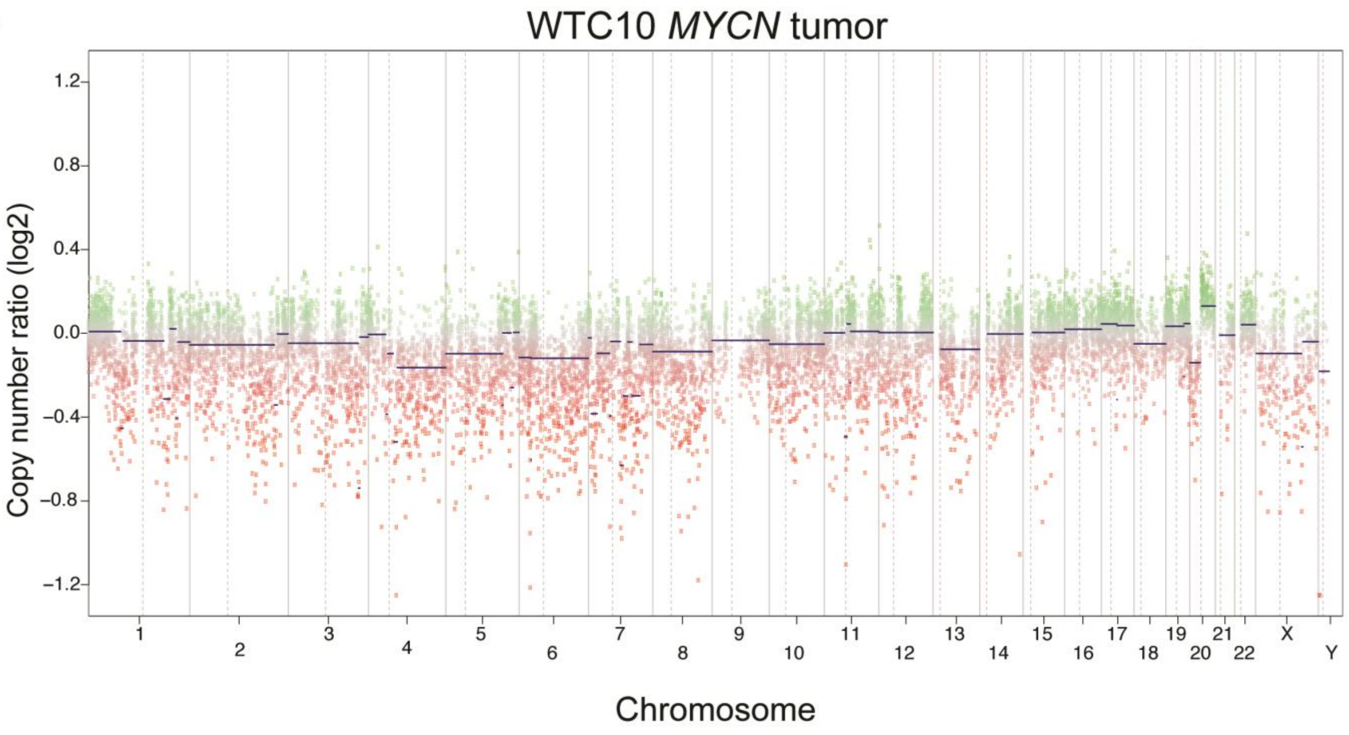

WTC10 *MYCN* tumor
Copy number ratio (log2)
1.2
0.8
0.4
0.0
−0.4
−0.8
−1.2
1 2 3 4 5 6 7 8 9 10 11 12 13 14 15 16 17 18 19 20 21 22 X Y
Chromosome


B

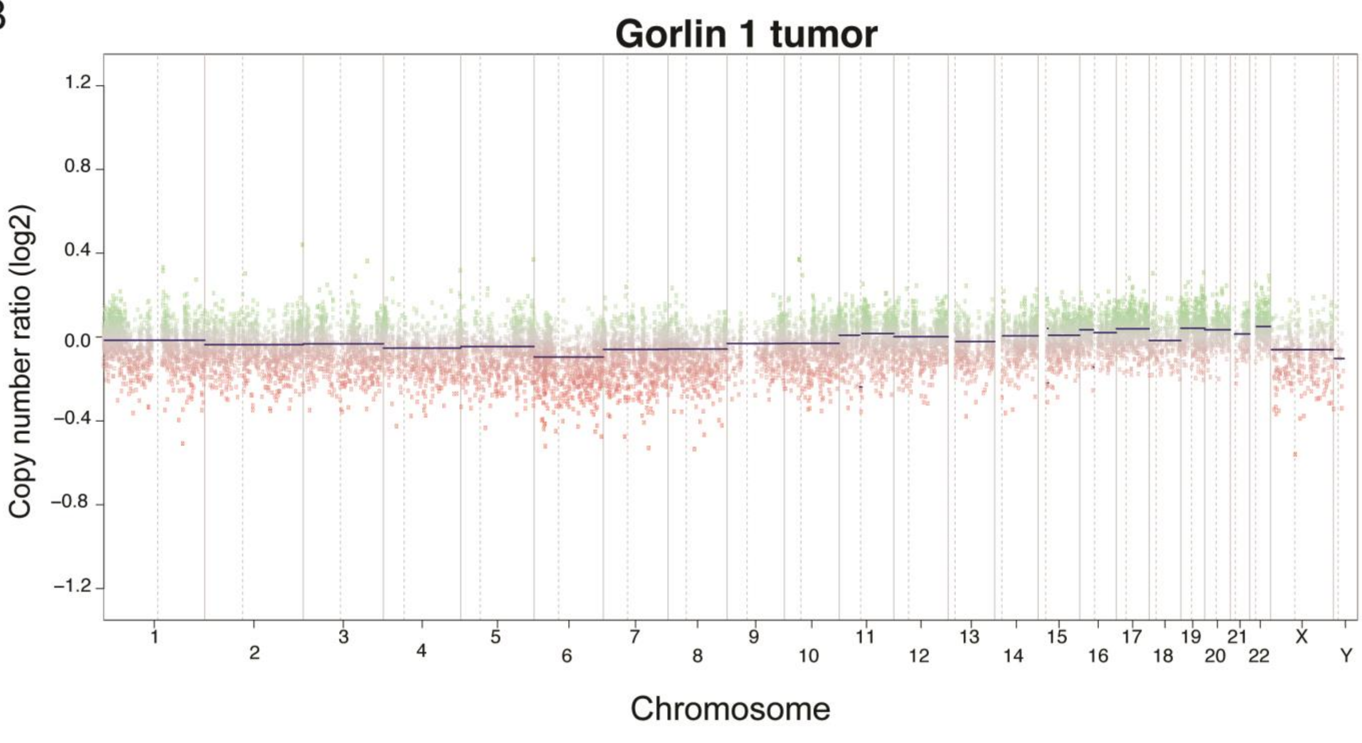

Gorlin 1 tumor
Copy number ratio (log2)
1.2
0.8
0.4
0.0
−0.4
−0.8
−1.2
1 2 3 4 5 6 7 8 9 10 11 12 13 14 15 16 17 18 19 20 21 22 X Y
Chromosome
